# Comparative transcriptomic and metabolic profiling provides insight into the mechanism by which the autophagy inhibitor 3-MA enhances salt stress sensitivity in wheat seedlings

**DOI:** 10.1186/s12870-021-03351-5

**Published:** 2021-12-06

**Authors:** Jieyu Yue, Yingjie Wang, Jinlan Jiao, Huazhong Wang

**Affiliations:** Tianjin Key Laboratory of Animal and Plant Resistance, Tianjin Normal University, Tianjin, 300387 China

**Keywords:** Wheat, Seedling growth, 3-Methyladenine, Salt stress, Metabolomics, Transcriptomics

## Abstract

**Background:**

Salt stress hinders plant growth and production around the world. Autophagy induced by salt stress helps plants improve their adaptability to salt stress. However, the underlying mechanism behind this adaptability remains unclear. To obtain deeper insight into this phenomenon, combined metabolomics and transcriptomics analyses were used to explore the coexpression of differentially expressed-metabolite (DEM) and gene (DEG) between control and salt-stressed wheat roots and leaves in the presence or absence of the added autophagy inhibitor 3-methyladenine (3-MA).

**Results:**

The results indicated that 3-MA addition inhibited autophagy, increased ROS accumulation, damaged photosynthesis apparatus and impaired the tolerance of wheat seedlings to NaCl stress. A total of 14,759 DEGs and 554 DEMs in roots and leaves of wheat seedlings were induced by salt stress. DEGs were predominantly enriched in cellular amino acid catabolic process, response to external biotic stimulus, regulation of the response to salt stress, reactive oxygen species (ROS) biosynthetic process, regulation of response to osmotic stress, ect. The DEMs were mostly associated with amino acid metabolism, carbohydrate metabolism, phenylalanine metabolism, carbapenem biosynthesis, and pantothenate and CoA biosynthesis. Further analysis identified some critical genes (gene involved in the oxidative stress response, gene encoding transcription factor (TF) and gene involved in the synthesis of metabolite such as alanine, asparagine, aspartate, glutamate, glutamine, 4-aminobutyric acid, abscisic acid, jasmonic acid, ect.) that potentially participated in a complex regulatory network in the wheat response to NaCl stress. The expression of the upregulated DEGs and DEMs were higher, and the expression of the down-regulated DEGs and DEMs was lower in 3-MA-treated plants under NaCl treatment.

**Conclusion:**

3-MA enhanced the salt stress sensitivity of wheat seedlings by inhibiting the activity of the roots and leaves, inhibiting autophagy in the roots and leaves, increasing the content of both H_2_O_2_ and O_2_•—, damaged photosynthesis apparatus and changing the transcriptome and metabolome of salt-stressed wheat seedlings.

**Supplementary Information:**

The online version contains supplementary material available at 10.1186/s12870-021-03351-5.

## Background

Global climate change, high levels of fertilizer application and improper irrigation methods make global soil salinization increasingly serious [1, 2]. Worldwide, no less than 800 million hectares of arable land have been salinized, and the area of salinized soil is still increasing every year [3]. NaCl is the most soluble and abundant salt released into soil among all types of salt [4]. Under salt stress, plants suffer from water deficiency, osmotic stress, ion toxicity, and oxidative damage, which consequently induce an ion imbalance, physiological metabolism disorders, blocked protein synthesis, the accumulation of toxic substances, and the accelerated senescence and death of plants [2, 5, 6]. In the evolutionary process, plants have evolved various defense mechanisms to cope with salt stress [7]. Previous studies have shown that the salt tolerance of plants involves many genes, including those involved in ion transport, cell defense, physiological metabolism and cell growth, and synergistic effects can be achieved through multiple mechanisms, such as compartmentalization of Na^+^ into vacuoles by ion transport or the reduction of oxidative damage [6, 8]. It is of great importance to elucidate the underlying mechanisms behind salt stress tolerance in plants.

Autophagy is a defense mechanism in plants that can be activated by salt stress [9]. Autophagy helps maintain physiological homeostasis by capturing dysfunctional or obsolete organelles and proteins in distinct double-membraned vesicles called autophagosomes and delivering these cargos to vacuoles for degradation [9–11]. Luo et al. (2017) found that autophagy induced by salt stress was able to enhance the salt tolerance of *Arabidopsis thaliana* [12]. Zhou et al. (2018) found that the addition of 3-methyladenine (3-MA) enhanced the number of cells undergoing programmed cell death (PCD) in TMV-infected tomato root-tips via the inhibition of autophagy [13]. As a mature autophagy inhibitor, 3-MA efficiently inhibited of phosphatidylinositol 3-kinase (PI3K) and disrupted intracellular protein degradation without interfering intracellular protein synthesis, as the proteins degradation is carried out through endocytosis and affects intracellular ATP levels [14, 15]. Baena et al. (2021) found that the inhibition of autophagy via 3-MA increased the monoubiquitination of nonphotosynthetic phosphoenolpyruvate carboxylase in *Arabidopsis* [16]*.* Overexpression of *MdATG10* or *MdATG8i* helped apple plants better adapt to salt stress, by alleviating the decrease in carbon assimilation and the accumulation of compatible osmolytes [3, 17]. Salt stress usually induces excess reactive oxygen species (ROS) in plant, which acts as a link between autophagy and abiotic stresses [9, 18]. ROS can induce autophagy and in turn control ROS overproduction by promoting the degradation of damaged organelles [9, 18, 19], but the specific mechanism is unclear. Moreover, current research on autophagic functions in plant resistance has mainly focused on model plants, and the molecular mechanism by which autophagy regulates crop plant responses to salt stress is still not well defined.

With the advancement of molecular biology and high-throughput sequencing technologies, plant stress research has entered an era of functional omics characterized by genomics, transcriptomics, proteomics, metabolomics, and comparative genomics [20–23]. Transcriptome sequencing can dynamically detect the expression changes of plant genes at different times and in different locations under stress in real time, exploit novel functional genes, and lay the foundation for revealing the regulatory mechanism of the plant response to stress [24–27]. At present, researchers have used high-throughput RNA sequencing (RNA-seq) to analyze the molecular mechanisms of wheat, rice, maize and other plants in response to drought, waterlogging, salinization, heavy metals and other stresses [23, 24, 28–30]. Metabonomics is a new science discipline that was developed after genomics, transcriptomics and proteomics. It can be used to systematically and quantitatively analyze the global metabonome in an organism or cell under external stimuli [31, 32]. It is considered a bridge between the plant genome and plant phenome [33, 34]. Many key metabolites in plants were found to regulate various metabolic activities to induce tolerance under diverse biotic and abiotic stresses [35–37]. The accumulation of both vanillic acid and phydroxybenzoic acid in rice significantly increased drought stress and was proportional to the drought resistance levels of rice [38]. Amino acids and their derivatives help plants adapt to salt stress [39]. Fatty acids play a key role in plant resistance to heavy metals [40].

The combined analysis of metabolomics and transcriptomics can improve the accuracy of investigations into biological problems and can more accurately explain the expression patterns of key functional genes and the pathways they participate in [5, 41–43]. Accordingly, the molecular functions and regulatory mechanisms of crop stress tolerance will be understood by identifying the key metabolic pathways or genes and metabolites [42]. Zhang et al. (2019) found that amino acid metabolism and sucrose and raffinose family oligosaccharide metabolism were the key pathways involved in rapid adaptive responses to salt stress in salt-tolerant sesame genotypes [5]. Guo et al. (2019) comparatively investigated the dynamic metabolomic and transcriptomic profiles of two transdifferentiation processes, embryogenic differentiation and somatic embryo development, during the somatic embryogenesis process in cotton, and identified a series of potential metabolites and corresponding genes responsible for somatic embryogenesis transdifferentiation [44].

The important crop wheat has a large, complicated and hexaploid genome [45]. Unraveling the complex defense mechanism of crop plants to salt stress at the metabolome and transcriptome levels is extremely important. Our previous study demonstrated that autophagy in the roots and leaves of wheat was induced by salt stress and contributed to wheat adaptation to salt stress [9]. To better understand the involvement of autophagy in the wheat response to salt stress, an association analysis of metabolomics and transcriptomics was used to investigate changes in various metabolites and fundamental regulatory pathways of wheat seedlings through the addition of 3-MA under normal or salt stress conditions. The results will help systematically reveal the mechanisms of wheat adaptation and tolerance to salt stress.

## Results

### 3-MA inhibits autophagy, increases ROS accumulation and impairs the tolerance of wheat seedlings to NaCl stress

**Fig. 1:**
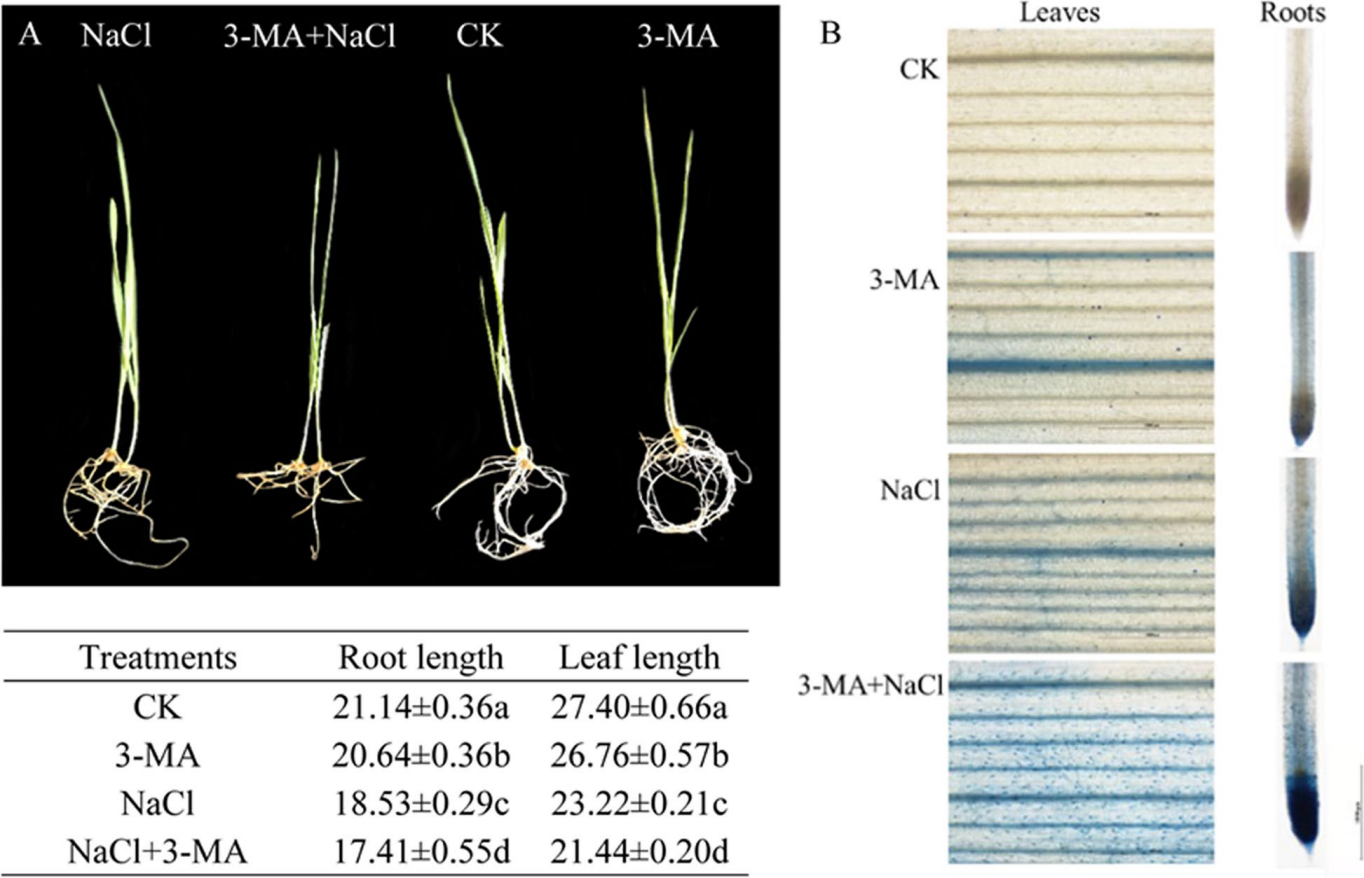
The effect of 3-MA on the growth of wheat seedlings under 150 mM NaCl stress. **A** was the effect of 3-MA on the growth and related physiological indexes of wheat seedlings under NaCl stress. The data are shown as mean ± SD of three independent experiments. The data with different capital letters in same column show significant difference (*P* < 0.05). **B** was the effect of 3-MA on the activity of roots and leaves of wheat seedling examined by Evans blue staining (scale was 1 mm)

**Fig. 2:**
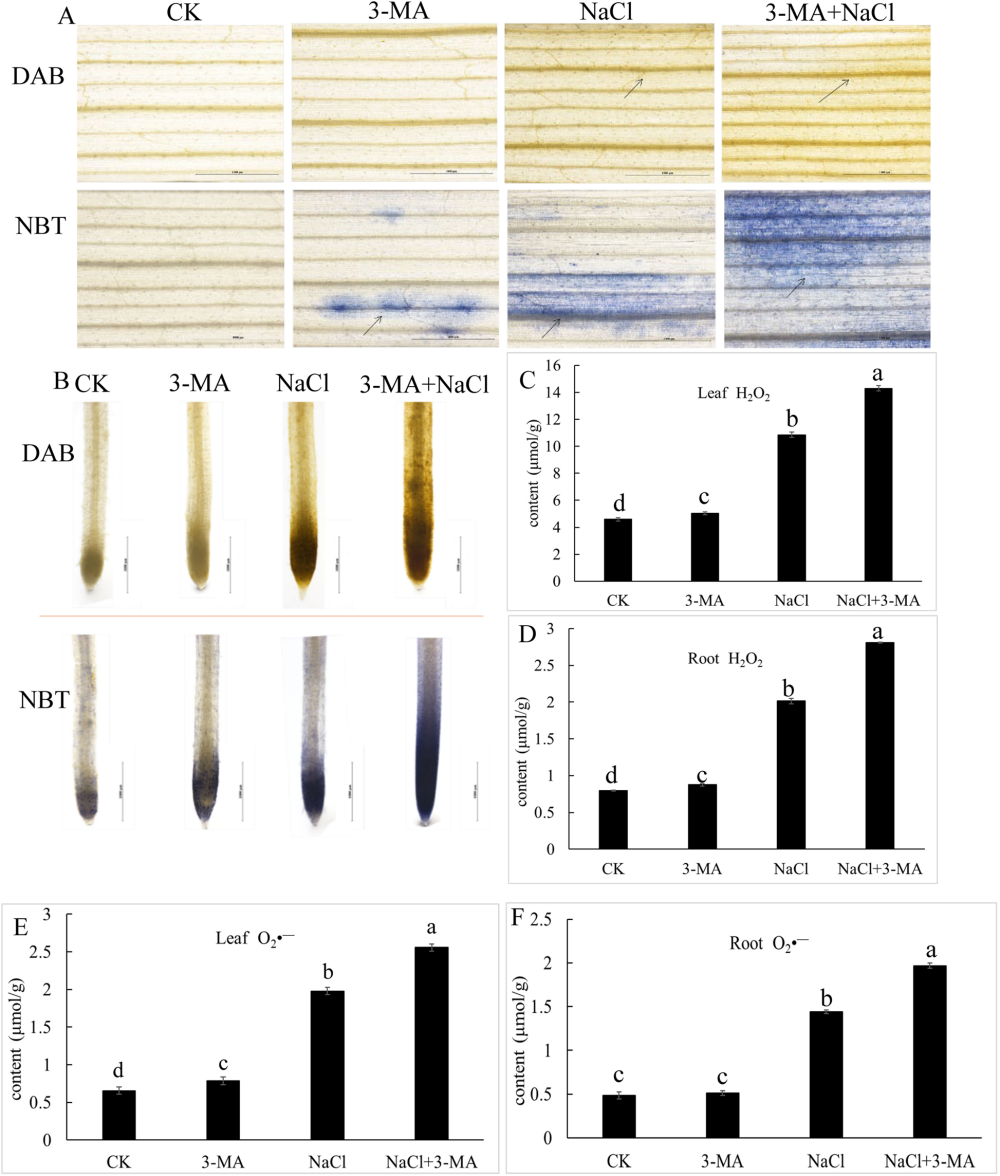
The effect of 3-MA on the ROS accumulation in roots and leaves of wheat seedlings under 150 mM NaCl stress. **A** The accumulation of H_2_O_2_ and O_2_•^—^ in leaves of wheat seedlings stained by DAB and NBT. **B** The accumulation of H_2_O_2_ and O_2_•^—^ in roots of wheat seedlings stained by DAB and NBT. **C** and **D** The content of H_2_O_2_ in leaves and roots of wheat seedlings. **E** and **F** The content of O_2_•^—^ in leaves and roots of wheat seedlings. All of the experiments presented here were performed at least 3 times, and similar results were obtained. Bars with different letters are significantly different at *P* < 0.05

Under normal conditions, exogenous 3-MA had a little effect on plant growth (Fig. 1 A) or the activity (Fig. 1 B) or content of hydrogen peroxide (H_2_O_2_) and superoxide (O_2_•—) in both the roots and leaves of wheat seedlings (Fig. 2). NaCl stress significantly reduced root and leaf length, decreased root and leaf activity, increased the accumulation of O_2_•— and H_2_O_2_ and enhanced the accumulation of autophagosomes (Supplementary Fig. 1) in the roots and leaves of wheat seedlings. 3-MA addition significantly reduced root and leaf length, decreased the activity of root and leaves, increased the content of both H_2_O_2_ and O_2_•^—^ and decreased the autophagic activity with a lower number of MDC-stained autophagosomes in the roots and leaves of wheat seedlings under NaCl stress. This visual observation of O_2_•^—^ and H_2_O_2_ matched the quantitative results (content of O_2_•^—^ and H_2_O_2_).

Several chlorophyll fluorescence parameters reflecting the health or integrity of the photosynthesis apparatus in the leaves of seedlings were measured. As compared with the NaCl-treated wheat seedlings, the 3-MA treated seedlings had significantly lower values of PSII photochemistry (Fv/Fm), quantum yield of PSII (Y (II)), quenching coefficient (qP), and electron transfer rate (ETR) while a significantly higher value of nonphotochemical quenching coefficient (NPQ) (Table 1). 3-MA addition aggravated damage to PSII of wheat seedlings induced by NaCl stress. The results suggested that 3-MA addition aggravated the damage to the of roots and leaves of wheat seedlings induced by NaCl stress.

**Table 1:** The effect of 3-MA on the chlorophyll fluorescence parameters in leaves of NaCl-treated wheat seedlings

Treatments	Fv/Fm	Y (II)	qP	NPQ	ETR
CK	0.80 ± 0.01a	0.35 ± 0.03a	0.56 ± 0.02a	0.56 ± 0.00d	52.64 ± 0.53a
3-MA	0.78 ± 0.02b	0.33 ± 0.03b	0.53 ± 0.03b	0.62 ± 0.01c	48.80 ± 0.33b
NaCl	0.75 ± 0.00c	0.22 ± 0.02c	0.38 ± 0.04c	1.24 ± 0.00b	38.26 ± 0.26c
NaCl+ 3-MA	0.71 ± 0.01d	0.18 ± 0.01d	0.32 ± 0.03d	1.53 ± 0.04a	35.50 ± 0.57d

The data are shown as mean ± SD of three independent experiments. The data with different capital letters in same column show significant difference (*P <* 0.05)

### Transcriptome analysis

**Fig. 3:**
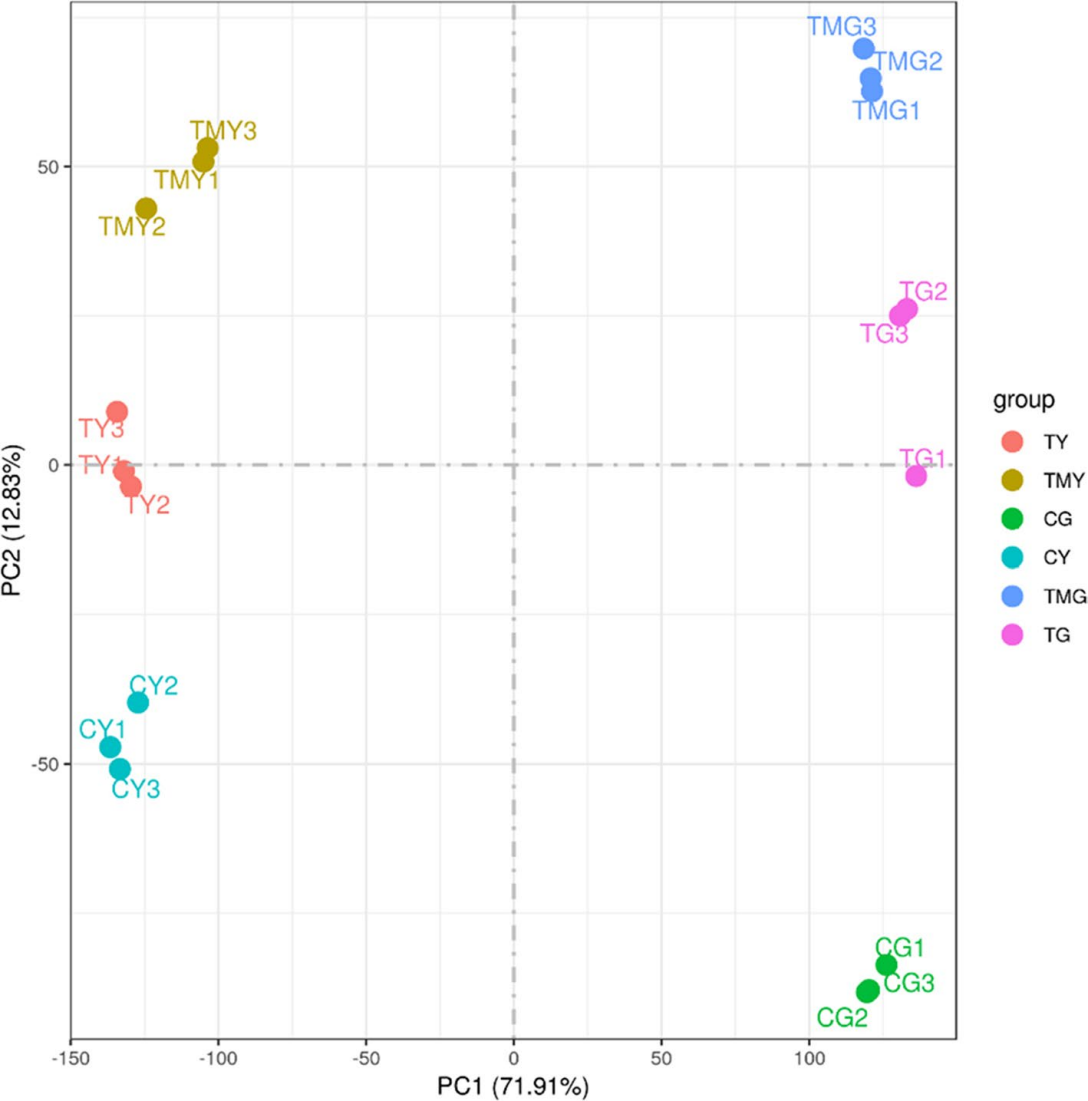
Principal component analysis (PCA) clustering based on RNA-Seq data. Note: CG: the control wheat roots, TG: 150 mM NaCl treated wheat roots, TMG: 5 mM 3-MA + 150 mM NaCl treated wheat roots, CY: the control wheat leaves, TY: 150 mM NaCl treated wheat leaves, TMY: 5 mM 3-MA + 150 mM NaCl treated wheat leaves

The RNA-seq data analysis was reliable and had high quality. The biological replicates of each treatment had good correlation (*R*^*2*^ > 0.92) (Supplementary Fig. 2), which indicated that the three biological replicates had good repeatability. Based on principal component analysis (PCA), a clear separation between the NaCl-treated group and controls could be observed (Fig. 3). The average GC content of the RNA-seq reads was 55.46% (Table 2), and the Q30 score (sequences with sequencing error rates lower than 0.1%) was over 94%. Each library obtained 68,310,810–83,844,286 high-quality clean reads by removing the low-quality sequences and adaptor sequences. Approximately 93.6–95.9% of these high-quality clean reads were mapped to the wheat reference genome (Table 3). A total of 120,744 expressed genes (with FPKM > 0) were predicted from the wheat genome, including 25,180 annotated genes.

**Table 2:** Summary of RNA-Seq data

Sample	Library	Raw_reads	Clean_reads	Clean_bases	Error_rate	Q20	Q30	GC_pct
CG1	FRAS202073351-1a	77,255,216	75,523,830	11.33G	0.02	98.43	95.14	54.4
CG2	FRAS202073352–1a	70,294,604	68,220,606	10.23G	0.02	98.38	95	54.75
CG3	FRAS202073353-1a	68,310,810	66,534,384	9.98G	0.02	98.48	95.24	55.48
CY1	FRAS202073355-1a	68,432,734	67,194,008	10.08G	0.02	98.39	95.02	56.18
CY2	FRAS202073356-1a	70,719,360	69,011,564	10.35G	0.02	98.37	95.02	56.36
CY3	FRAS202073357-1a	79,082,316	76,966,138	11.54G	0.02	98.31	94.83	57.38
TG1	FRAS202073359-1a	80,299,962	79,055,488	11.86G	0.02	98.3	94.84	54.8
TG2	FRAS202073360-1a	75,881,502	74,512,988	11.18G	0.02	98.37	94.99	54.64
TG3	FRAS202073361-1a	73,538,600	72,202,420	10.83G	0.02	98.33	94.92	54.89
TY1	FRAS202073363-1a	76,023,686	74,889,566	11.23G	0.02	98.23	94.65	56.01
TY2	FRAS202073364-1a	78,758,494	77,610,338	11.64G	0.02	98.29	94.78	56.36
TY3	FRAS202073365-1a	75,182,950	74,136,986	11.12G	0.02	98.25	94.69	56.05
TMG1	FRAS202073367-1a	74,723,446	73,524,748	11.03G	0.02	98.29	94.82	54.87
TMG2	FRAS202073368-1a	83,361,782	81,966,850	12.3G	0.02	98.31	94.81	54.8
TMG3	FRAS202073369-1a	77,013,812	75,163,830	11.27G	0.02	98.28	94.76	54.71
TMY1	FRAS202073371-1a	83,844,286	82,055,564	12.31G	0.02	98.17	94.54	55.58
TMY2	FRAS202073372–1a	76,328,120	74,958,466	11.24G	0.02	98.25	94.73	55.74
TMY3	FRAS202073373-1a	68,342,108	66,789,756	10.02G	0.02	98.27	94.76	55.35

**Table 3:** The mapping percentage of the reads to the reference genome

Sample	Total_reads	Total_map	Unique_map	Multi_map	Read1_map	Read2_map	Positive_map	Negative_map	Splice_map	Unsplice_map
CG1	75,523,830	71,664,697 (94.89%)	68,307,785 (90.45%)	3,356,912 (4.44%)	34,199,117 (45.28%)	34,108,668 (45.16%)	34,148,669 (45.22%)	34,159,116 (45.23%)	21,179,274 (28.04%)	47,128,511 (62.4%)
CG2	68,220,606	64,924,770 (95.17%)	61,732,477 (90.49%)	3,192,293 (4.68%)	30,922,970 (45.33%)	30,809,507 (45.16%)	30,864,438 (45.24%)	30,868,039 (45.25%)	21,112,879 (30.95%)	40,619,598 (59.54%)
CG3	66,534,384	63,310,965 (95.16%)	60,365,460 (90.73%)	2,945,505 (4.43%)	30,216,035 (45.41%)	30,149,425 (45.31%)	30,186,963 (45.37%)	30,178,497 (45.36%)	20,301,179 (30.51%)	40,064,281 (60.22%)
TG1	79,055,488	74,411,921 (94.13%)	71,278,205 (90.16%)	3,133,716 (3.96%)	35,716,283 (45.18%)	35,561,922 (44.98%)	35,629,244 (45.07%)	35,648,961 (45.09%)	21,666,142 (27.41%)	49,612,063 (62.76%)
TG2	74,512,988	69,747,227 (93.6%)	66,309,895 (88.99%)	3,437,332 (4.61%)	33,215,753 (44.58%)	33,094,142 (44.41%)	33,143,538 (44.48%)	33,166,357 (44.51%)	22,766,624 (30.55%)	43,543,271 (58.44%)
TG3	72,202,420	67,995,364 (94.17%)	64,590,239 (89.46%)	3,405,125 (4.72%)	32,349,299 (44.8%)	32,240,940 (44.65%)	32,287,468 (44.72%)	32,302,771 (44.74%)	22,532,341 (31.21%)	42,057,898 (58.25%)
TMG1	73,524,748	69,635,427 (94.71%)	66,016,287 (89.79%)	3,619,140 (4.92%)	33,077,821 (44.99%)	32,938,466 (44.8%)	32,995,555 (44.88%)	33,020,732 (44.91%)	23,315,017 (31.71%)	42,701,270 (58.08%)
TMG2	81,966,850	77,512,447 (94.57%)	73,592,660 (89.78%)	3,919,787 (4.78%)	36,881,779 (45.0%)	36,710,881 (44.79%)	36,782,914 (44.88%)	36,809,746 (44.91%)	26,237,150 (32.01%)	47,355,510 (57.77%)
TMG3	75,163,830	70,955,569 (94.4%)	67,303,940 (89.54%)	3,651,629 (4.86%)	33,733,352 (44.88%)	33,570,588 (44.66%)	33,642,864 (44.76%)	33,661,076 (44.78%)	24,476,380 (32.56%)	42,827,560 (56.98%)
CY1	67,194,008	64,359,545 (95.78%)	61,153,519 (91.01%)	3,206,026 (4.77%)	30,639,976 (45.6%)	30,513,543 (45.41%)	30,555,311 (45.47%)	30,598,208 (45.54%)	20,261,507 (30.15%)	40,892,012 (60.86%)
CY2	69,011,564	66,026,437 (95.67%)	62,452,186 (90.5%)	3,574,251 (5.18%)	31,278,820 (45.32%)	31,173,366 (45.17%)	31,206,020 (45.22%)	31,246,166 (45.28%)	22,121,279 (32.05%)	40,330,907 (58.44%)
CY3	76,966,138	73,812,694 (95.9%)	69,582,215 (90.41%)	4,230,479 (5.5%)	34,875,194 (45.31%)	34,707,021 (45.09%)	34,761,852 (45.17%)	34,820,363 (45.24%)	24,463,569 (31.78%)	45,118,646 (58.62%)
TY1	74,889,566	71,521,444 (95.5%)	68,026,253 (90.84%)	3,495,191 (4.67%)	34,100,291 (45.53%)	33,925,962 (45.3%)	33,994,689 (45.39%)	34,031,564 (45.44%)	24,893,596 (33.24%)	43,132,657 (57.6%)
TY2	77,610,338	74,187,300 (95.59%)	70,702,863 (91.1%)	3,484,437 (4.49%)	35,434,985 (45.66%)	35,267,878 (45.44%)	35,335,115 (45.53%)	35,367,748 (45.57%)	26,023,123 (33.53%)	44,679,740 (57.57%)
TY3	74,136,986	70,791,930 (95.49%)	67,420,458 (90.94%)	3,371,472 (4.55%)	33,793,751 (45.58%)	33,626,707 (45.36%)	33,689,433 (45.44%)	33,731,025 (45.5%)	25,071,938 (33.82%)	42,348,520 (57.12%)
TMY1	82,055,564	78,281,544 (95.4%)	74,255,728 (90.49%)	4,025,816 (4.91%)	37,238,423 (45.38%)	37,017,305 (45.11%)	37,103,907 (45.22%)	37,151,821 (45.28%)	26,675,332 (32.51%)	47,580,396 (57.99%)
TMY2	74,958,466	71,597,070 (95.52%)	67,847,161 (90.51%)	3,749,909 (5.0%)	34,008,689 (45.37%)	33,838,472 (45.14%)	33,900,507 (45.23%)	33,946,654 (45.29%)	25,189,516 (33.6%)	42,657,645 (56.91%)
TMY3	66,789,756	63,811,203 (95.54%)	60,607,358 (90.74%)	3,203,845 (4.8%)	30,376,657 (45.48%)	30,230,701 (45.26%)	30,283,505 (45.34%)	30,323,853 (45.4%)	21,176,707 (31.71%)	39,430,651 (59.04%)

### Transcriptome profiling of wheat roots and leaves in response to NaCl stress at the seedling stage

At least 14,759 genes showed significant differences among the comparisons of seedlings induced by NaCl stress (Table 4). The functions of these DEGs were further predicted on the basis of their associated annotations. After NaCl stress, compared with the control, 6569 DEGs were upregulated and 9712 DEGs were down-regulated in the leaves, and 13,261 DEGs were upregulated and of 13,692 DEGs were downregulated in the roots (*P* < 0.05 and |log2FoldChange| > 0). The DEGs in the roots were annotated using Gene Ontology (GO) analysis and the results showed that the top clusters of DEGs belonged to ‘phenylpropanoid metabolic process,’ ‘aromatic amino acid family metabolic process’ and ‘secondary metabolic process’ in the category of biological process. They grouped into ‘apoplast,’ ‘photosynthetic membrane’ and ‘thylakoid part’ in the cellular component category and ‘manganese ion binding’, ‘NAD binding’ and ‘nutrient reservoir activity’ in the molecular function category (Supplementary Fig. 3A). The DEGs in the roots were also annotated using GO analysis and the results showed that top clusters of DEGs belong to ‘photosynthesis’, ‘photosynthesis, light reaction’ and ‘photosynthesis, light harvesting’ in the biological process category. They grouped into ‘chloroplast stroma,’ ‘photosynthetic membrane’ and ‘thylakoid membrane’ in the cellular component category and ‘carbon-carbon lyase activity’, ‘channel activity’ and ‘passive transmembrane transporter activity’ in the molecular function category (Supplementary Fig. 3B). Furthermore, the GO terms of the DEGs in the roots were aggregated in responses to salt stress, including response to biotic stimulus (GO:0009607), response to antibiotics (GO:0046677), response to wounding (GO:0009611), ect. (Supplementary Table S1). The GO terms of the DEGs in the leaves were also aggregated in responses to various stresses, such as response to osmotic stress (GO:0006970), response to salt stress (GO:0009651), response to superoxide (GO:0000303), ect. (Supplementary Table S2).

**Table 4:** The DEGs among the comparisons

compare	all	up	down	threshold
TGvsCG	26,953	13,261	13,692	DESeq2 padj<=0.05 |log2FoldChange| > =0.0
TMGvsTG	16,482	6587	9895	DESeq2 padj<=0.05 |log2FoldChange| > =0.0
TYvsCY	16,281	6569	9712	DESeq2 padj<=0.05 |log2FoldChange| > =0.0
TMYvsTY	14,759	7194	7565	DESeq2 padj<=0.05 |log2FoldChange| > =0.0

**Fig. 4:**
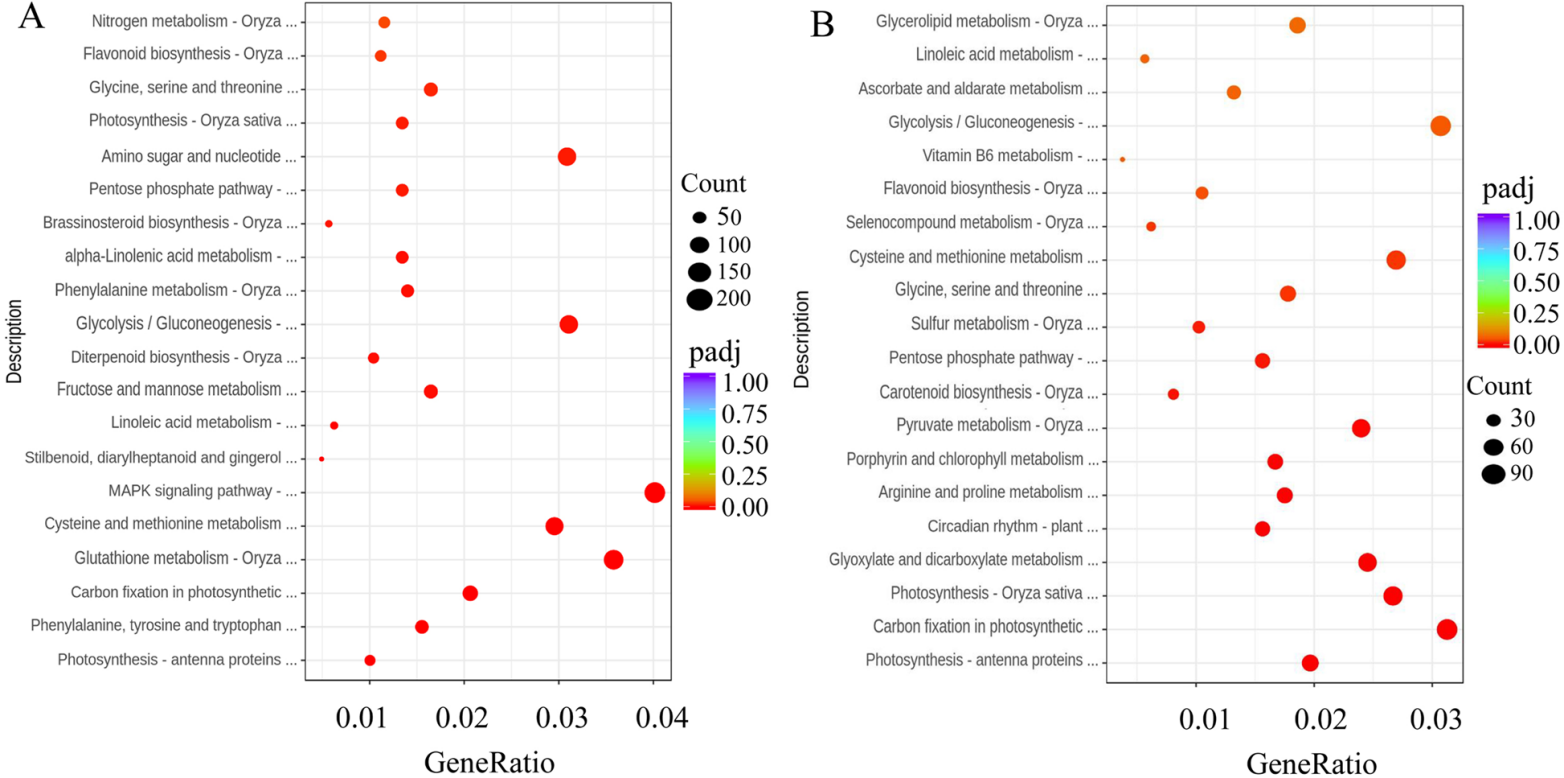
The differentially expressed genes (DEGs) with Kyoto Encyclopedia of Genes and Genomes (KEGG) pathway enrichment (top 20) in wheat roots (**A**) and leaves (**B**) in response to salt stress. Note: CG: the control wheat roots, TG: 150 mM NaCl treated wheat roots, CY: the control wheat leaves, TY: 150 mM NaCl treated wheat leaves

The Kyoto Encyclopedia of Genes and Genomes (KEGG) pathway was used to annotate the DEGs in the roots and leaves. The DEGs in roots were mainly enriched in ‘MAPK signaling pathway’ (212, 4%), ‘Glutathione metabolism’ (189, 3.5%) and ‘Glycolysis/Gluconeogenesis’ (164, 3.1%) (Fig. 4A, Supplementary Table S3). The DEGs in leaves were mainly enriched in ‘Carbon fixation in photosynthesis’ (116, 3.1%), ‘Cysteine and methionine metabolism’ (100, 2.6%) and ‘Glycolysis/Gluconeogenesis’ (114, 3%) (Fig. 4B, Supplementary Table S4).

### Addition of 3-MA affects the transcriptomic responses of wheat roots and leaves to salt stress

In this study, 121 and 139 DEGs were detected in the wheat roots and leaves with 3-MA addition under salt stress treatment (*P* < 0.001 and |log2FoldChange| > 7) (Table 5 and Table 6). The expression of 115 genes, inducing 18 peroxidase genes, beta-glucosidase 5-like isoform X1, chalcone synthase genes, ect.in wheat roots was decreased significantly by 3-MA addition under salt stress. Therefore, the 3-MA-induced downregulation of these genes may play important roles in decreasing wheat root tolerance to salt stress. The expression of 25 genes, inducing ethylene-responsive transcription factor, resistance proteins, MYB, ect.in wheat leaves was also decreased significantly by 3-MA addition under salt stress. Four calmodulin-like genes were upregulated.

**Table 5:** The DEGs were detected in the wheat roots with 3-MA added to the salt stress treatment (*p*-value < 0.001 and |log2FoldChange| > 7)

Gene_id	TGvsCG_log2FoldChange	TGvsCG_*p*value	TMGvsTG_log2FoldChange	TMGvsTG_*p*value	Gene_strand	Gene_length	Gene_biotype	nr annotation
TraesCS3B02G483500	0.1	0.8544	−7.8	3.49E-10	+	2416	protein_coding	beta-glucosidase 5-like isoform X1 [*Aegilops tauschii* subsp. tauschii]
TraesCS2A02G527700	0.6	0.15539	−8.3	1.04E-11	+	1724	protein_coding	chalcone synthase [Oryza officinalis]
TraesCS7D02G469600	−1.3	0.05025	−7.2	9.22E-19	–	402	protein_coding	cortical cell-delineating protein-like [*Aegilops tauschii* subsp. tauschii]
TraesCS5A02G548600	1.6	#######	−7.3	#######	–	1649	protein_coding	predicted protein, partial [*Hordeum vulgare* subsp. vulgare]
TraesCS2D02G300100	1.4	0.00225	−9.9	1.10E-21	+	1272	protein_coding	loricrin-like [*Aegilops tauschii* subsp. tauschii]
TraesCS2A02G411600	−1.6	0.07519	−7.4	1.27E-08	–	381	protein_coding	protease inhibitor-like protein [*Triticum aestivum*]
TraesCS4D02G360000	1.5	0.002	−8.7	2.21E-12	+	426	protein_coding	pEARLI1-like lipid transfer protein 1 [*Aegilops tauschii* subsp. tauschii]
TraesCS2B02G126300	−1.4	0.14191	−8	3.46E-05	–	1254	protein_coding	peroxidase 70 [*Oryza sativa* Japonica Group]
TraesCS1D02G331800	−1.6	0.08427	−7.9	1.29E-05	–	351	protein_coding	cortical cell-delineating protein-like [*Aegilops tauschii* subsp. tauschii]
TraesCS1D02G331900	−1.2	0.18531	−9.9	4.33E-09	–	441	protein_coding	cortical cell-delineating protein-like [*Aegilops tauschii* subsp. tauschii]
TraesCS7B02G317800	−2.4	#######	−7.2	4.86E-18	+	384	protein_coding	lipid transfer protein EARLI 1-like [*Aegilops tauschii* subsp. tauschii]
TraesCS7A02G417700	−4.4	#######	−8.2	2.90E-10	+	384	protein_coding	14 kDa proline-rich protein DC2.15 [*Triticum urartu*]
TraesCS1B02G135800	−3.7	0.00452	8.44	NA	–	719	protein_coding	metallothioneine type1, partial [*Hordeum vulgare* subsp. vulgare]
TraesCS7B02G298400	−2.5	0.00327	−9.6	4.47E-08	+	1266	protein_coding	peroxidase 2-like [*Aegilops tauschii* subsp. tauschii]
TraesCS2A02G301500	−0.8	0.17673	−8.1	3.16E-10	+	1132	protein_coding	loricrin-like [*Aegilops tauschii* subsp. tauschii]
TraesCS1D02G213400	−1.2	0.18376	−9.6	2.94E-09	+	345	protein_coding	cortical cell-delineating protein-like [*Aegilops tauschii* subsp. tauschii]
TraesCS2B02G053200	6.4	#######	−9.2	1.78E-09	+	1132	protein_coding	xylanase inhibitor protein 1-like [*Aegilops tauschii* subsp. tauschii]
TraesCS1D02G285900	−1.7	0.00192	−7.2	5.92E-07	+	828	protein_coding	uncharacterized protein LOC109748351 [*Aegilops tauschii* subsp. tauschii]
TraesCS7B02G384900	−1.6	0.19774	−8.2	4.81E-05	–	399	protein_coding	predicted protein [*Hordeum vulgare* subsp. vulgare]
TraesCS3D02G538500	0.4	0.30155	−7.2	9.03E-09	–	1422	protein_coding	pectin acetylesterase 5-like isoform X1 [*Aegilops tauschii* subsp. tauschii]
TraesCS7D02G464600	−1	0.00271	−8	2.11E-18	–	1352	protein_coding	predicted protein [*Hordeum vulgare* subsp. vulgare]
TraesCS1D02G027300	−0.9	0.01898	−7.7	1.62E-19	+	300	protein_coding	Subtilisin-chymotrypsin inhibitor-2A [*Triticum urartu*]
TraesCS4D02G310500	0.3	0.76204	−7.8	5.71E-10	+	480	protein_coding	cortical cell-delineating protein-like [*Aegilops tauschii* subsp. tauschii]
TraesCS7A02G477100	−1.5	0.00078	−10	4.68E-15	–	1353	protein_coding	peroxidase 70-like [*Aegilops tauschii* subsp. tauschii]
TraesCS7A02G452900	0	0.9174	−7.3	2.93E-30	+	1299	protein_coding	peroxidase 39-like [*Aegilops tauschii* subsp. tauschii]
TraesCS7B02G384600	−3.6	0.00019	−8.2	3.60E-08	–	399	protein_coding	predicted protein [*Hordeum vulgare* subsp. vulgare]
TraesCS7A02G396400	−2	0.01087	−7.5	2.70E-10	+	1247	protein_coding	peroxidase 2-like [*Aegilops tauschii* subsp. tauschii]
TraesCS1D02G027400	1.4	0.00631	−7.6	3.71E−11	+	237	protein_coding	Subtilisin-chymotrypsin inhibitor-2A [*Triticum urartu*]
TraesCS3D02G467700	2.1	#######	-11	4.96E-14	+	1307	protein_coding	basic 7S globulin-like [*Aegilops tauschii* subsp. tauschii]
TraesCS7B02G273700	−1.7	0.02873	−7.6	2.35E-10	+	1563	protein_coding	berberine bridge enzyme-like 27 [*Aegilops tauschii* subsp. tauschii]
novel.8984	NA	NA	8.73	4.41E-12	+	401	–	–
TraesCS1B02G330200	−1.7	0.00063	−8.2	4.90E-10	–	1441	protein_coding	peroxidase 1-like [*Aegilops tauschii* subsp. tauschii]
TraesCS1B02G331200	−2.2	0.01578	−7.9	1.68E-08	+	1286	protein_coding	peroxidase 1-like [*Aegilops tauschii* subsp. tauschii]
TraesCS7B02G298600	−2.3	#######	−7.7	6.86E-09	+	1313	protein_coding	peroxidase 2-like [*Aegilops tauschii* subsp. tauschii]
TraesCS1D02G318000	−2.3	#######	−8.6	2.81E-11	+	1419	protein_coding	peroxidase 1-like [*Aegilops tauschii* subsp. tauschii]
TraesCS2A02G267200	−1.7	#######	−7.2	5.42E-11	–	548	protein_coding	hypothetical protein TRIUR3_15154 [*Triticum urartu*]
TraesCS1A02G329700	−0.6	0.59623	−9.7	2.03E-05	–	351	protein_coding	Cortical cell-delineating protein [*Triticum urartu*]
TraesCS2B02G482300	−0.9	0.56442	−7.2	0.0127	+	324	protein_coding	RecName: Full = Cold-regulated protein 2
TraesCS2D02G107800	8.4	#######	−9.7	6.98E-06	–	1256	protein_coding	peroxidase 1-like [*Aegilops tauschii* subsp. tauschii]
TraesCS7D02G469425	−1	0.0067	−9.4	1.03E-13	–	324	protein_coding	cortical cell-delineating protein-like [*Aegilops tauschii* subsp. tauschii]
TraesCSU02G047800	1.1	#######	−10	1.30E-17	–	2025	protein_coding	unnamed protein product [*Triticum aestivum*]
TraesCS6B02G280400	−1.8	0.00011	−7.4	2.21E-08	+	2675	protein_coding	beta-D-xylosidase 3-like [*Aegilops tauschii* subsp. tauschii]
TraesCS2D02G108100	2.4	#######	−10	5.40E-11	–	1272	protein_coding	unnamed protein product [*Triticum aestivum*]
novel.13266	−2.3	#######	−8.3	4.98E-11	–	3879	–	uncharacterized protein LOC109771772 [*Aegilops tauschii* subsp. tauschii]
TraesCS2A02G040500	8.3	#######	−9.6	2.83E-13	–	906	protein_coding	xylanase inhibitor protein 1-like [*Aegilops tauschii* subsp. tauschii]
TraesCS1B02G330500	−2.2	#######	−8.3	9.69E-10	+	1425	protein_coding	peroxidase 1-like [*Aegilops tauschii* subsp. tauschii]
TraesCS3D02G318000	−2	#######	−8.3	2.41E-10	–	1296	protein_coding	blue copper protein-like [*Aegilops tauschii* subsp. tauschii]
TraesCSU02G144300	4.5	0.0004	−7.1	9.90E-05	+	390	protein_coding	cortical cell-delineating protein-like [*Aegilops tauschii* subsp. tauschii]
TraesCSU02G144600	2.8	#######	−10	1.29E-15	+	465	protein_coding	cortical cell-delineating protein-like [*Aegilops tauschii* subsp. tauschii]
TraesCS4A02G412900	3.6	0.01668	−8.4	9.08E-05	+	426	protein_coding	Cortical cell-delineating protein [*Triticum urartu*]
TraesCSU02G144500	1.2	0.05018	−7.9	5.33E-11	+	432	protein_coding	cortical cell-delineating protein-like [*Aegilops tauschii* subsp. tauschii]
TraesCS7D02G049400	1.3	#######	−9.8	8.55E-16	–	1937	protein_coding	protein NRT1/ PTR FAMILY 2.3-like [*Aegilops tauschii* subsp. tauschii]
TraesCS1A02G356100	−1.5	0.00349	−7.5	8.00E-09	+	1194	protein_coding	peroxidase 1-like [*Aegilops tauschii* subsp. tauschii]
TraesCS4B02G368300	2.6	#######	−9.9	1.31E-14	–	432	protein_coding	predicted protein [*Hordeum vulgare* subsp. vulgare]
TraesCS7B02G298500	− 1.4	0.16193	−8.3	8.13E-09	+	1221	protein_coding	peroxidase 2-like [*Aegilops tauschii* subsp. tauschii]
TraesCS2D02G008100	−1.3	0.01092	−8.4	5.01E-10	+	1794	protein_coding	unnamed protein product [*Triticum aestivum*]
TraesCS2D02G108300	−1.4	0.00055	−7.4	9.36E-09	–	1291	protein_coding	unnamed protein product [*Triticum aestivum*]
TraesCS5A02G535500	3	#######	−9.4	9.50E-13	–	507	protein_coding	Cortical cell-delineating protein [*Triticum urartu*]
TraesCS7B02G010600	−1.9	#######	−8	2.78E-09	+	258	protein_coding	uncharacterized protein LOC109785281 [*Aegilops tauschii* subsp. tauschii]
TraesCS2D02G039300	7.7	#######	−7.1	5.07E-08	+	1201	protein_coding	xylanase inhibitor protein 1-like [*Aegilops tauschii* subsp. tauschii]
TraesCS1A02G318000	−2.9	#######	−7.1	3.60E-06	–	1300	protein_coding	peroxidase 1-like [*Aegilops tauschii* subsp. tauschii]
TraesCSU02G144800	5.8	#######	−9.4	4.04E-13	+	519	protein_coding	pEARLI1-like lipid transfer protein 2 [*Aegilops tauschii* subsp. tauschii]
TraesCS3A02G077100	NA	NA	7.41	4.33E-09	+	2245	protein_coding	asparagine synthetase [*Triticum turgidum* subsp. durum]
TraesCS1A02G318200	−2.8	#######	−7.1	1.20E-07	–	1241	protein_coding	peroxidase 1-like [*Aegilops tauschii* subsp. tauschii]
TraesCS4A02G409400	−1.5	0.01447	−7.1	5.76E-07	–	3492	protein_coding	MDIS1-interacting receptor like kinase 1-like [*Aegilops tauschii* subsp. tauschii]
TraesCSU02G243200	−2.7	#######	−7.1	8.95E-08	+	754	protein_coding	unnamed protein product [*Triticum aestivum*]
TraesCS7D02G539100	−1.7	0.00013	−8	3.00E-09	+	1344	protein_coding	tricetin 3′,4′,5′-O-trimethyltransferase-like [*Aegilops tauschii* subsp. tauschii]
TraesCS4A02G339000	1.6	#######	−9.3	3.20E-14	–	1876	protein_coding	4-hydroxyphenylacetaldehyde oxime monooxygenase-like [*Aegilops tauschii* subsp. tauschii]
TraesCS4B02G369000	4.5	0.00018	−9.7	1.14E-09	–	390	protein_coding	cortical cell-delineating protein-like [*Aegilops tauschii* subsp. tauschii]
TraesCS5A02G418600	−0.4	0.56518	−8.7	6.85E-06	+	609	protein_coding	predicted protein [*Hordeum vulgare* subsp. vulgare]
TraesCS2D02G458700	−4.4	0.1389	23.6	NA	–	651	protein_coding	hypothetical protein OsI_31610 [*Oryza sativa* Indica Group]
TraesCS1A02G209900	−0.4	0.36364	−8.7	1.23E-11	–	339	protein_coding	cortical cell-delineating protein-like [*Aegilops tauschii* subsp. tauschii]
TraesCS1D02G318600	−2.7	#######	−7.1	1.34E-06	+	1274	protein_coding	peroxidase 1-like [*Aegilops tauschii* subsp. tauschii]
TraesCS3A02G324700	−1.4	0.01242	−8	1.52E-08	–	894	protein_coding	unnamed protein product [*Triticum aestivum*]
TraesCS1B02G027800	0.2	0.68119	−7	2.04E-09	–	616	protein_coding	Bowman-Birk trypsin inhibitor-like protein [*Triticum aestivum*]
TraesCS7A02G054100	0.4	0.23441	−9	2.89E-13	–	1974	protein_coding	protein NRT1/ PTR FAMILY 2.3-like [*Aegilops tauschii* subsp. tauschii]
TraesCSU02G243695	4.8	0.00051	−7.7	2.25E-06	–	347	protein_coding	cortical cell-delineating protein-like [*Aegilops tauschii* subsp. tauschii]
novel.2861	NA	NA	23.5	NA	+	1363	–	–
TraesCS2A02G002900	2.8	#######	−8.8	2.01E−12	–	1911	protein_coding	Cytochrome P450 86B1 [*Triticum urartu*]
TraesCS2A02G108000	-1	0.11668	−8.2	2.21E-08	–	1261	protein_coding	unnamed protein product [*Triticum aestivum*]
TraesCS4B02G313200	1.1	0.026	−8.1	1.13E-10	+	480	protein_coding	cortical cell-delineating protein-like [*Aegilops tauschii* subsp. tauschii]
TraesCS1A02G129600	4.2	0.00012	−7.1	3.97E-07	+	905	protein_coding	bidirectional sugar transporter SWEET3a [*Aegilops tauschii* subsp. tauschii]
novel.1280	−2.4	#######	−7.2	8.06E-07	–	2191	–	extensin-like [*Aegilops tauschii* subsp. tauschii]
TraesCS5D02G561700	0.7	0.1221	−7.9	5.31E-10	+	1302	protein_coding	aquaporin PIP2–2-like [*Aegilops tauschii* subsp. tauschii]
TraesCS7D02G410700	−1.7	0.0053	−7.5	2.99E-07	+	384	protein_coding	lipid transfer protein EARLI 1-like [*Aegilops tauschii* subsp. tauschii]
TraesCS6B02G018700	−0.3	0.43244	−8.3	6.09E-11	–	1220	protein_coding	extradiol ring-cleavage dioxygenase-like [*Aegilops tauschii* subsp. tauschii]
TraesCS2A02G108200	−1.5	#######	−7.6	6.68E-09	–	1328	protein_coding	unnamed protein product [*Triticum aestivum*]
TraesCS4D02G208900	4.3	#######	−7.1	9.72E-08	+	2126	protein_coding	laccase-10-like [*Aegilops tauschii* subsp. tauschii]
novel.13303	−1.8	0.0179	−7.3	6.61E-06	–	754	–	–
TraesCS6A02G137200	−2	0.00034	−7.1	6.16E-07	+	845	protein_coding	Early nodulin-like protein 2 [*Triticum urartu*]
TraesCS2B02G173200	−1.8	0.00115	−7.2	4.05E-07	+	1372	protein_coding	peroxidase 2-like [*Aegilops tauschii* subsp. tauschii]
TraesCSU02G242200	3.6	#######	−8	1.60E-09	+	390	protein_coding	cortical cell-delineating protein-like [*Aegilops tauschii* subsp. tauschii]
TraesCS1D02G021900	2.3	NA	−8.8	NA	–	378	protein_coding	Bowman-Birk trypsin inhibitor-like protein [*Triticum aestivum*]
TraesCS1D02G050600	2.3	0.01591	−8.8	6.75E-05	+	709	protein_coding	Salt stress-induced protein [*Dichanthelium oligosanthes*]
TraesCS3A02G460900	−1.9	0.00107	−7	1.21E−06	–	1708	protein_coding	probable leucine-rich repeat receptor-like protein kinase At5g49770 [*Aegilops tauschii* subsp. tauschii]
TraesCS7B02G002000	-0	0.94185	−8.1	2.61E-10	–	855	protein_coding	aquaporin PIP2–2-like [*Aegilops tauschii* subsp. tauschii]
novel.890	NA	NA	22.9	NA	+	1511	–	hypothetical protein TRIUR3_19550 [*Triticum urartu*]
TraesCS1D02G214500	4	0.04098	−8.8	0.00081	+	672	protein_coding	predicted protein [*Hordeum vulgare* subsp. vulgare]
TraesCS4D02G104000	5.2	#######	−8.8	3.83E-12	+	528	protein_coding	dirigent protein 21-like [*Aegilops tauschii* subsp. tauschii]
TraesCS5D02G004100	0	0.98706	−7.1	4.42E-08	+	447	protein_coding	puroindoline a [*Triticum turgidum* subsp. durum x *Aegilops tauschii*]
TraesCS7D02G020100	6	0.00022	−7.4	0.00281	–	1173	protein_coding	extensin-like isoform X1 [*Aegilops tauschii* subsp. tauschii]
TraesCS1D02G006600	3.6	#######	−8.7	2.09E-11	+	990	protein_coding	hypothetical protein TRIUR3_28534 [*Triticum urartu*]
TraesCS7B02G115300	4.8	#######	−7	9.21E-08	–	674	protein_coding	GASR7 [*Triticum aestivum*]
TraesCS2D02G152700	−0.2	0.77316	−7.8	9.40E-09	–	1540	protein_coding	peroxidase 2-like [*Aegilops tauschii* subsp. tauschii]
TraesCS5B02G528700	−0.4	0.47088	−7.6	3.86E-08	+	1187	protein_coding	expansin-A31-like [*Aegilops tauschii* subsp. tauschii]
TraesCS5A02G558000	−0.4	0.33336	−7.6	6.13E-09	–	1616	protein_coding	probable receptor-like protein kinase At4g10390 [*Aegilops tauschii* subsp. tauschii]
TraesCSU02G154100	5.5	0.00709	−8.6	0.0002	+	390	protein_coding	cortical cell-delineating protein-like [*Aegilops tauschii* subsp. tauschii]
TraesCS4B02G367800	1.8	0.07225	−8.2	1.50E-08	–	432	protein_coding	cortical cell-delineating protein-like [*Aegilops tauschii* subsp. tauschii]
novel.3884	−0.8	0.06377	−7.3	7.32E-08	+	576	–	predicted protein [*Hordeum vulgare* subsp. vulgare]
TraesCS7D02G139500	0.9	0.17508	−7.7	1.47E-08	+	1554	protein_coding	uncharacterized protein LOC109732600 [*Aegilops tauschii* subsp. tauschii]
TraesCS3B02G405900	−0.2	0.79462	−7.1	1.10E-06	+	1153	protein_coding	unnamed protein product [*Triticum aestivum*]
novel.4897	2.4	0.37495	−7.7	0.04761	+	1614	–	predicted protein [*Hordeum vulgare* subsp. vulgare]
TraesCS2A02G002800	4.4	#######	−7.7	3.85E-09	+	1978	protein_coding	Cytochrome P450 86B1 [*Triticum urartu*]
TraesCS5D02G249900	1.3	0.1679	−7.4	2.04E-06	+	300	protein_coding	hypothetical protein TRIUR3_16342 [*Triticum urartu*]
TraesCS3D02G338600	0.9	0.16034	−7.3	2.80E-07	+	1292	protein_coding	expansin-A2 [*Oryza sativa* Japonica Group]
TraesCS5A02G243400	1.4	0.05229	−7.4	6.90E-08	+	288	protein_coding	hypothetical protein TRIUR3_16342 [*Triticum urartu*]
TraesCS1D02G050800	3.9	0.00035	−7.6	7.24E-08	+	792	protein_coding	LOW QUALITY PROTEIN: mannose/glucose-specific lectin [*Setaria italica*]
TraesCS1D02G050700	4	0.00047	−7.5	2.65E-07	+	486	protein_coding	LOW QUALITY PROTEIN: mannose/glucose-specific lectin [*Setaria italica*]
TraesCS7A02G452400	1.4	0.05297	−7.2	1.23E-07	+	1652	protein_coding	probable glycosyltransferase 4 [*Aegilops tauschii* subsp. tauschii]
TraesCSU02G185500	6.1	#######	−7.4	1.04E-07	+	390	protein_coding	cortical cell-delineating protein-like [*Aegilops tauschii* subsp. tauschii]
TraesCS5A02G270300	7	#######	−7.4	6.05E-08	+	2445	protein_coding	G-type lectin S-receptor-like serine/threonine-protein kinase [*Panicum miliaceum*]

**Table 6:** The DEGs were detected in the wheat leaves with 3-MA added to the salt stress treatment (*p*-value < 0.001 and |log2FoldChange| > 7)

Gene_id	TGvsCG_log2FoldChange	TGvsCG_*p*value	TMGvsTG_log2FoldChange	TMGvsTG_*p*value	Gene_strand	Gene_length	Gene_biotype	nr annotation
TraesCS4D02G238600	−0.3323801	0.7931235	8.1020348	6.324E-16	+	765	protein_coding	hemoglobin 1 [*Triticum aestivum*]
TraesCS7B02G105300	4.214515	0.0523772	8.7327373	2.277E-81	+	777	protein_coding	pathogenesis-related protein 1–17 [*Triticum aestivum*]
TraesCS4B02G237300	−2.1334831	0.5180465	9.6167847	1.334E-06	+	774	protein_coding	Non-symbiotic hemoglobin [*Triticum urartu*]
TraesCS1D02G266000	1.5552708	0.4394289	7.0341381	4.737E-09	+	744	protein_coding	uncharacterized protein LOC109755131 [*Aegilops tauschii* subsp. tauschii]
TraesCS2D02G431500	−0.4780261	0.8477712	8.2992476	1.236E-07	+	1281	protein_coding	unnamed protein product [*Triticum aestivum*]
TraesCS5D02G507800	−2.7621685	0.031361	7.8176373	9.738E-10	–	853	protein_coding	cell number regulator 10-like [*Aegilops tauschii* subsp. tauschii]
TraesCS7B02G253600	−1.2392657	0.6291003	11.204843	6.619E-07	–	1735	protein_coding	probable gamma-aminobutyrate transaminase 4 [*Oryza sativa* Japonica Group]
TraesCS4D02G200700	−0.7818844	0.4933324	8.4153668	5.429E-08	+	1104	protein_coding	protein IN2–1-like isoform X2 [*Aegilops tauschii* subsp. tauschii]
TraesCS5B02G128100	−3.6421817	7.23E-23	−8.3302015	1.231E-10	+	1527	protein_coding	cytochrome P450 94C1-like [*Aegilops tauschii* subsp. tauschii]
TraesCS3B02G328700	−3.0000069	0.0006569	7.479858	3.09E-08	–	1640	protein_coding	unnamed protein product [*Triticum aestivum*]
TraesCS7B02G107700	−1.5379386	8.541E-05	−7.0838361	2.372E-12	+	817	protein_coding	protein TIFY 11e-like [*Aegilops tauschii* subsp. tauschii]
TraesCS4A02G061700	−3.6104814	0.2473155	11.105673	4.846E-08	+	824	protein_coding	hemoglobin 1 [*Triticum aestivum*]
TraesCS7B02G107800	−1.5677406	0.0290698	−7.3593804	4.586E-09	+	690	protein_coding	Protein TIFY 3A [*Triticum urartu*]
TraesCS1A02G303100	1.394171	0.729974	8.9475077	7.043E-07	+	2234	protein_coding	cytochrome P450 714D1-like [*Aegilops tauschii* subsp. tauschii]
TraesCS1B02G389700	−2.2210215	0.0537236	−7.7456662	1.147E-06	+	1007	protein_coding	ethylene responsive transcription factor 6 [*Triticum turgidum* subsp. durum]
TraesCS7B02G245000	1.0239382	0.7514332	7.4810508	6.323E-13	–	192	protein_coding	hypothetical protein TRIUR3_01539 [*Triticum urartu*]
TraesCS4B02G199700	−3.2189175	4.882E-05	9.3039195	5.731E-65	+	1047	protein_coding	protein IN2–1-like isoform X2 [*Aegilops tauschii* subsp. tauschii]
TraesCS1A02G370600	−2.4884592	0.0195036	−8.2137194	4.94E-07	+	1257	protein_coding	AP2 domain containing protein [*Zea mays*]
TraesCS7B02G108000	−1.3915841	0.0008718	−7.4844356	9.126E-10	+	675	protein_coding	predicted protein [*Hordeum vulgare* subsp. vulgare]
TraesCS4A02G104300	−2.6154062	0.0023405	8.5615901	3.856E-23	–	1226	protein_coding	Protein IN2–1 [*Triticum urartu*]
novel.3130	0.8170057	0.3808812	7.9498956	4.758E-24	–	7163	–	OSJNBb0034I13.10 [*Oryza sativa* Japonica Group]
TraesCS1A02G186400	−2.2807992	0.0120042	8.3724605	4.069E-22	+	1025	protein_coding	putative glutathione S-transferase GSTU6 [*Triticum urartu*]
TraesCS3A02G212300	−2.3643168	0.5576773	8.4079095	2.968E-09	+	1982	protein_coding	PREDICTED: aldehyde dehydrogenase family 2 member C4-like [Oryza brachyantha]
TraesCS3D02G049700	−3.8165125	0.2879584	8.371108	2.336E-09	+	1998	protein_coding	LEAF RUST 10 DISEASE-RESISTANCE LOCUS RECEPTOR-LIKE PROTEIN KINASE-like 2.4 [*Aegilops tauschii* subsp. tauschii]
TraesCS6A02G297500	−2.3842856	0.3196855	7.3373451	3.262E-10	–	884	protein_coding	blue copper protein-like [*Aegilops tauschii* subsp. tauschii]
TraesCS7B02G108300	−2.6183375	4.549E-15	−7.041706	6.78E-17	+	821	protein_coding	protein TIFY 11e-like [*Aegilops tauschii* subsp. tauschii]
TraesCS7B02G150000	−0.7197533	0.24143	−7.2565587	4.506E-07	+	1309	protein_coding	RING-H2 finger protein ATL16-like [*Aegilops tauschii* subsp. tauschii]
TraesCS5B02G148300	−0.0055847	0.9980956	10.150592	4.15E-18	+	1664	protein_coding	UDP-glycosyltransferase [*Triticum aestivum*]
TraesCS5A02G149600	0.4517151	0.9109179	10.87046	3.259E-15	+	2318	protein_coding	UDP-glycosyltransferase 74E2-like [*Aegilops tauschii* subsp. tauschii]
TraesCS6D02G302300	−0.87443	0.285645	8.0376595	5.41E-55	–	1508	protein_coding	RecName: Full = Putative 12-oxophytodienoate reductase 11; AltName: Full = OPDA-reductase 11; Short = OsOPR11
TraesCS2B02G454500	1.9783823	0.6205002	10.786639	4.309E-13	+	1499	protein_coding	putative xyloglucan endotransglucosylase/hydrolase protein 13 [*Aegilops tauschii* subsp. tauschii]
TraesCS7D02G204700	−2.8301953	0.0003421	−7.1062002	8.97E-09	+	742	protein_coding	protein TIFY 11e-like [*Aegilops tauschii* subsp. tauschii]
TraesCS5D02G360600	2.3930151	0.5470208	10.597521	1.688E-11	–	1005	protein_coding	11S globulin seed storage protein 2-like [*Aegilops tauschii* subsp. tauschii]
TraesCS6A02G181000	1.0236555	0.5799038	9.2447277	4.658E-12	+	1652	protein_coding	Premnaspirodiene oxygenase [*Triticum urartu*]
TraesCS5A02G017900	−1.3019367	0.5125593	7.101288	0.000169	–	942	protein_coding	thaumatin-like protein [*Triticum aestivum*]
novel.9925	−3.4360585	0.095649	8.1593202	8.451E-11	–	1002	–	12-oxophytodienoic acid reductase 2 [*Hordeum vulgare* subsp. vulgare]
TraesCS4B02G086100	−0.7953882	0.6826519	9.1847692	5.271E-07	+	1355	protein_coding	2-alkenal reductase (NADP(+)-dependent)-like [*Aegilops tauschii* subsp. tauschii]
TraesCS3D02G514300	−0.4473872	0.9126941	12.413653	8.099E-17	–	1447	protein_coding	3-oxo-Delta(4,5)-steroid 5-beta-reductase-like [*Aegilops tauschii* subsp. tauschii]
novel.5891	1.9985882	0.6168275	8.7138732	1.386E-20	–	2166	–	glutathione transferase [*Triticum aestivum*]
novel.12621	−1.8906479	0.0266958	−7.0792994	2.871E-07	–	4559	–	alpha-terpineol synthase, chloroplastic-like [*Aegilops tauschii* subsp. tauschii]
TraesCS3D02G104400	−1.9016134	0.0823456	7.6956478	2.662E-15	–	1160	protein_coding	probable NADPH:quinone oxidoreductase 1 [*Aegilops tauschii* subsp. tauschii]
TraesCS1A02G370400	−2.5463721	0.0673874	−8.22026	0.0019948	+	1341	protein_coding	ethylene-responsive transcription factor ERF109-like [*Aegilops tauschii* subsp. tauschii]
TraesCS3D02G514400	−2.6997891	0.0006367	7.245715	4.114E-07	+	1420	protein_coding	3-oxo-Delta(4,5)-steroid 5-beta-reductase-like [*Aegilops tauschii* subsp. tauschii]
TraesCS5A02G238400	−1.6455602	0.3716241	−7.373527	0.0162525	+	1179	protein_coding	ethylene-responsive transcription factor ERF109-like [*Aegilops tauschii* subsp. tauschii]
TraesCS1D02G190200	−0.6747421	0.5327128	8.46699	6.26E-43	–	997	protein_coding	glutathione S-transferase U17-like [*Aegilops tauschii* subsp. tauschii]
TraesCS3D02G483000	3.4059669	0.2493825	9.0171295	1.424E-15	–	1273	protein_coding	glutathione S-transferase U8-like [*Aegilops tauschii* subsp. tauschii]
TraesCS6D02G127700	−3.9439956	0.0009776	9.6526212	7.343E-23	–	1102	protein_coding	glutathione S-transferase 1 [*Aegilops tauschii*]
TraesCS2D02G030500	−6.3413476	0.0109846	−7.0637497	0.0708707	–	1399	protein_coding	unnamed protein product [*Triticum aestivum*]
TraesCS7D02G466900	1.9783823	0.6205002	8.8562647	6.602E-23	+	1465	protein_coding	ureide permease 1-like [*Aegilops tauschii* subsp. tauschii]
TraesCS6D02G127800	0.2498907	0.9138951	7.9662513	3.474E-05	–	1017	protein_coding	glutathione-S-transferase 2 [*Aegilops tauschii*]
TraesCS7B02G264200	−4.3495971	0.1855559	7.6096234	7.696E-15	–	2736	protein_coding	beta-galactosidase precursor [*Zea mays*]
TraesCS3B02G290200	−4.2850591	0.1092021	12.755727	8.125E-16	–	1737	protein_coding	unnamed protein product [*Triticum aestivum*]
TraesCS7B02G290200	0.43239	0.9147506	9.0797725	1.443E-15	–	707	protein_coding	60S acidic ribosomal protein P2A-like [*Aegilops tauschii* subsp. tauschii]
TraesCS6A02G260900	−0.3766796	0.9072237	8.6940862	3.771E-08	–	1495	protein_coding	Anthranilate N-benzoyltransferase protein 1 [*Triticum urartu*]
TraesCS1D02G073000	−0.4473872	0.9126941	8.5796305	1.73E-10	+	1748	protein_coding	nicotianamine aminotransferase A-like [*Aegilops tauschii* subsp. tauschii]
TraesCS2D02G442700	−4.9781944	0.0002759	8.2422757	0.0005716	–	2160	protein_coding	protein NRT1/ PTR FAMILY 8.3-like [*Aegilops tauschii* subsp. tauschii]
TraesCS3B02G220700	2.0384366	0.461114	7.873967	1.185E-09	–	864	protein_coding	eukaryotic translation initiation factor 6–2 [Arachis duranensis]
TraesCS7B02G400500	−5.2197164	0.005458	12.06558	3.747E-14	–	1716	protein_coding	protein DETOXIFICATION 16-like isoform X2 [*Aegilops tauschii* subsp. tauschii]
TraesCS6B02G011500	−3.1745648	0.1952765	9.1026764	9.984E-14	–	905	protein_coding	thiosulfate sulfurtransferase 16, chloroplastic-like [*Aegilops tauschii* subsp. tauschii]
TraesCS6B02G052200	−4.4009609	0.1650036	10.092898	1.495E-07	+	1781	protein_coding	indole-2-monooxygenase-like [*Aegilops tauschii* subsp. tauschii]
TraesCS7D02G466800	2.4009292	0.527902	8.7596636	2.897E-30	–	1619	protein_coding	ureide permease 1-like [*Aegilops tauschii* subsp. tauschii]
TraesCS6A02G139100	0.1600182	0.9478011	7.9627226	7.446E-05	–	1083	protein_coding	glutathione-S-transferase 2 [*Aegilops tauschii*]
TraesCS2D02G582000	−4.9309655	0.0858604	10.780386	5.116E-06	–	1650	protein_coding	tryptamine benzoyltransferase 1-like [*Aegilops tauschii* subsp. tauschii]
TraesCS6A02G331300	−1.088292	0.6040891	8.4161256	1.231E-10	+	1765	protein_coding	7-deoxyloganetin glucosyltransferase-like [*Aegilops tauschii* subsp. tauschii]
TraesCS2D02G072600	2.0047293	0.3191813	7.043509	2.799E-25	+	1807	protein_coding	UDP-glycosyltransferase 74F2-like [*Aegilops tauschii* subsp. tauschii]
TraesCS3A02G260100	−2.7183185	0.0996397	7.6304841	5.634E-06	–	1256	protein_coding	endochitinase [*Triticum aestivum*]
TraesCS2A02G534600	2.71469	0.4930462	8.5851595	1.544E-06	+	1837	protein_coding	isoflavone 2′-hydroxylase-like [*Aegilops tauschii* subsp. tauschii]
novel.9926	−2.2967107	0.0958291	7.1765368	4.322E-06	–	384	–	putative 12-oxophytodienoate reductase 11 [*Aegilops tauschii* subsp. tauschii]
TraesCS7B02G131600	−3.721248	2.984E-40	−8.8556802	2.034E-12	+	243	protein_coding	MYB-related protein [*Aegilops speltoides*]
TraesCS6B02G167600	−3.6208092	0.2525152	11.2974	1.648E-09	–	917	protein_coding	probable glutathione S-transferase GSTU6 [*Aegilops tauschii* subsp. tauschii]
TraesCS4B02G188800	−5.1887629	0.0069538	7.8397786	8.053E-08	–	1208	protein_coding	sex determination protein tasselseed-2-like [*Aegilops tauschii* subsp. tauschii]
TraesCS3B02G536100	−1.4279693	0.4922008	8.7614661	2.1E-08	–	972	protein_coding	glutathione S-transferase U8-like [*Aegilops tauschii* subsp. tauschii]
TraesCS7D02G328700	−4.1326269	0.0839718	7.835076	2.291E-07	+	878	protein_coding	uncharacterized protein LOC109748612 [*Aegilops tauschii* subsp. tauschii]
TraesCS4A02G454800	−4.7428261	0.0515433	11.301311	4.134E-19	+	1060	protein_coding	probable glutathione S-transferase GSTU6 [*Aegilops tauschii* subsp. tauschii]
novel.12163	1.935393	0.4021517	7.5227918	4.243E-09	+	2047	–	hypothetical protein TRIUR3_06504 [*Triticum urartu*]
TraesCS1D02G338500	−1.4019942	0.7201248	9.2965547	1.6E-08	+	933	protein_coding	28 kDa heat- and acid-stable phosphoprotein-like [*Aegilops tauschii* subsp. tauschii]
TraesCS1A02G435100	1.0156952	0.6669168	7.1021758	7.996E-07	+	792	protein_coding	Calmodulin-like protein 5 [*Triticum urartu*]
TraesCS5D02G312100	0.1393233	0.9422517	8.1572609	5.841E-14	+	1645	protein_coding	UDP-glycosyltransferase 74E1-like [*Aegilops tauschii* subsp. tauschii]
TraesCS7B02G105100	−0.9610666	0.7773397	8.4600953	2.534E-14	+	866	protein_coding	pathogenesis-related protein 1–18 [*Triticum aestivum*]
TraesCS5B02G267800	0.0477745	0.9877232	8.421405	1.832E-08	+	1557	protein_coding	protein PIN-LIKES 3-like [*Aegilops tauschii* subsp. tauschii]
novel.13733	−1.0982927	0.4496533	7.4881966	1.21E-11	–	525	–	–
TraesCS2B02G187200	−2.2373153	0.5800992	7.6214849	4.772E-07	–	1382	protein_coding	probable trehalose-phosphate phosphatase 3 [*Aegilops tauschii* subsp. tauschii]
TraesCS7D02G168600	−0.7021325	0.6529766	7.4453677	5.308E-20	–	882	protein_coding	cyclic phosphodiesterase-like [*Aegilops tauschii* subsp. tauschii]
TraesCS6D02G332900	2.7462839	0.4879009	7.402371	1.27E-06	+	1100	protein_coding	dehydrin DHN4-like [*Aegilops tauschii* subsp. tauschii]
TraesCS7A02G479700	−0.4720268	0.8973618	8.5341964	2.223E-14	+	1623	protein_coding	ureide permease 1-like [*Aegilops tauschii* subsp. tauschii]
TraesCS7D02G410000	−3.5602826	0.1359825	9.2735067	1.496E-12	–	483	protein_coding	uncharacterized protein LOC109747823 [*Aegilops tauschii* subsp. tauschii]
TraesCS1D02G256500	−0.8709822	0.7113897	8.089545	3.646E-16	–	1734	protein_coding	AAA-ATPase ASD, mitochondrial-like [*Aegilops tauschii* subsp. tauschii]
novel.1311	−0.3811944	0.8752142	7.8617085	4.704E-11	–	1082	–	–
TraesCS4A02G229900	−2.8641966	0.1793838	9.4469266	1.73E-17	–	1056	protein_coding	2-alkenal reductase (NADP(+)-dependent)-like [*Aegilops tauschii* subsp. tauschii]
TraesCS6D02G379100	−0.4473872	0.9126941	9.285282	5.708E-09	+	892	protein_coding	cytokinesis protein sepA-like [*Aegilops tauschii* subsp. tauschii]
TraesCS4D02G031900	−2.2373153	0.5800992	11.17321	2.562E-13	+	675	protein_coding	oxalate oxidase GF-2.8-like [*Aegilops tauschii* subsp. tauschii]
TraesCS5B02G444500	1.3215179	0.0061584	−7.8212775	1.254E-28	–	1385	protein_coding	tryptophan synthase alpha chain-like isoform X2 [*Aegilops tauschii* subsp. tauschii]
TraesCS4D02G032100	1.9851433	0.6192703	8.6283703	9.134E-12	+	552	protein_coding	oxalate oxidase 2-like [*Aegilops tauschii* subsp. tauschii]
TraesCS7A02G353400	−2.2761827	0.5731962	7.0126884	0.0729095	–	1229	protein_coding	predicted protein [*Hordeum vulgare* subsp. vulgare]
TraesCS7A02G132900	−0.5648719	0.3073534	−7.6105157	9.669E-09	–	1156	protein_coding	uncharacterized protein LOC109766650 [*Aegilops tauschii* subsp. tauschii]
TraesCS3D02G429900	−4.9767719	0.0135991	9.7845788	1.926E-08	+	1033	protein_coding	predicted protein [*Hordeum vulgare* subsp. vulgare]
TraesCS4A02G181900	−2.2373153	0.5800992	10.354153	4.923E-16	+	657	protein_coding	oxalate oxidase 1-like [*Aegilops tauschii* subsp. tauschii]
TraesCS3B02G150500	−2.2761827	0.5731962	10.036526	2.24E-05	+	1251	protein_coding	unnamed protein product [*Triticum aestivum*]
TraesCS2D02G500300	−3.5536974	0.351769	7.1052719	2.051E-05	–	1421	protein_coding	anthocyanidin reductase ((2S)-flavan-3-ol-forming)-like isoform X1 [*Aegilops tauschii* subsp. tauschii]
TraesCS5B02G014700	−3.0629216	0.0017365	−7.050839	2.163E-06	+	1879	protein_coding	zingiberene synthase-like [*Aegilops tauschii* subsp. tauschii]
novel.2216	−4.8156021	0.1002409	7.7308538	2.932E-08	–	1586	–	uncharacterized protein LOC109734236 [*Aegilops tauschii* subsp. tauschii]
TraesCS2A02G203200	2.71469	0.4930462	7.3240261	0.0002701	–	2124	protein_coding	internal alternative NAD(P)H-ubiquinone oxidoreductase A1, mitochondrial-like [*Aegilops tauschii* subsp. tauschii]
TraesCS4B02G225200	−4.2311915	0.1972684	8.2760113	6.72E-09	–	1362	protein_coding	glutathione-S-transferase Cla47 [*Triticum aestivum*]
TraesCS2A02G345500	0.3526547	0.8896238	7.3070073	3.626E-08	–	1906	protein_coding	predicted protein [*Hordeum vulgare* subsp. vulgare]
TraesCS5A02G016700	−2.7946233	0.0206792	−8.1103256	0.0001979	+	951	protein_coding	zingiberene synthase-like [*Aegilops tauschii* subsp. tauschii]
TraesCS3A02G508600	2.7212556	0.4405509	7.2184615	4.862E-18	+	1692	protein_coding	3-oxo-Delta(4,5)-steroid 5-beta-reductase-like [*Aegilops tauschii* subsp. tauschii]
TraesCS2A02G318800	−4.7810317	0.0251074	7.0633853	0.070853	+	1012	protein_coding	hypothetical protein TRIUR3_18331 [*Triticum urartu*]
TraesCS3D02G478200	−4.6901928	0.1176666	8.467173	0.0015332	+	1253	protein_coding	predicted protein [*Hordeum vulgare* subsp. vulgare]
TraesCS4B02G133900	−2.9339392	0.4294896	9.1794596	1.633E-11	+	1007	protein_coding	uncharacterized protein LOC109779747 [*Aegilops tauschii* subsp. tauschii]
TraesCS7B02G257300	1.5900346	0.0047867	−7.9804242	2.369E-10	+	1653	protein_coding	acyl transferase 10-like [*Aegilops tauschii* subsp. tauschii]
TraesCS5D02G403700	−8.7207991	1.321E-06	7.2045543	0.0008243	+	1786	protein_coding	UDP-glycosyltransferase 83A1-like [*Aegilops tauschii* subsp. tauschii]
TraesCS6A02G305000	−3.6678672	0.2339607	10.061734	2.816E-06	+	1737	protein_coding	putative laccase-9 [*Aegilops tauschii* subsp. tauschii]
TraesCS5B02G491800	−3.6368507	0.3312656	7.8327116	6.219E-09	+	866	protein_coding	actin-depolymerizing factor 3-like [*Aegilops tauschii* subsp. tauschii]
TraesCS1B02G470900	−1.2757024	0.7506262	8.1538058	9.302E-10	+	838	protein_coding	calmodulin-like protein 7 [*Aegilops tauschii* subsp. tauschii]
novel.9932	−3.6262911	0.1751248	7.1851142	1.005E-06	–	524	–	–
TraesCS3B02G529400	− 2.2731666	0.4975801	8.1088126	0.0007766	–	1250	protein_coding	glucan endo-1,3-beta-glucosidase GII-like [*Aegilops tauschii* subsp. tauschii]
TraesCS2D02G483300	1.9985882	0.6168275	7.1119124	1.303E-11	+	1891	protein_coding	organic cation/carnitine transporter 4-like [*Aegilops tauschii* subsp. tauschii]
TraesCS5A02G016600	−2.9159243	0.006354	−7.3438681	4.262E-06	+	1374	protein_coding	(E)-beta-farnesene synthase [*Triticum urartu*]
TraesCS1D02G338000	0.019253	0.9768362	−9.1546773	1.11E-06	–	1323	protein_coding	acetylserotonin O-methyltransferase 1-like isoform X1 [*Aegilops tauschii* subsp. tauschii]
TraesCS4A02G279300	1.4134962	0.7262676	7.4179894	8.542E-11	+	657	protein_coding	oxalate oxidase 1-like [*Aegilops tauschii* subsp. tauschii]
TraesCS5B02G337000	−3.5536974	0.351769	7.0909643	3.366E-06	+	1326	protein_coding	putrescine hydroxycinnamoyltransferase-like [*Aegilops tauschii* subsp. tauschii]
novel.12863	3.5517969	0.0975663	−7.8431141	2.666E-05	+	3595	–	uncharacterized protein LOC109734495 [*Aegilops tauschii* subsp. tauschii]
TraesCS1D02G415300	−2.7955134	7.908E-16	−7.0148282	3.915E-07	+	925	protein_coding	uncharacterized protein LOC109761946 [*Aegilops tauschii* subsp. tauschii]
TraesCS3A02G488200	−1.9883298	0.5725568	8.4907608	1.019E-06	–	915	protein_coding	glutathione S-transferase U8-like [*Aegilops tauschii* subsp. tauschii]
TraesCS3B02G529300	−3.4407584	0.2129746	7.4717551	0.0007694	–	1008	protein_coding	glucan endo-1,3-beta-glucosidase GII-like [*Aegilops tauschii* subsp. tauschii]
TraesCS3B02G015800	−2.0138033	0.0021161	−7.6639588	6.058E-07	+	528	protein_coding	unnamed protein product [*Triticum aestivum*]
TraesCS6D02G310700	−1.3927409	0.6634596	7.2448711	6.325E-05	+	1331	protein_coding	flavonol synthase/flavanone 3-hydroxylase-like [*Aegilops tauschii* subsp. tauschii]
TraesCS1A02G435200	−3.2324464	0.4162502	7.8853925	6.338E-08	+	727	protein_coding	calmodulin-like protein 3 [*Aegilops tauschii* subsp. tauschii]
novel.2568	−2.6996636	0.3162526	8.1539415	2.66E-09	+	497	–	–
TraesCS2B02G396000	−0.6556561	0.6785371	−7.4198467	0.057726	+	1462	protein_coding	GA2ox-A6 [*Triticum aestivum*]
TraesCS2D02G316300	−3.2324464	0.4162502	7.3693968	1.063E-06	+	913	protein_coding	unnamed protein product [*Triticum aestivum*]
TraesCS5B02G398800	−5.783603	0.0011091	9.0603748	1.614E-11	+	1670	protein_coding	UDP-glycosyltransferase 83A1-like [*Aegilops tauschii* subsp. tauschii]
TraesCS6D02G165300	−2.8191138	0.4808383	7.2748317	5.452E-07	+	147	protein_coding	hypothetical protein TRIUR3_24014 [*Triticum urartu*]
TraesCS3D02G496600	−4.4309627	0.0880171	7.3117017	1.307E-06	–	866	protein_coding	probable NADPH:quinone oxidoreductase 1 [*Aegilops tauschii* subsp. tauschii]
TraesCS1D02G190500	−3.3683273	0.3961937	8.0287573	3.073E-08	+	962	protein_coding	probable glutathione S-transferase GSTU6 [*Aegilops tauschii* subsp. tauschii]
TraesCS4B02G178300	−2.3142899	0.566463	7.0408433	2.203E-07	–	1870	protein_coding	uncharacterized protein LOC109762516 [*Aegilops tauschii* subsp. tauschii]
TraesCS6B02G074100	−3.5113134	0.0913707	7.3442365	3.653E-08	+	1614	protein_coding	cytochrome P450 76 M5-like [*Aegilops tauschii* subsp. tauschii]
TraesCS6D02G244600	−2.8468712	0.453278	7.3616271	5.384E-06	–	1777	protein_coding	7-deoxyloganetic acid glucosyltransferase-like [*Aegilops tauschii* subsp. tauschii]
TraesCS7D02G418200	−3.2324464	0.4162502	7.1218338	4.271E-06	–	2810	nontranslating_CDS	wall-associated receptor kinase 1-like [*Aegilops tauschii* subsp. tauschii]

**Fig. 5:**
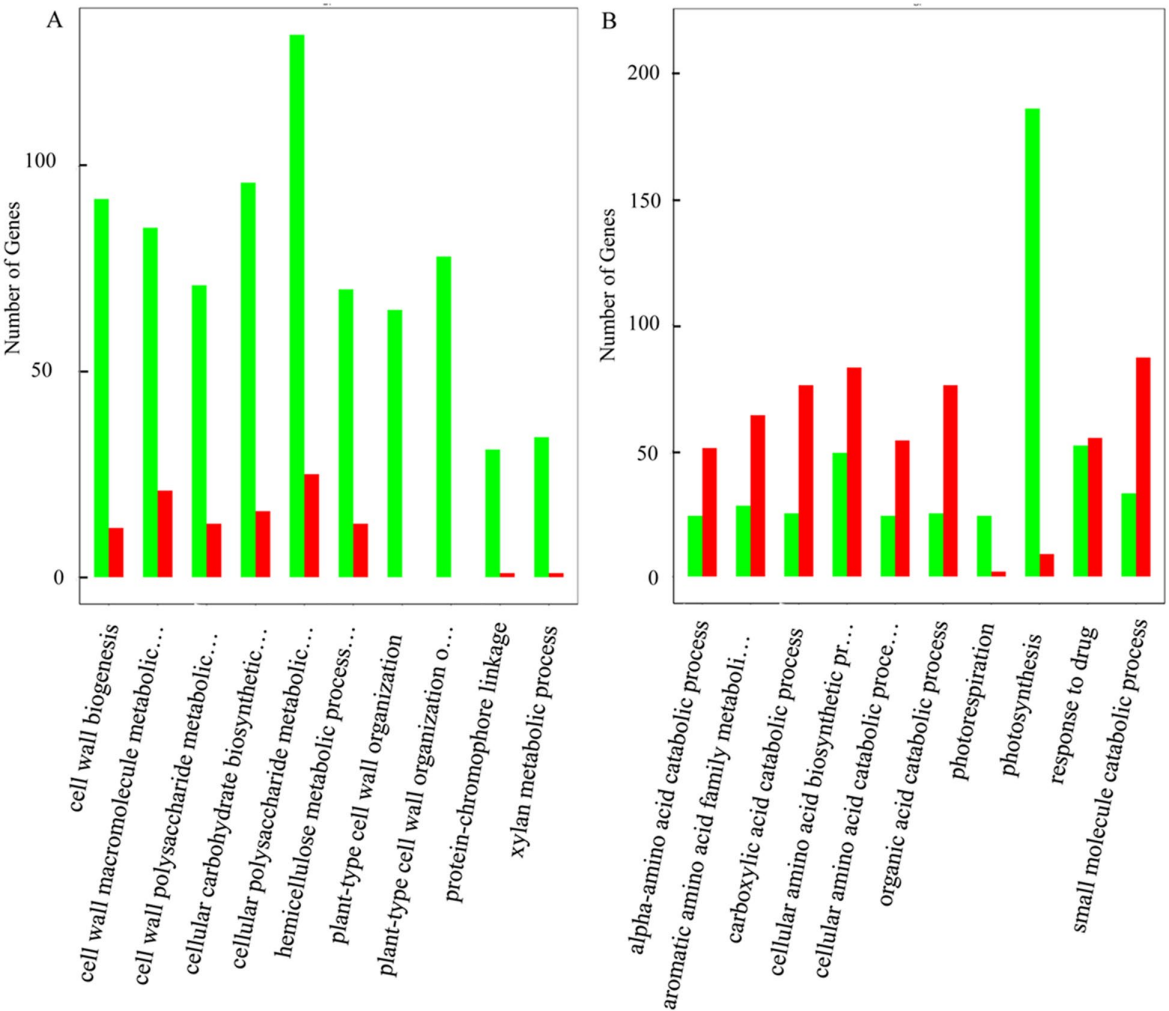
GO enrichment of differentially expressed genes (DEGs) based on biological process, for the comparison of NaCl treatment versus 3-MA + NaCl treatment. **A** was GO enrichment of DEGs in wheat roots (TMG vs TG), and **B** was GO enrichment of DEGs in wheat leaves (TMY vs TY)

**Fig. 6:**
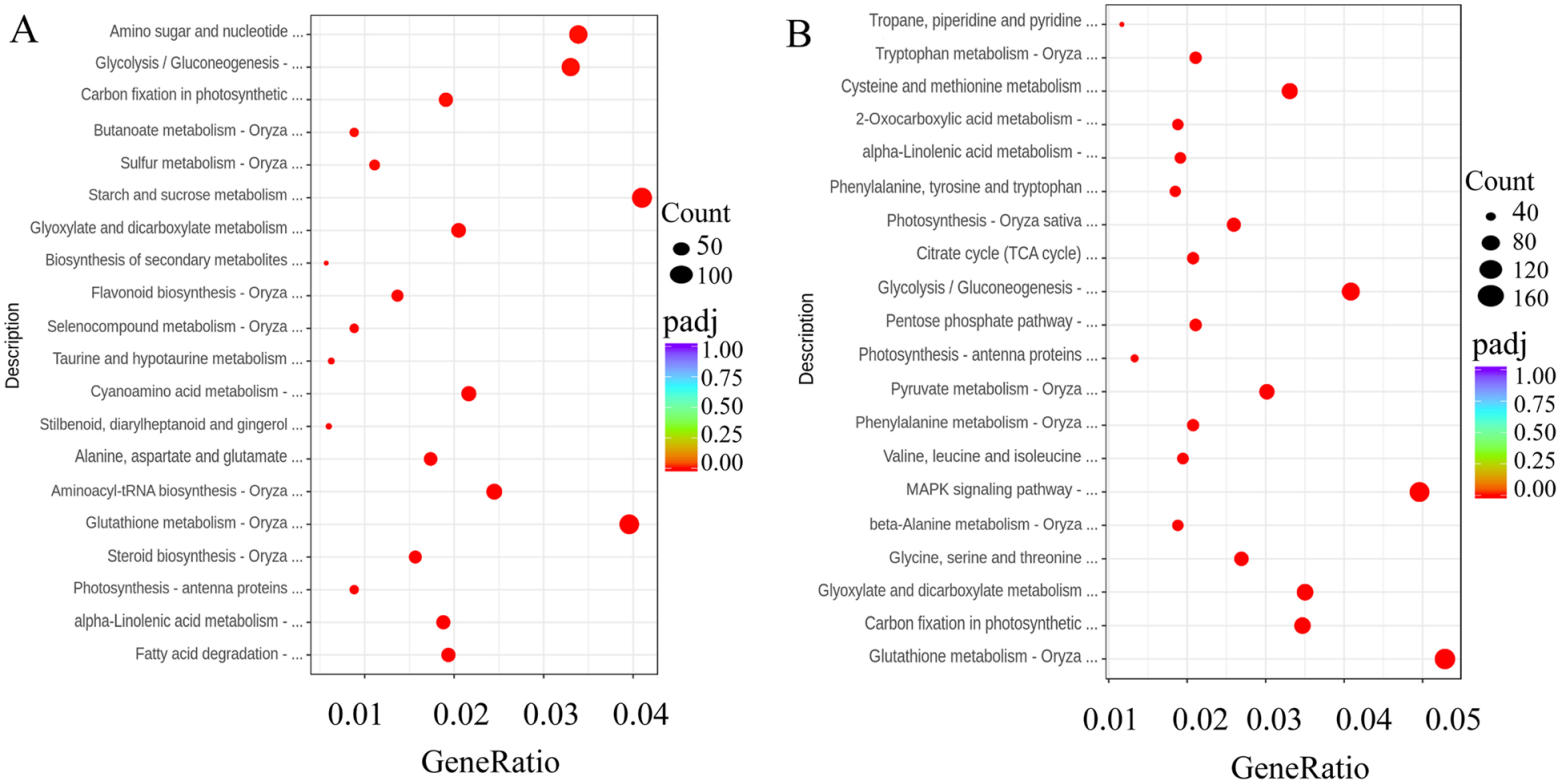
The effect if 3-MA on differentially expressed genes (DEGs) with Kyoto Encyclopedia of Genes and Genomes (KEGG) pathway enrichment (top 20) in roots (**A**) and leaves (**B**) of wheat seedlings in response to salt stress

DEGs related to the biological process (BP) category were the most informative in the context of the salt stress response, and we paid much attention to the DEGs expressed in this category when performing GO annotation. The most represented BP subcategories in the roots of wheat seedlings were cellular polysaccharide metabolic process (GO:0044264), carbohydrate biosynthetic process (GO:0016051), glucan metabolic process (GO:0044042), response to biotic stimulus (GO:0009607), ect. (Fig. 5A, Supplementary Table S5). The most represented BP subcategories in the leaves of wheat seedlings were photosynthesis (GO:0015979), drug catabolic process (GO:0042737), cellular amino acid biosynthetic process (GO:0008652), response to external stimulus (GO:0009605), ect. (Fig. 5B, Supplementary Table S6). The DEGs were also subjected to KEGG pathway enrichment analysis. The DEGs in the roots were mainly enriched in ‘Starch and sucrose metabolism’ (144, 4.1%), ‘Glutathione metabolism’ (139, 4%), MAPK signaling pathway-plant (133, 3.8%), ect. (Fig. 6A, Supplementary Table S7). The DEGs in the leaves were mainly enriched in ‘Glutathione metabolism’ (163, 5.3%), ‘MAPK signaling pathway - plant’ (153, 5%) and ‘Glycolysis/Gluconeogenesis’ (126, 4.1%) (Fig. 6B, Supplementary Table S8). These results suggest that the significant difference in salt tolerance between with and without 3-MA addition could emanate from the difference in the number of genes enriched in each of those shared GO terms, which suggests a potential role of 3-MA in aggravating salt stress.

To comprehensively understand the effect of exogenous 3-MA on wheat transcriptomes under salt stress, the DEGs that showed significant expression divergence between the control and NaCl-treated samples were identified, and the expression levels of these DEGs among the control, NaCl- and NaCl+ 3-MA-treated samples were compared. The results suggested that the addition of 3-MA altered the transcription level of DEGs in roots and leaves of wheat seedlings underwent salt stress. Compared to the controls, the expression of the upregulated DEGs was higher and the expression of the downregulated DEGs was lower in 3-MA-treated plants under NaCl stress conditions (Supplementary Tables S9 and S10).

### Metabolic profiling of wheat roots and leaves at the seedling stage in response to NaCl stress

In the present study, 1394 metabolites were detected in roots and leaves of wheat seedlings. These metabolites included various primary and secondary metabolites, such as amino acids, lipids, organic acids, polyols and sugars, alkaloids, amines, flavonoids, terpenoids etc. Compared with the control (CG or CY), 297 and 247 differentially expressed metabolites (DEMs) were found in roots and leaves induced by salt stress.

**Fig. 7:**
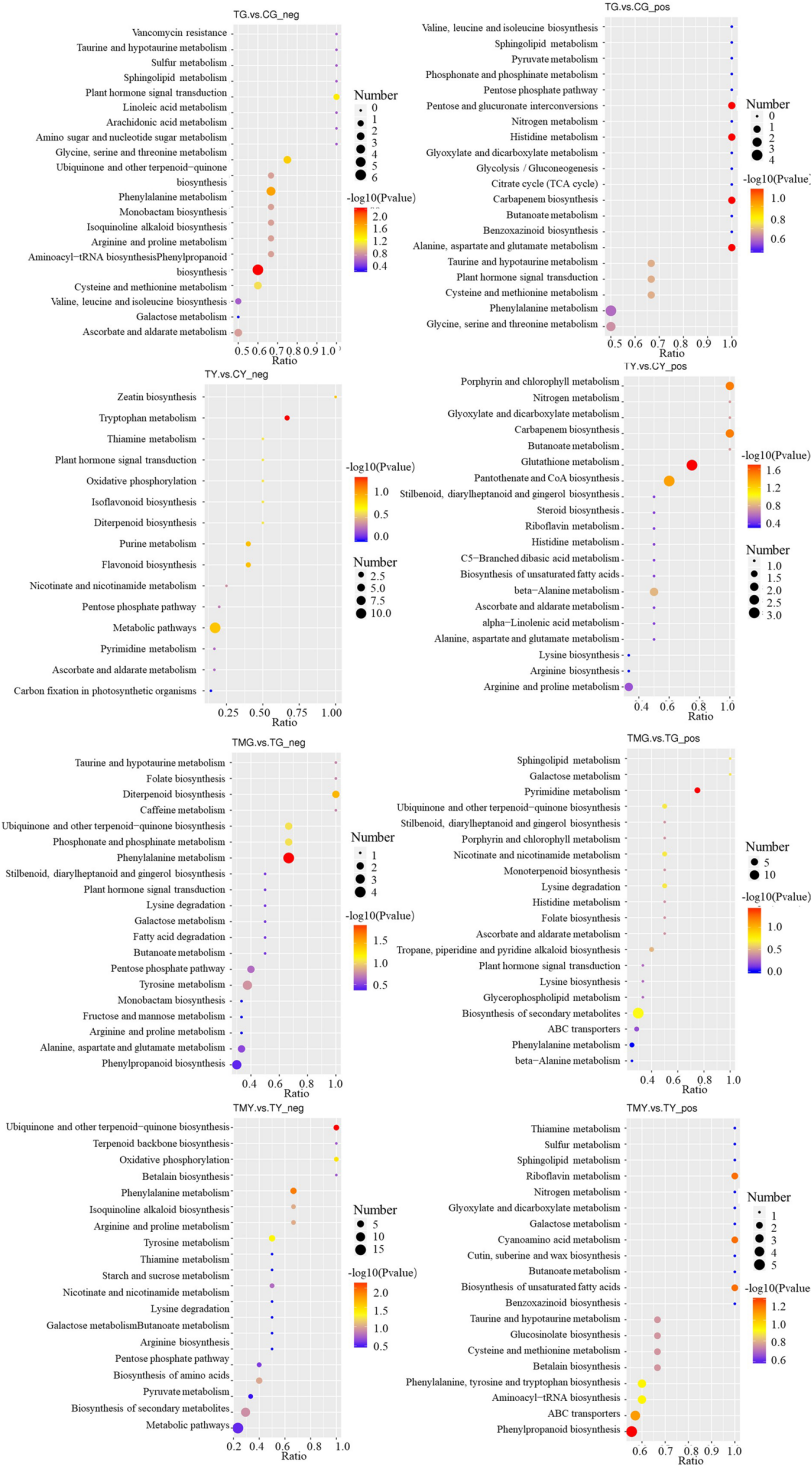
The top 20 significantly enriched KEGG pathways of DEMs. Note: The Y-axis on the left represents KEGG pathways, and the X-axis indicates the “enrich factor” represented by the ratio of DEMs numbers to total annotated gene numbers of each pathway. Low q-values are shown in the blue circle, and high q-values are shown in the red circle. The area of a circle represents DEMs number. CG: the control wheat roots, TG: 150 mM NaCl treated wheat roots, CY: the control wheat leaves, TY: 150 mM NaCl treated wheat leaves. TMG: 5 mM 3-MA + 150 mM NaCl treated wheat roots, TMY: 5 mM 3-MA + 150 mM NaCl treated wheat leaves

Then PCA was performed to detect whether there were differences between the metabolic profiles of the treated and control plants. Clear separation among the NaCl-treated group, 3-MA + NaCl-treated group and controls could be observed. The biological replicate plots clustered together showing good repeatability (Supplementary Fig. 4). There were many significant DEMs detected in the roots and leaves of wheat seedlings induced by salt stress, including metabolites that were up/downregulated (134/113) in the leaves, and metabolites that were up/downregulated (159/148) in the roots (Supplementary Fig. 5). The DEMs in the roots of wheat seedlings induced by salt stress were mainly enriched in metabolic pathways, including phenylpropanoid biosynthesis, phenylalanine metabolism, glycine, serine and threonine metabolism, cysteine and methionine metabolism, and histidine metabolism. The DEMs in the leaves of wheat seedlings induced by salt stress were mainly enriched in glutathione metabolism, carbapenem biosynthesis, metabolic pathways, pantothenate and CoA biosynthesis, flavonoid biosynthesis, tryptophan metabolism, and porphyrin and chlorophyll metabolism. These metabolic pathways may confer salt adaptability to roots and leaves of wheat seedlings (Fig. 7).

### Effect of 3-MA on the metabolome of wheat under salt stress

Exogenous 3-MA also changed the metabolomics of salt-stressed seedlings. In total, 67 increased or decreased metabolites were shared by both NaCl-treated groups (TG/CG) and 3 + MA + NaCl-treated groups (TMG/TG), representing the core metabolites in the two groups of wheat roots under salt stress. These core metabolites contained 39 significantly increased metabolites, including 10 organic acids, 12 free amino acids, 7 polyols and sugars, 7 fatty acids, 3 alkaloids and abscisic acid (ABA), etc., which have vital functions in the effect of 3-MA on osmotic regulation and contribute to the regulation of wheat root tolerance to NaCl stress (Supplementary Table S11). Fifty DEMs were shared by both NaCl-treated groups (TY/CY) and 3 + MA + NaCl-treated groups (TMY/TY), representing the core metabolites in the two groups of wheat leaves under salt stress (Supplementary Table S12). Among them, the contents of 7 organic acids, 7 amino acids, 4 polyols and sugars, 4 fatty acids, 1 alkaloid and jasmonic acid (JA), etc. were significantly increased in both groups of wheat leaves under salt stress, which have vital functions in osmotic regulation and contribute to wheat leaf tolerance to NaCl stress. The DEMs induced by NaCl treatment tended to show higher deviations in their content than those in the control (CG or CY) when exogenous 3-MA was added. The expression of the upregulated DEMs was higher and the expression of the downregulated DEMs was lower in 3-MA-treated plants under NaCl treatment.

In addition to salt stress, many DEMs were also specifically expressed after the addition of 3-MA. In particular, the 185 DEMs in 3-MA-treated wheat root under NaCl stress included 24 organic acids, 13 amino acids, 21 polyols and sugars, 25 fatty acids, 3 alkaloids, etc. Notably, the contents of N, N-dimethyl-9H-purin-6-amine and neosaxitoxin increased by 8.5-fold and 6.36-fold in 3-MA-treated plant roots under NaCl stress, respectively (Supplementary Table S13). The 186 DEMs in 3-MA- treated wheat leaves under NaCl stress included 38 organic acids, 22 amino acids, 15 polyols and sugars, 7 fatty acids, 3 alkaloids, etc. Notably, the contents of N, N-dimethyl-9H-purin-6-amine and 1-methylguanine increased by 10.2-fold and 9.36-fold in 3-MA-treated plant leaves under NaCl stress, respectively. Exogenous 3-MA induced a lower accumulation of organic acids, fatty acids, sugars, ect. (Supplementary Table S14). These DEMs were mainly enriched in amino acid metabolism, phenylalanine metabolism, carbohydrate metabolism, carbapenem biosynthesis, and pantothenate and CoA biosynthesis, indicating that these metabolic pathways are involved in in stress signaling and responses in wheat roots and leaves under NaCl stress with or without 3-MA (Fig. 7).

### Validation in the DEGs and DEMs identified from the transcriptomic and *metabolomic* data

Six DEGs and four DEMs which were involved in the pathway regulated autophagy were chosen to test the reliability of the transcriptomic and metabolomic data. The results showed that the relative gene expression levels of these six genes in roots and leaves of wheat seedlings induced by salt stress were consistent with those acquired in the RNA-seq analysis (Supplementary Table S15). The activities of peroxidase (POD), superoxide dismutase (SOD) and catalase (CAT) in the roots and leaves of wheat seedlings were lower than those in the roots and leaves of 3-MA-treated seedlings and WT seedlings (Supplementary Fig. 6). Similar patterns were found for the 4-Aminobutyric acid (GABA) content (Supplementary Fig. 6), which were in agreement with the metabolomic data.

### Integrated transcriptomic and metabolomic analyses

**Fig. 8:**
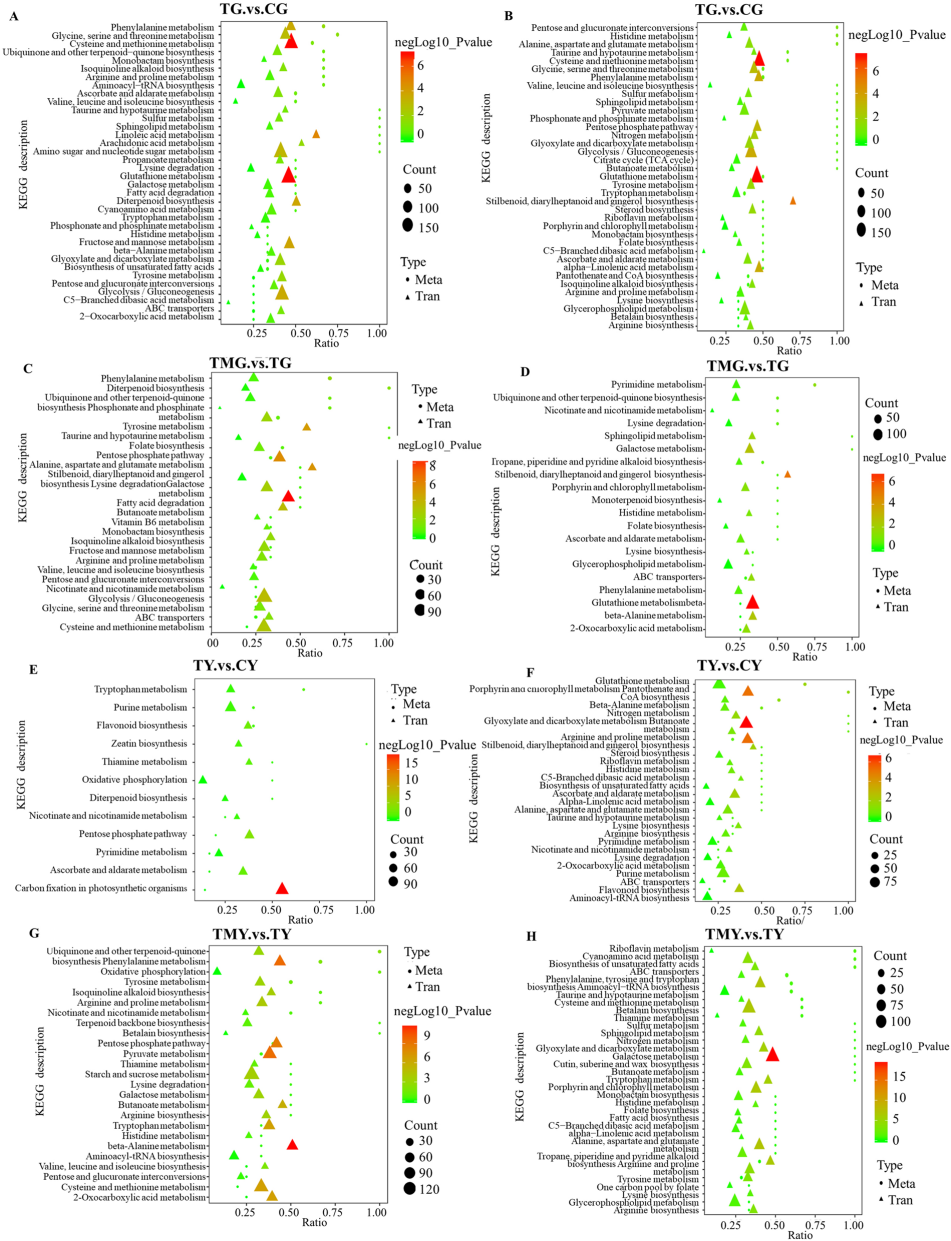
The effect of 3-MA on correlation analysis of Kyoto Encyclopedia of Genes and Genomes (KEGG) pathway enrichment of both genes and metabolites regulated by NaCl stress in roots and leaves of wheat seedlings. Note: The Y-axis on the left represents DEGs and DEMs co-mapped KEGG pathways, and the X-axis indicates the “enrich factor” represented by the ratio of DEMs or DEGs numbers to total annotated metabolite or gene numbers of each pathway. Count represented DEGs and DEMs co-mapped KEGG pathways. The number of DEGs or DEMs enriched in the KEGG pathway. A the negative ion mode of CG.vs.TG, B the positive ion mode of CG.vs.TG, C the negative ion mode of TMG.vs.TG, D the positive ion mode of TMG.vs.TG, E the negative ion mode of CY.vs. TY, F the positive ion mode of CY.vs. TY, G the negative ion mode of TMY.vs. TY, H the positive ion mode of TMY.vs. TY

Through integrated analyses of the metabolomes and transcriptomes of wheat roots and leaves, many DEGs encoding key proteins were enriched in either the biosynthesis or degradation processes of vital metabolites, implying their potential roles in regulating wheat tolerance to salt stress with or without the addition of 3-MA (Fig. 8). Notably, a variety of amino acids, organic acids and related genes were enriched in the same metabolic pathway. A total of 45 metabolic pathways were highly enriched, including glycine, serine and threonine metabolism, cysteine and methionine metabolism, phenylalanine metabolism, ubiquinone and other terpenoid-quinone biosynthesis, ect.

Both the DEGs and DEMs in wheat roots and leaves between TMG and TG and between TMY and TY were significantly enriched in amino acid metabolism pathways (e.g., alanine, glutamine, glycine, ect.), suggesting that amino acids functioned in the wheat response to salt stress as osmotic regulatory substances. The expression profiles of the DEGs enriched in amino acid metabolism in the roots and leaves of wheat seedlings are presented in detail in Supplementary Tables S16 and S17. The greatest accumulation of differentially expressed amino acids included cysteinylglycine, tryptophan, asparagine, histamine, leucine, alanine, gamma-aminobutyric acid, ect. Many of the DEGs enriched in the biosynthesis pathway of these amino acids were upregulated in the roots and leaves of wheat seedlings induced by salt stress, and these DEGs may play important roles in maintaining the biosynthesis of some amino acids under salt stress. The GABA content in wheat leaves was increased significantly by salt stress, and decreased significantly in wheat leaves from the TYvsTMY group. GABA is an important derivative of glutamate that is generated by glutamic acid decarboxylase (GAD). The expression of *GADs* showed the same change trend as that for the GABA content. The addition of 3-MA made the DEMs and DEGs involved in these pathways induced by salt stress deviate more from what was observed for the control (CG or CY). 3-MA treatment decreased wheat adaptability to salt stress. This result implies that autophagy may help wheat seedlings improve their tolerance to salt. The results may help us understand the mechanism for 3-MA-mediated plant salt tolerance and provide a theoretical basis for autophagy decreasing wheat adaptability to salt stress.

## Discussion

Autophagy is involved in the degradation and recirculation pathway, which is essential to maintaining cellular homeostasis [46]. It can be induced or enhanced by various environmental stresses and, as a result, it can enhance plant adaptation to these stresses [29, 47, 48]. However, the connection between salt stress-induced autophagy and the underlying regulators remains poorly understood. As a known inhibitor of autophagy in animals and plants, 3-MA was used to block autophagy induced in wheat seedlings in response to salt stress. Our results, showed that 3-MA addition caused strong ROS production, inhibited autophagy and aggravated the deleterious effect of NaCl stress on wheat seedlings. Our results echo those of a previous study that showed that inhibition of autophagy via knockdown of *ATG2* or *ATG7* aggravated the negative effects of NaCl stress on wheat seedlings, leading to an elevated level of toxic ROS [9]. Then, the combination of transcriptome and metabolome analyses was used to identify the DEGs and DEMs involved in the 3-MA-mediated response of wheat to salt stress. The results help deepen the understanding of the 3-MA-regulated salt response in wheat seedlings. However, the underlying regulatory mechanism of 3-MA-induced sensitivity of wheat to salt stress is still unclear.

### Addition of 3-MA regulates TF expression in wheat seedlings in response to salt stress

Salt stress seriously affects many physiological and biochemical processes in plants and results in the alteration of plant metabolism. The present study found many enriched GO terms for DEGs between (TGvsCG) and (TGvsTMG) or between (TYvsCY) and (TYvsTMY), with most involved in cellular metabolism, biological regulation, and response to stimulus. The results were consistent with those of previous studies [25, 49]. TFs, have significant regulatory functions in plant homeostasis under stress. In the present study, many DEGs encoding transcription factors were identified in the roots and leaves of wheat seedlings under salt stress. These DEGs belonged to MYB, WRKY, ERF, bHLH, HBP, TCP, NAC, etc., indicating that there is a complicated transcriptional regulation network in the wheat seedling response to salt stress. All these transcription factors were identified as positive or negative regulators in the modulation of metabolic pathways under abiotic or biotic stress [11, 25, 50]. Some of these DEGs were more or less highly expressed in the wheat roots and leaves induced by the addition of 3-MA under salt stress. These results suggested that 3-MA regulated plant homeostasis under salt stress by regulating the expression of TFs.

### Addition of 3-MA alters ROS homeostasis in wheat seedlings during salt stress

Salt stress always induces ROS (such as H_2_O_2_ and O_2_•^—^) accumulation, which has oxidative stress-induced toxic effects on plants [7]. In addition to their toxic effects, ROS also function as signaling molecules that induce ROS signal transduction and responses in plant cells, comprising an important detoxification-signaling pathway, and are also involved in antioxidant defense responses and the regulation of ROS homeostasis in response to salt/oxidative stress [5, 7]. Autophagy can maintain ROS homeostasis in plants [29]. In the present study, the contents of both H_2_O_2_ and O_2_•^—^ were increased by salt stress and were further increased by the exogenous addition of 3-MA. Our results echo those of a previous study that indicated that 3-MA impaired Xa3/Xa26-mediated resistance to *Xanthomonas oryzae* pv. *oryzae* by promoting the accumulation of H_2_O_2_ in the xylem parenchyma cells and mesophyll cells of rice [51].

To maintain ROS homeostasis under salt stress, many ROS scavenging enzymes and their coding genes were activated. Our results showed that the upregulated genes of *POD*, *SOD*), *CAT*, and *glutathione s-transferase* (GST) genes were annotated, including 3 *CAT* genes in wheat roots (TraesCS4B02G325800, TraesCS4D02G322700, TraesCS7A02G549900), one *CAT* gene in wheat leaves (TraesCS7A02G549900), two *SOD* genes in wheat leaves (TraesCS4A02G390300, TraesCS7A02G048600), 160 *POD* genes in wheat roots (TraesCS7D02G347300, TraesCS1B02G115900, TraesCS1B02G096800, ect.), 92 *POD* genes in wheat leaves (TraesCS2A02G571300, TraesCS2A02G573400, TraesCS2A02G573500, ect.); 55 *GST* genes in wheat roots (TraesCS3D02G445400, TraesCS3D02G133100, TraesCS1B02G194300, ect.), 39 *GST* genes in wheat leaves (TraesCS1A02G186400, TraesCS1A02G016800, TraesCS1D02G190000, ect.); and 2 *GDP-mannose 3, 5-epimerase* (GME) genes in wheat roots (TraesCS7D02G073300, TraesCS7A02G077700). It has been reported that GST prevents ROS accumulation by reducing H_2_O_2_ via glutathione peroxidase activity [52]. POD catalyzes the oxidation of H_2_O_2_ [53]. SOD catalyzes the disproportionation of superoxide radicals to H_2_O_2_, and then H_2_O_2_ is catalyzed into oxygen and water by CAT [54]. GME plays an important role in ascorbic acid (AsA) synthesis in plants, and AsA is an important antioxidant involved in ROS scavenging in plants [55, 56]. 3-MA treatment affected the majority of the above genes more seriously than the control. The results suggested that wheat roots and leaves have a complex antioxidant system, in which these enzymes play essential roles in scavenging ROS and alleviating ROS accumulation under salt stress. 3-MA positively regulates ROS accumulation by more strongly inducing the related genes than the control.

### Addition of 3-MA regulates plant photosynthesis and carbon sequestration in wheat seedlings in response to salt stress

Plant photosynthesis and carbon sequestration are easily affected by salt stress [57]. Autophagy is central to metabolic regulation through its ability to recycle intracellular nutrients, maintain sufficient amino acid and fixed-carbon pools, and eliminate dysfunctional or unwanted proteins, lipids, and organelles [58]. According to our transcriptome analysis, the DEGs in wheat leaves induced by salt stress were mostly enriched in flavonoid biosynthesis, oxidative phosphorylation, diterpenoid biosynthesis, caron fixation in photosynthetic organisms, porphyrin and chlorophyll metabolism, ect. The DEGs in wheat roots induced by salt stress were mostly enriched in amino acid metabolism, sugar metabolism and fat metabolism. Plant salt tolerance is closely related to the accumulation of amino acids and carbohydrates [59]. Our results also indicated 3-MA addition aggravated damage to PSII of wheat seedlings induced by NaCl stress. Therefore, it is of great significance for plants to maintain photosynthetic capacity and normal carbon metabolism under salt stress. By providing energy, signal transduction and osmotic regulation, these metabolites play important roles in the plant response to salt stress. Soluble sugars (including glucose, sucrose, fructose, raffinose ect.) can enhance plant tolerance to salt stress by osmotic regulation to maintain leaf water content [60]. Active carbohydrate metabolism helps plants reach a new equilibrium state in response to salt stress [61]. In the present study, most soluble sugars including stachyose, maltotetraose, glucose, fucose, ect. Were downregulated. Genes related to sugar biosynthesis, glycosylhydrolase, starch decomposition and sugar transport were identified as DEGs to salt stress. Most of the DEGs related to carbohydrate transportation and metabolism showed the same expression trend in roots and leaves, and the expression of these DEGs in the roots was greater than that in the leaves. 3-MA addition affected the majority of the above DEGs and DEMs more seriously than the control. These results suggested that salt stress caused intracellular nutrient disorders/stress, which induced autophagy to degrade unwanted metabolites or damaged intracellular components and organelles to provide raw materials for nutrient synthesis.

### 3-MA readjusts the metabolic balance of wheat seedlings under salt stress

Transcriptome and metabolome analyses indicated that the process of secondary metabolism synthesis and decomposition was significantly enriched for DEGs and DEMs in wheat roots and leaves under salt stress. It was found the amino acid metabolism pathway was enriched with the largest number of the DEGs and DEMs in wheat roots and leaves under salt stress, indicating that amino acid metabolism may have important biological functions in the wheat response to salt stress. This result is consistent with the results obtained in buckwheat under salt stress [8]. Amino acids are precursors of functional macromolecular proteins and important nitrogen metabolites in plants [39]. The free amino acids in plant cells can be directly or indirectly adjusted to quickly adapt to environmental changes. Our results showed that most amino acids in wheat roots and leaves decreased under150 mM NaCl stress, the contents of metabolites related to glycolysis decreased, and the contents of the trans amino metabolite asparagine decreased. Glutamate has an important function in nitrogen metabolism. Glutamate is usually used to synthesize other amino acids and nitrogenous compounds. Glutamate dehydrogenase can catalyze the reduction of α-ketoglutarate to glutamic acid in a high-NH4^+^ environment [62]. The accumulation of glutamate in roots and leaves of wheat seedlings decreased, indicating that ammonia conversion in wheat was reduced under salt stress and that wheat seedling growth was inhibited. Many studies have shown that GABA will be synthesized in large quantities in plants under salt stress (Khanna et al., 2021). Li et al. (2020) indicated that the overexpression of *MdATG18a* in apple enhances alkaline tolerance and the GABA shunt, which may be due to the increase in autophagic activity [63]. Our results also showed that salt stress induced an increase in the GABA content in leaves of wheat seedlings, which was decreased significantly by 3-MA. However, the GABA content in wheat roots showed no significant change. As a nonprotein, four-carbon amino acid, the biochemical properties of GABA are similar to those of some infiltrating molecules such as proline and betaine, which can be used as osmotic regulation substances to reduce cell water potential and improve water holding capacity, slowing down the damage to plant cells caused by osmotic stress [64]. These results indicated that 3-MA altered wheat leaf tolerance to salt stress by decreasing the GABA content, which inhibited stress signals and reduced stress.

It was found that abscisic acid (ABA) regulates the accumulation of GABA under salt stress [65]. We also found that ABA and jasmonic acid (JA) were induced and synthesized quickly in wheat roots and leaves. ABA has a sesquiterpene structure [66]. The ABA content in normal plants is low but can be increased rapidly under stress [65]. The ABA signaling pathway is involved in regulating plant growth and the plant response to salt stress. Amjad et al. (2014) found that the ABA in a salt-tolerant tomato genotype was increased significantly by salt stress, and an increased ABA concentration helped tomato plants resist salt stress by inducing a decrease in Na^+^ accumulation and maintaining osmotic balance [67]. Our results indicated that salt stress induced an increase in ABA accumulation in wheat roots and leaves. The abscisic acid 8′-hydroxylase 1-like genes that participate in the oxidative degradation of ABA were downregulated in wheat roots. Furthermore, ABA could be released from vacuoles and apoplast stores of ABA glucosyl ester to be catalyzed by beta-glucosidase [68]. Some beta-glucosidase genes were upregulated in wheat roots and leaves under salt stress. ABA also leads to the induction of autophagy under stress [18]. Furthermore, there is crosstalk between the JA signaling pathway and the ABA pathway through transcription factors including MYC2, ABI5, and WRKY57 [69]. JA positively regulated maize tolerance to salt stress via involvement in Na^+^ transportation from the roots to the shoots [50]. 3-MA addition also affected the majority of the above DEGs and DEMs seriously than the control. Calmodulin-like proteins are a remarkable group of putative Ca^2+^ sensors in plants that participate in regulating the SA, JA, and ABA signaling pathways under biotic or abiotic stress [70]. The expression of 4 calmodulin-like genes in wheat leaves was decreased significantly by 3-MA addition under salt stress. These results suggested that sugars, GABA, ABA, JA and Ca^2+^ have positive effects on autophagy in wheat seedlings induced by salt stress.

## Conclusions

The addition of the autophagy inhibitor 3-MA inhibited growth, inhibited autophagy and increased the ROS content of wheat roots and leaves under NaCl stress. A total of 14,759 DEGs and 554 DEMs were identified under NaCl stress. 3-MA addition changed the transcriptome and metabolome in wheat seedlings under salt stress. The expression of the upregulated DEGs and DEMs was higher, and the expression of the downregulated DEGs and DEMs was lower in 3-MA-treated plants under NaCl treatment. This study will contribute to a better understanding of the mechanism by which 3-MA mediates salt tolerance and thus provide a theoretical foundation for autophagy-regulated wheat seedlings responses to salt stress.

## Materials and methods

### Wheat seedling growth and NaCl treatments

The test variety was NaCl-tolerant Jimai 22 which was acquired from the Tianjin Academy of Agricultural Sciences. Jimai 22 is a medium gluten wheat variety with super high yield, multiresistance and high quality. The seeds were first washed with tap water and then rinsed with distilled water three times before being soaked in distilled water for 12 h. The soaked seeds were then placed on moist gauze for germination. Distilled water was regularly sprayed onto the seeds for culture at ambient temperature. The distilled water was changed every 24 h until the seedlings grew to the one-leaf-and-one-bud stage. Uniform seedlings were transplanted into plastic pots containing 1/4 Hoagland solution for further culture. The nutrient solution was changed every 2 d until the seedlings grew to the two-leaf-and-one-bud stage.

The seedlings selected for uniform growth were separately transplanted into plastic pots (50 cm in diameter and 40 cm in height; 10 plants per pot) containing 1/4 Hoagland nutrient solution (pH 5.2) with 0 μM NaCl to form the control group; those subjected to various levels of stress (150 mM NaCl, 5 mM 3-MA, or 5 mM 3-MA+ 150 mM NaCl) formed the treatment groups. 3-MA (Sigma Aldrich, Saint Louis, MO, USA) was added to the medium 5 h prior to NaCl treatment. The nutrient solution was changed every 2 d. For each treatment, seedlings from three pots were collected randomly or measured as a replicate, and each treatment had 3 duplications.

### Determination of plant physiological parameters

Evans blue staining was used to identify dead cells. Deeper staining of more sites indicates less cell activity in the root or leaves. The staining approach was modified based on the methods proposed by Baker (1994) [71] and Chalivendra (2017) [72]. Three seedlings were harvested from both the control and treated-groups at 0 and 4 d. They were washed with tap water, distilled water, and deionized water. The washed-root tips and leaves of wheat seedings were placed in 0.25% Evans blue staining liquid (Solarbio Life Sciences, Cat#: G1810, China) for 8 min and 4 h, respectively in the dark. Then the stained roots and leaves of wheat seedlings were washed and photographed under a stereoscopic microscope (Nikon C-fled2, Nikon, Tokyo, Japan).

The in situ accumulation of H_2_O_2_ and O_2_•^—^ in the roots and leaves of wheat seedlings was detected by histochemical staining with 3,3-diaminobenzidine (DAB, Sigma, USA) and nitro blue tetrazolium (NBT, Sigma, USA), respectively [29]. The contents of H_2_O_2_ and O_2_•^—^ were determined using detection kits manufactured by Solarbio Life Sciences (Cat#: BC3595 and BC1290, China).

The activities of antioxidant enzymes including SOD, POD, and CAT were determined using commercial detection kits according to the manufacturer’s instructions (Solarbio Lifesciences, Cat#: BC0170, BC0090 and BC0200, China).

### RNA-seq analysis

4 d after NaCl and 3-MA treatment, the roots and the third leaves were collected respectively with every 10 of them being mixed into one biological replicate for each treatment with three biological replicates. Each sample was ground into a powder with liquid nitrogen. Then, the RNA in wheat roots and leaves was extracted by using TRIzol reagent (Invitrogen, USA). There were six sample groups: CG: the control wheat roots, TG: 150 mM NaCl treated wheat roots, TMG: 5 mM 3-MA + 150 mM NaCl-treated wheat roots, CY: control wheat leaves, TY: 150 mM NaCl treated wheat leaves, and TMY: 5 mM 3-MA + 150 mM NaCl treated wheat leaves.

The libraries for transcriptome sequencing were generated using the NEBNext® UltraTM RNA Library Preparation Kit for Illumina® (NEB, Cat #E7775, USA), following the manufacturer’s instructions [73]. Then, the library of each sample was sequenced on an Illumina NovaSeq platform (Novogene Bioinformatics Institute, Beijing, China), with a 150 bp paired-end read model [74]. The paired-end clean RNA-seq reads were mapped to the reference genome assembly of Chinese Spring (CS), and the gene model annotation files were downloaded from ftp://ftp.ensemblgenomes.org/pub/release-45/plants/fasta/triticum_aestivum/dna/Triticum_aestivum.IWGSC.dna.toplevel.fa.gz.

The DEGs analysis of every 2 groups was performed using the DESeq2 R package (1.16.1) [75]. Genes with an adjusted *P* < 0.05 and |log2FoldChange| > 0 found by DESeq2 were considered differentially expressed. The datasets generated and analyzed during the current study are available in the National Center for Biotechnology Information (NCBI) Sequence Read Archive (SRA) database (accession number: GSE166260)] repository. [https://www.ncbi.nlm.nih.gov/geo/info/linking.html].

### Quantitative real-time PCR (qRT–PCR) analysis

qRT–PCR was employed to validate the relative gene expression of DEGs as previously described [76]. TRIzol reagent (Invitrogen, USA) was used to extract total RNA from the root and leaf samples. Two micrograms of total RNA were used as a template for first-strand cDNA synthesis via a reverse transcription kit following the protocol provided by the manufacturer (Promega, USA). qRT–PCR was performed on a Roche LightCycler 480 system using SYBR Green PCR master mix (Roche, UK). For DEG expression analysis, quantitative values were obtained using the cycle number (Ct value). The relative expression of each gene was calculated by the 2^-∆∆Ct^ method. The wheat *α-tubulin* gene was used as an internal control [77]. The primers used for qRT–PCR were designed with Primer 3 software; which are listed in Table S18.

### Metabolomic assay

Samples of roots and leaves from each treatment group were collected for RNA-seq assays. Every treatment had four biological replicates. The extraction of metabolites was performed according to the procedure described by Ma et al. (2019) [8]. Sample tissues (0.1 g) were ground to a powder with liquid nitrogen. Liquid chromatography-mass spectrometry (LC–MS) analyses were performed using a Vanquish UHPLC system (Thermo Fisher, USA) coupled with an Orbitrap Q Exactive series mass spectrometer (Thermo Fisher, USA) (Novogene Bioinformatics Institute, Beijing, China). Compound Discoverer 3.1 (CD3.1, Thermo Fisher) was used to perform peak alignment, peak selection, and quantitation for each metabolite. Then, the peaks were matched with the mzCloud (https://www.mzcloud.org/) mzVault and MassList databases to obtain the accurate qualitative and relative quantitative results. Statistical analyses were performed using the statistical software R (R version R-3.4.3), Python (Python 2.7.6 version) and CentOS (CentOS release 6.6). The metabolites with *VIP* > 1, *P* < 0.05 and |log2FoldChange| ≥ 2 or FC ≤ 0.5 were regarded as differential metabolites. Pearson’s correlation analysis was used to integrate metabolome and transcriptome analyses.

### Data analysis

To determine the physiological parameters, at least three replicates were performed. All collected data were statistically analyzed by analysis of variance. Duncan’s multiple range test was used to compare the mean differences. Statistical significance was considered as *P* < 0.05.

## Supplementary Information

**Additional file 1: Supplementary Figure1.** The effect of autophagosomes in roots and leaves of wheat seedlings stained with monodansylcadaverine (MDC) under NaCl stress.

**Additional file 2: Supplementary Figure 2.** The pearson correlation between biological replicates for all samples.

**Additional file 3: Supplementary Figure 3.** GO enrichment of differentially expressed genes (DEGs) based on biological process, cellular component and molecular function categories for the comparison of NaCl treatment versus control conditions.

**Additional file 4: Table S1.** The GO enrichment of DEGs in roots of wheat seedlings under NaCl stress.

**Additional file 5: Table S2.** The GO enrichment of DEGs in leaves of wheat seedlings under NaCl stress.

**Additional file 6: Table S3.** The KEGG pathway significant enrichment in roots of wheat seedlings under NaCl stress.

**Additional file 7: Table S4.** The KEGG pathway significant enrichment in leaves of wheat seedlings under NaCl stress.

**Additional file 8: Table S5.** The effect of 3-MA on GO significant enrichment of DEGs in roots of wheat seedings under NaCl stress.

**Additional file 9: Table S6.** The effect of 3-MA on GO significant enrichment of DEGs in leaves of wheat seedings under NaCl stress.

**Additional file 10: Table S7.** The effect of 3-MA on KEGG pathway significant enrichment in roots of wheat seedings under NaCl stress.

**Additional file 11: Table S8.** The effect of 3-MA on KEGG pathway significant enrichment in leaves of wheat seedings under NaCl stress.

**Additional file 12: Supplementary Table 9.** Genes that were differentially expressed in wheat roots between NaCl and 3-MA+ NaCl samples.

**Additional file 13: Supplementary Table 10.** Genes that were differentially expressed in wheat leaves between NaCl and 3-MA+ NaCl samples.

**Additional file 14: Supplementary Figure 4.** Principal component analysis (PCA) score diagram in (A) negative, (B) positive ion mode of metabolic profiles in the wheat roots and leaves.

**Additional file 15: Supplementary Figure 5.** Volcano plot of negative ion and positive ion mode in roots and leaves of wheat seedlings.

**Additional file 16: Table S11.** The root differentially expressed metabolites (DEMs) were shared by both NaCl-treated roots (TG/CG) and 3-MA + NaCl-treated roots (TMG/TG) of wheat seedlings.

**Additional file 17: Table S12.** The leaves differentially expressed metabolites (DEMs) were shared by both NaCl-treated leaves (TY/CY) and 3-MA + NaCl-treated leaves (TMY/TY) of wheat seedlings.

**Additional file 18: Table S13.** The root differentially expressed metabolites (DEMs) were specific in NaCl-treated roots (TG/CG) and 3-MA + NaCl-treated roots (TMG/TG) of wheat seedlings.

**Additional file 19: Table S14.** The root differentially expressed metabolites (DEMs) were specific in NaCl-treated leaves (TY/CY) and 3-MA + NaCl-treated leaves (TMY/TY) of wheat seedlings.

**Additional file 20: Table S15.** The gene expression verification by qRT-PCR.

**Additional file 21: Supplementary Figure 6.** The effect of 3-MA on activity of POD, SOD, CAT and GABA content in wheat seedlings under NaCl stress.

**Additional file 22: Table S16.** The expression patterns of DEGs assigned to amino acid metabolism were shared by both NaCl-treated roots (TG/CG) and 3-MA + NaCl-treated roots (TMG/TG).

**Additional file 23: Table S17.** The expression patterns of DEGs assigned to amino acid metabolism were shared by both NaCl-treated leaves (TY/CY) and 3-MA + NaCl-treated leaves (TMY/TY).

**Additional file 24: Table S18.** Primers used in qRT-PCR analysis.

## Data Availability

The datasets generated and analyzed during the current study are available in the [National Center for Biotechnology Information (NCBI) Sequence Read Archive (SRA) database (https://www.ncbi.nlm.nih.gov/geo/info/linking.html) under the accession number: GSE166260.

## Abbreviations

3-MA: 3-Methyladenine
DEG: Differentially expressed gene
DEM: Differentially expressed metabolite
PI3K: Phosphatidylinositol 3-kinase
qRT-PCR: Quantitative real-time PCR
ROS: Reactive oxygen species
LC-MS: Liquid chromatograph mass spectrometer
GAD: Glutamic acid decarboxylase
POD: Peroxidase
SOD: Superoxide dismutase
CAT: Catalase
GST: Glutathione s-transferase
AsA: Ascorbic acid
GABA: 4-Aminobutyric acid
ABA: Abscisic acid
JA: Jasmonic acid
CG: The control wheat roots
TG: 150 mM NaCl treated wheat roots
TMG: 5 mM 3-MA + 150 mM NaCl treated wheat roots
CY: The control wheat leaves
TY: 150 mM NaCl treated wheat leaves
TMY: 5 mM 3-MA + 150 mM NaCl treated wheat leaves
TF: Transcription factor
PCD: Programmed cell death
PI3K: Phosphatidylinositol 3-kinase
ATP: Adenosine triphosphate
RNA-seq: RNA sequencing
H_2_O_2_: Hydrogen peroxide
O_2_•^—^: Superoxide
GO: Gene Ontology
KEGG: The Kyoto Encyclopedia of Genes and Genomes
BP: Biological process
GME: GDP-mannose 3, 5-epimerase
Fv/Fm: PSII photochemistry
Y (II): Quantum yield of PSII
qP: Quenching coefficient
ETR: Electron transfer rate
NPQ: Nonphotochemical quenching coefficient
PCA: Principal component analysis
