# Supplementary material for: Comparative transcriptomic and metabolic profiling provides insight into the mechanism by which the autophagy inhibitor 3-MA enhances salt stress sensitivity in wheat seedlings
Source: BMC Plant Biol. 2021 Dec 6;21:577. doi: 10.1186/s12870-021-03351-5 (PMC8647401; doi:10.1186/s12870-021-03351-5)
Supplement: Supplementary file 1 — Additional file 1: Supplementary Figure1. The effect of autophagosomes in roots and leaves of wheat seedlings stained with monodansylcadaverine (MDC) under NaCl stress. [file 12870_2021_3351_MOESM1_ESM.docx]

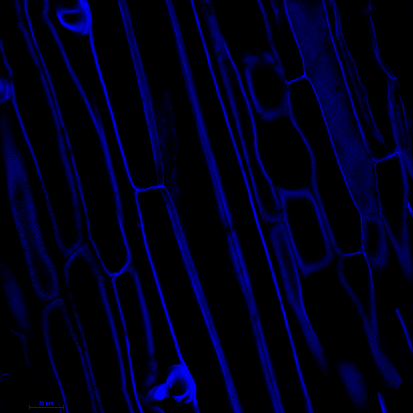

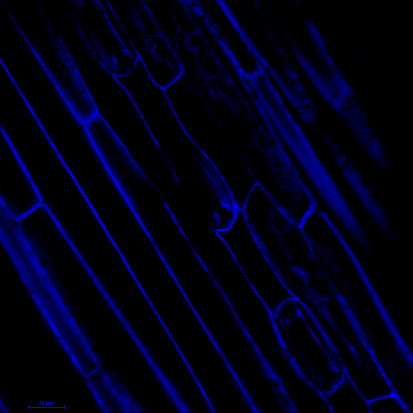

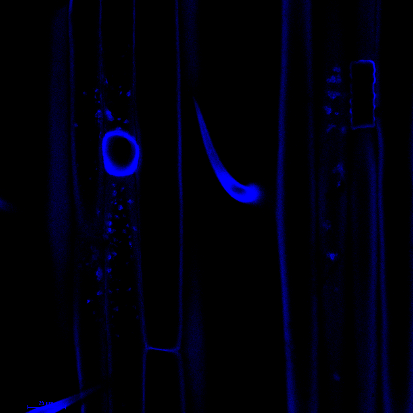

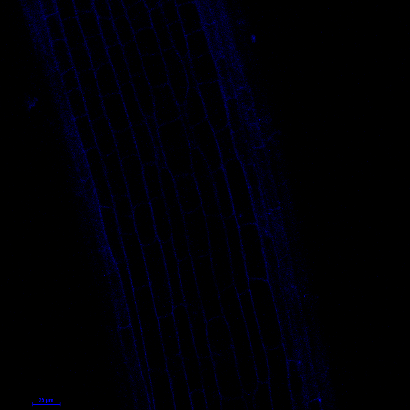

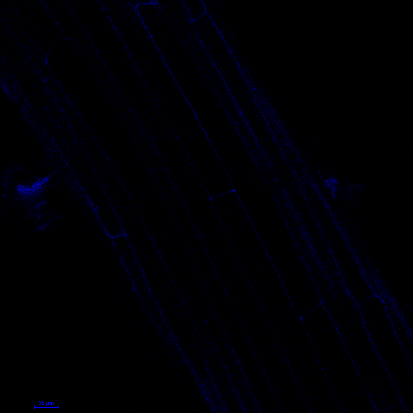

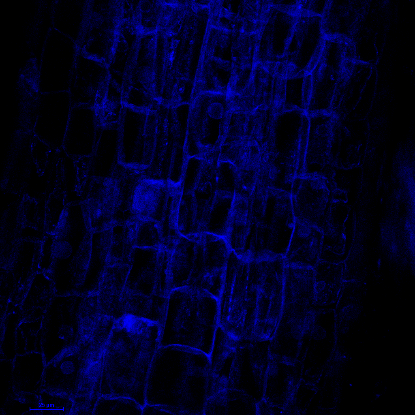

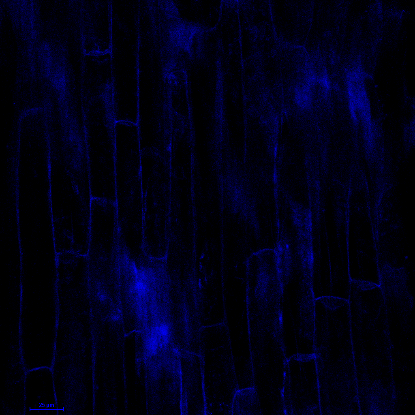


Root

CK

3-MA

NaCl

3-MA+ NaCl


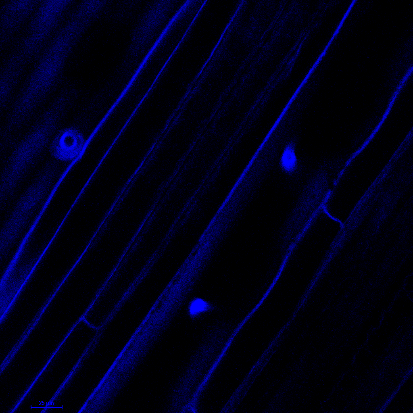


Leaf

Supplementary Figure1 The effect of autophagosomes in roots and leaves of wheat seedlings stained with monodansylcadaverine (MDC) under NaCl stress

Fluorescence dots are autophagosomes.
