# Supplementary material for: Comparative transcriptomic and metabolic profiling provides insight into the mechanism by which the autophagy inhibitor 3-MA enhances salt stress sensitivity in wheat seedlings
Source: BMC Plant Biol. 2021 Dec 6;21:577. doi: 10.1186/s12870-021-03351-5 (PMC8647401; doi:10.1186/s12870-021-03351-5)
Supplement: Supplementary file 2 — Additional file 2: Supplementary Figure 2. The pearson correlation between biological replicates for all samples. [file 12870_2021_3351_MOESM2_ESM.docx]

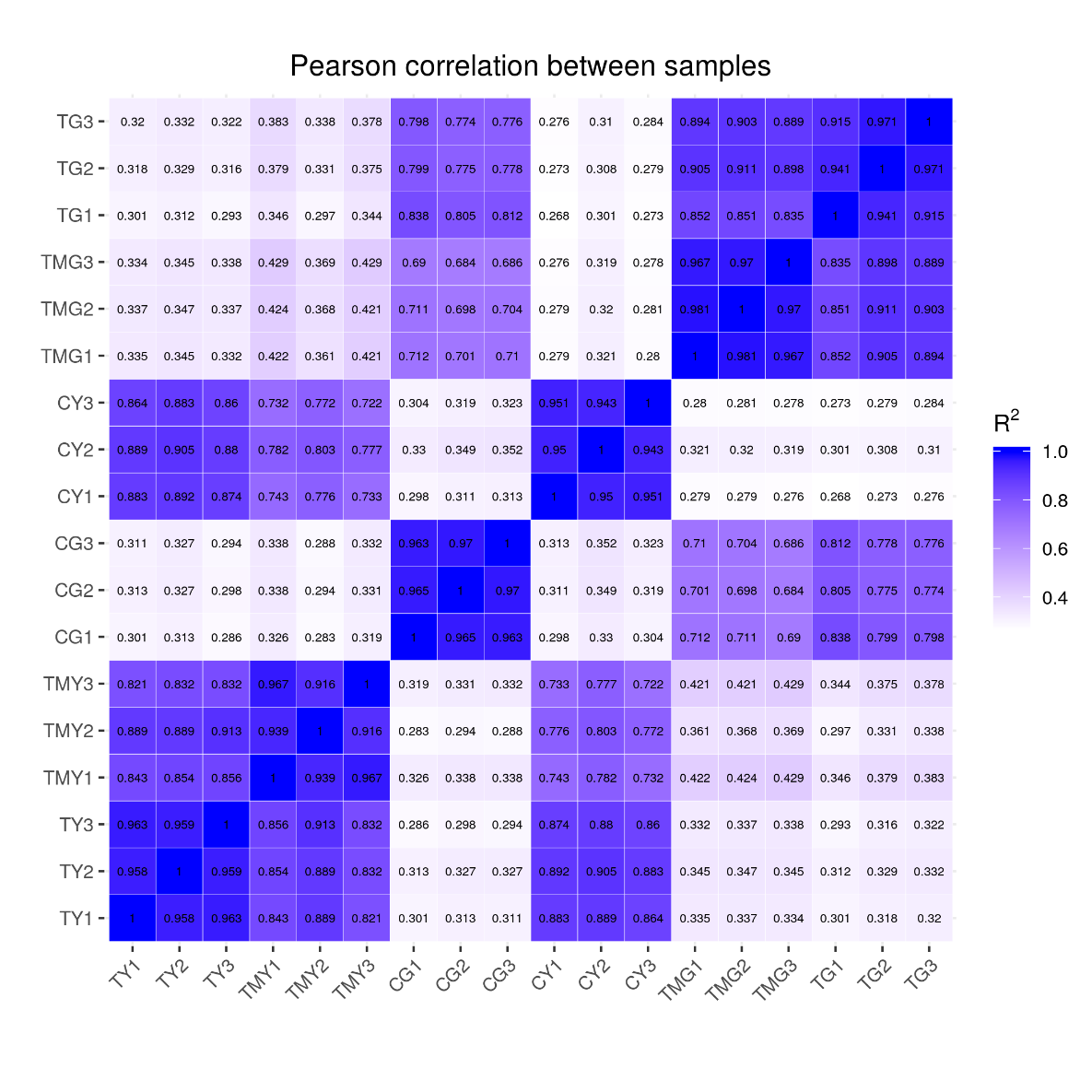


Supplementary Figure 2 The pearson correlation between biological replicates for all samples

Note: CG: the control wheat roots, TG: 150 mM NaCl treated wheat roots, TMG: 5 mM 3-MA + 150 mM NaCl treated wheat roots, CY: the control wheat leaves, TY: 150 mM NaCl treated wheat leaves, TMY: 5 mM 3-MA + 150 mM NaCl treated wheat leaves.
