# Supplementary material for: Comparative transcriptomic and metabolic profiling provides insight into the mechanism by which the autophagy inhibitor 3-MA enhances salt stress sensitivity in wheat seedlings
Source: BMC Plant Biol. 2021 Dec 6;21:577. doi: 10.1186/s12870-021-03351-5 (PMC8647401; doi:10.1186/s12870-021-03351-5)
Supplement: Supplementary file 3 — Additional file 3: Supplementary Figure 3. GO enrichment of differentially expressed genes (DEGs) based on biological process, cellular component and molecular function categories for the comparison of NaCl treatment versus control conditions. [file 12870_2021_3351_MOESM3_ESM.docx]

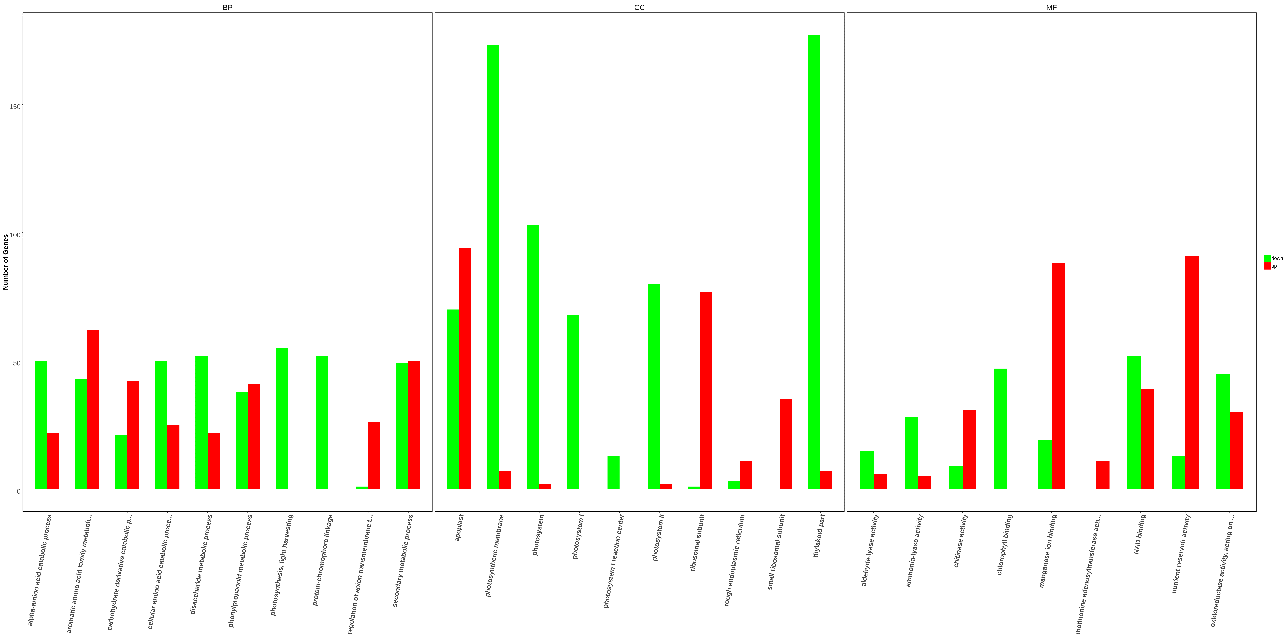


A


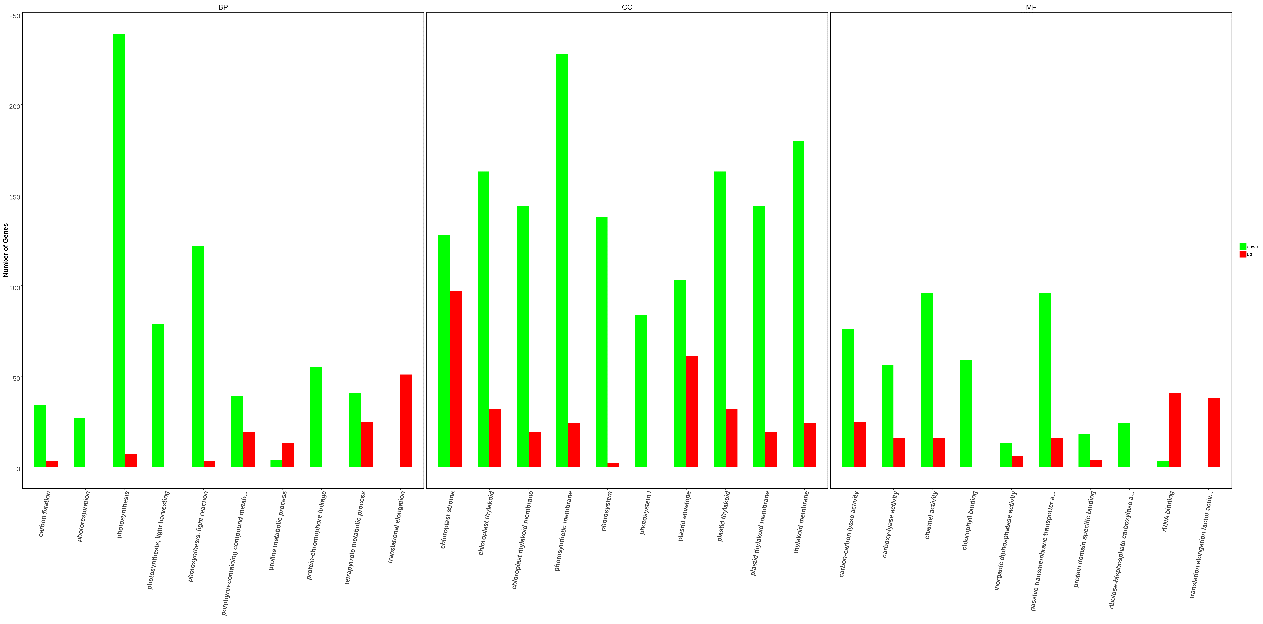


B

Supplementary Figure 3 GO enrichment of differentially expressed genes (DEGs) based on biological process, cellular component and molecular function categories for the comparison of NaCl treatment versus control conditions

A was GO enrichment of DEGs in wheat roots (TG vs CG), and B was GO enrichment of DEGs in wheat leaves (TY vs CY).
