# Supplementary material for: Comparative transcriptomic and metabolic profiling provides insight into the mechanism by which the autophagy inhibitor 3-MA enhances salt stress sensitivity in wheat seedlings
Source: BMC Plant Biol. 2021 Dec 6;21:577. doi: 10.1186/s12870-021-03351-5 (PMC8647401; doi:10.1186/s12870-021-03351-5)
Supplement: Supplementary file 12 — Additional file 12: Supplementary Table 9. Genes that were differentially expressed in wheat roots between NaCl and 3-MA+ NaCl samples. [file 12870_2021_3351_MOESM12_ESM.docx]

Supplementary table 9 Genes that were diﬀerentially expressed in wheat roots between NaCl and 3-MA+ NaCl samples

| gene_id | TGvsCG_log2FoldChange | TGvsCG_pvalue | TMGvsTG_log2FoldChange | TMGvsTG_pvalue | gene_strand | gene_length | gene_biotype | nr annotation |
| --- | --- | --- | --- | --- | --- | --- | --- | --- |
| TraesCS3B02G483500 | 0.07 | 0.854396 | -7.76 | 3.49E-10 | + | 2416 | protein_coding | beta-glucosidase 5-like isoform X1 [Aegilops tauschii subsp. tauschii] |
| TraesCS2A02G527700 | 0.64 | 0.155393 | -8.31 | 1.04E-11 | + | 1724 | protein_coding | chalcone synthase [Oryza officinalis] |
| TraesCS7D02G469600 | -1.3 | 0.050252 | -7.16 | 9.22E-19 | - | 402 | protein_coding | cortical cell-delineating protein-like [Aegilops tauschii subsp. tauschii] |
| TraesCS5A02G548600 | 1.55 | 4.62E-11 | -7.28 | 2.50E-202 | - | 1649 | protein_coding | predicted protein, partial [Hordeum vulgare subsp. vulgare] |
| TraesCS2D02G300100 | 1.45 | 0.002246 | -9.94 | 1.10E-21 | + | 1272 | protein_coding | loricrin-like [Aegilops tauschii subsp. tauschii] |
| TraesCS2A02G411600 | -1.6 | 0.075186 | -7.42 | 1.27E-08 | - | 381 | protein_coding | protease inhibitor-like protein [Triticum aestivum] |
| TraesCS4D02G360000 | 1.47 | 0.001995 | -8.69 | 2.21E-12 | + | 426 | protein_coding | pEARLI1-like lipid transfer protein 1 [Aegilops tauschii subsp. tauschii] |
| TraesCS2B02G126300 | -1.4 | 0.141914 | -7.97 | 3.46E-05 | - | 1254 | protein_coding | peroxidase 70 [Oryza sativa Japonica Group] |
| TraesCS1D02G331800 | -1.6 | 0.084268 | -7.91 | 1.29E-05 | - | 351 | protein_coding | cortical cell-delineating protein-like [Aegilops tauschii subsp. tauschii] |
| TraesCSU02G207700 | -0.7 | 0.816299 | 7.68 | NA | + | 1492 | protein_coding | Ananain [Triticum urartu] |
| TraesCS1D02G331900 | -1.2 | 0.185311 | -9.92 | 4.33E-09 | - | 441 | protein_coding | cortical cell-delineating protein-like [Aegilops tauschii subsp. tauschii] |
| TraesCS7B02G317800 | -2.4 | 3.85E-11 | -7.24 | 4.86E-18 | + | 384 | protein_coding | lipid transfer protein EARLI 1-like [Aegilops tauschii subsp. tauschii] |
| TraesCS7A02G417700 | -4.4 | 2.38E-52 | -8.19 | 2.90E-10 | + | 384 | protein_coding | 14 kDa proline-rich protein DC2.15 [Triticum urartu] |
| TraesCS1B02G135800 | -3.7 | 0.004521 | 8.44 | NA | - | 719 | protein_coding | metallothioneine type1, partial [Hordeum vulgare subsp. vulgare] |
| TraesCS7B02G298400 | -2.5 | 0.003274 | -9.62 | 4.47E-08 | + | 1266 | protein_coding | peroxidase 2-like [Aegilops tauschii subsp. tauschii] |
| TraesCS2A02G301500 | -0.8 | 0.17673 | -8.08 | 3.16E-10 | + | 1132 | protein_coding | loricrin-like [Aegilops tauschii subsp. tauschii] |
| TraesCS1D02G213400 | -1.2 | 0.183759 | -9.6 | 2.94E-09 | + | 345 | protein_coding | cortical cell-delineating protein-like [Aegilops tauschii subsp. tauschii] |
| TraesCS2B02G053200 | 6.38 | 2.45E-08 | -9.19 | 1.78E-09 | + | 1132 | protein_coding | xylanase inhibitor protein 1-like [Aegilops tauschii subsp. tauschii] |
| TraesCS1D02G040500 | NA | NA | 25.8 | NA | + | 1557 | protein_coding | DNA-directed RNA polymerase V subunit 1-like isoform X2 [Aegilops tauschii subsp. tauschii] |
| TraesCS1D02G285900 | -1.7 | 0.00192 | -7.24 | 5.92E-07 | + | 828 | protein_coding | uncharacterized protein LOC109748351 [Aegilops tauschii subsp. tauschii] |
| TraesCS7B02G384900 | -1.6 | 0.197735 | -8.18 | 4.81E-05 | - | 399 | protein_coding | predicted protein [Hordeum vulgare subsp. vulgare] |
| TraesCS3D02G538500 | 0.37 | 0.301551 | -7.23 | 9.03E-09 | - | 1422 | protein_coding | pectin acetylesterase 5-like isoform X1 [Aegilops tauschii subsp. tauschii] |
| TraesCS7D02G464600 | -1 | 0.002711 | -7.96 | 2.11E-18 | - | 1352 | protein_coding | predicted protein [Hordeum vulgare subsp. vulgare] |
| TraesCS1D02G027300 | -0.9 | 0.018984 | -7.66 | 1.62E-19 | + | 300 | protein_coding | Subtilisin-chymotrypsin inhibitor-2A [Triticum urartu] |
| TraesCS4D02G310500 | 0.33 | 0.762039 | -7.82 | 5.71E-10 | + | 480 | protein_coding | cortical cell-delineating protein-like [Aegilops tauschii subsp. tauschii] |
| TraesCS7A02G477100 | -1.5 | 0.000783 | -9.98 | 4.68E-15 | - | 1353 | protein_coding | peroxidase 70-like [Aegilops tauschii subsp. tauschii] |
| TraesCS7A02G452900 | 0.02 | 0.917404 | -7.32 | 2.93E-30 | + | 1299 | protein_coding | peroxidase 39-like [Aegilops tauschii subsp. tauschii] |
| TraesCS7B02G384600 | -3.6 | 0.000191 | -8.15 | 3.60E-08 | - | 399 | protein_coding | predicted protein [Hordeum vulgare subsp. vulgare] |
| TraesCS4B02G313055 | 1.62 | NA | -8.33 | NA | - | 477 | protein_coding | cortical cell-delineating protein-like [Aegilops tauschii subsp. tauschii] |
| TraesCS7A02G396400 | -2 | 0.010874 | -7.55 | 2.70E-10 | + | 1247 | protein_coding | peroxidase 2-like [Aegilops tauschii subsp. tauschii] |
| TraesCS4B02G313300 | 1.3 | 0.615785 | -8.08 | NA | - | 426 | protein_coding | cortical cell-delineating protein-like [Aegilops tauschii subsp. tauschii] |
| TraesCS1D02G027400 | 1.41 | 0.006307 | -7.56 | 3.71E-11 | + | 237 | protein_coding | Subtilisin-chymotrypsin inhibitor-2A [Triticum urartu] |
| TraesCS3D02G467700 | 2.06 | 4.32E-06 | -11.1 | 4.96E-14 | + | 1307 | protein_coding | basic 7S globulin-like [Aegilops tauschii subsp. tauschii] |
| TraesCS7B02G273700 | -1.7 | 0.028726 | -7.58 | 2.35E-10 | + | 1563 | protein_coding | berberine bridge enzyme-like 27 [Aegilops tauschii subsp. tauschii] |
| novel.8984 | NA | NA | 8.73 | 4.41E-12 | + | 401 | - | -- |
| TraesCS1B02G330200 | -1.7 | 0.000631 | -8.23 | 4.90E-10 | - | 1441 | protein_coding | peroxidase 1-like [Aegilops tauschii subsp. tauschii] |
| TraesCS1B02G331200 | -2.2 | 0.015781 | -7.86 | 1.68E-08 | + | 1286 | protein_coding | peroxidase 1-like [Aegilops tauschii subsp. tauschii] |
| TraesCS7B02G298600 | -2.3 | 1.77E-06 | -7.72 | 6.86E-09 | + | 1313 | protein_coding | peroxidase 2-like [Aegilops tauschii subsp. tauschii] |
| TraesCS1D02G318000 | -2.3 | 1.91E-06 | -8.63 | 2.81E-11 | + | 1419 | protein_coding | peroxidase 1-like [Aegilops tauschii subsp. tauschii] |
| TraesCS2A02G267200 | -1.7 | 5.86E-08 | -7.25 | 5.42E-11 | - | 548 | protein_coding | hypothetical protein TRIUR3_15154 [Triticum urartu] |
| TraesCS1A02G329700 | -0.6 | 0.59623 | -9.72 | 2.03E-05 | - | 351 | protein_coding | Cortical cell-delineating protein [Triticum urartu] |
| TraesCS2B02G482300 | -0.9 | 0.564417 | -7.22 | 0.012699 | + | 324 | protein_coding | RecName: Full=Cold-regulated protein 2 |
| TraesCS2D02G107800 | 8.35 | 1.14E-05 | -9.72 | 6.98E-06 | - | 1256 | protein_coding | peroxidase 1-like [Aegilops tauschii subsp. tauschii] |
| TraesCS7D02G469425 | -1 | 0.006699 | -9.41 | 1.03E-13 | - | 324 | protein_coding | cortical cell-delineating protein-like [Aegilops tauschii subsp. tauschii] |
| TraesCSU02G047800 | 1.14 | 8.93E-05 | -10.3 | 1.30E-17 | - | 2025 | protein_coding | unnamed protein product [Triticum aestivum] |
| TraesCS6B02G280400 | -1.8 | 0.000106 | -7.41 | 2.21E-08 | + | 2675 | protein_coding | beta-D-xylosidase 3-like [Aegilops tauschii subsp. tauschii] |
| TraesCS2D02G108100 | 2.4 | 8.14E-06 | -10.4 | 5.40E-11 | - | 1272 | protein_coding | unnamed protein product [Triticum aestivum] |
| novel.13266 | -2.3 | 2.76E-14 | -8.34 | 4.98E-11 | - | 3879 | - | uncharacterized protein LOC109771772 [Aegilops tauschii subsp. tauschii] |
| TraesCS2A02G040500 | 8.29 | 2.13E-12 | -9.59 | 2.83E-13 | - | 906 | protein_coding | xylanase inhibitor protein 1-like [Aegilops tauschii subsp. tauschii] |
| TraesCS1B02G330500 | -2.2 | 8.24E-06 | -8.27 | 9.69E-10 | + | 1425 | protein_coding | peroxidase 1-like [Aegilops tauschii subsp. tauschii] |
| TraesCS3D02G318000 | -2 | 1.70E-06 | -8.29 | 2.41E-10 | - | 1296 | protein_coding | blue copper protein-like [Aegilops tauschii subsp. tauschii] |
| TraesCSU02G144300 | 4.54 | 0.000396 | -7.13 | 9.90E-05 | + | 390 | protein_coding | cortical cell-delineating protein-like [Aegilops tauschii subsp. tauschii] |
| TraesCS1D02G022000 | 1.71 | NA | -7.55 | NA | - | 967 | protein_coding | Bowman-Birk trypsin inhibitor-like protein [Triticum aestivum] |
| TraesCSU02G144600 | 2.83 | 7.63E-09 | -10.1 | 1.29E-15 | + | 465 | protein_coding | cortical cell-delineating protein-like [Aegilops tauschii subsp. tauschii] |
| TraesCS4A02G412900 | 3.65 | 0.016681 | -8.37 | 9.08E-05 | + | 426 | protein_coding | Cortical cell-delineating protein [Triticum urartu] |
| TraesCSU02G144500 | 1.23 | 0.050176 | -7.92 | 5.33E-11 | + | 432 | protein_coding | cortical cell-delineating protein-like [Aegilops tauschii subsp. tauschii] |
| TraesCS7D02G049400 | 1.31 | 1.74E-05 | -9.78 | 8.55E-16 | - | 1937 | protein_coding | protein NRT1/ PTR FAMILY 2.3-like [Aegilops tauschii subsp. tauschii] |
| TraesCS1A02G356100 | -1.5 | 0.003491 | -7.51 | 8.00E-09 | + | 1194 | protein_coding | peroxidase 1-like [Aegilops tauschii subsp. tauschii] |
| TraesCS4B02G368300 | 2.56 | 7.62E-07 | -9.9 | 1.31E-14 | - | 432 | protein_coding | predicted protein [Hordeum vulgare subsp. vulgare] |
| TraesCS7B02G298500 | -1.4 | 0.161929 | -8.31 | 8.13E-09 | + | 1221 | protein_coding | peroxidase 2-like [Aegilops tauschii subsp. tauschii] |
| TraesCS2D02G008100 | -1.3 | 0.010923 | -8.43 | 5.01E-10 | + | 1794 | protein_coding | unnamed protein product [Triticum aestivum] |
| TraesCS2D02G108300 | -1.4 | 0.000552 | -7.41 | 9.36E-09 | - | 1291 | protein_coding | unnamed protein product [Triticum aestivum] |
| TraesCS5A02G535500 | 2.96 | 5.77E-06 | -9.41 | 9.50E-13 | - | 507 | protein_coding | Cortical cell-delineating protein [Triticum urartu] |
| TraesCS7B02G010600 | -1.9 | 2.64E-05 | -7.97 | 2.78E-09 | + | 258 | protein_coding | uncharacterized protein LOC109785281 [Aegilops tauschii subsp. tauschii] |
| TraesCS2D02G039300 | 7.7 | 2.28E-10 | -7.06 | 5.07E-08 | + | 1201 | protein_coding | xylanase inhibitor protein 1-like [Aegilops tauschii subsp. tauschii] |
| TraesCS1A02G318000 | -2.9 | 6.28E-07 | -7.15 | 3.60E-06 | - | 1300 | protein_coding | peroxidase 1-like [Aegilops tauschii subsp. tauschii] |
| TraesCSU02G144800 | 5.84 | 6.94E-12 | -9.41 | 4.04E-13 | + | 519 | protein_coding | pEARLI1-like lipid transfer protein 2 [Aegilops tauschii subsp. tauschii] |
| TraesCS3A02G077100 | NA | NA | 7.41 | 4.33E-09 | + | 2245 | protein_coding | asparagine synthetase [Triticum turgidum subsp. durum] |
| TraesCS1A02G318200 | -2.8 | 3.39E-15 | -7.15 | 1.20E-07 | - | 1241 | protein_coding | peroxidase 1-like [Aegilops tauschii subsp. tauschii] |
| TraesCS4A02G409400 | -1.5 | 0.014473 | -7.09 | 5.76E-07 | - | 3492 | protein_coding | MDIS1-interacting receptor like kinase 1-like [Aegilops tauschii subsp. tauschii] |
| TraesCSU02G243200 | -2.7 | 2.42E-17 | -7.13 | 8.95E-08 | + | 754 | protein_coding | unnamed protein product [Triticum aestivum] |
| TraesCS7D02G539100 | -1.7 | 0.000133 | -7.96 | 3.00E-09 | + | 1344 | protein_coding | tricetin 3',4',5'-O-trimethyltransferase-like [Aegilops tauschii subsp. tauschii] |
| TraesCS4A02G339000 | 1.59 | 3.96E-05 | -9.3 | 3.20E-14 | - | 1876 | protein_coding | 4-hydroxyphenylacetaldehyde oxime monooxygenase-like [Aegilops tauschii subsp. tauschii] |
| TraesCS4B02G369000 | 4.52 | 0.000177 | -9.71 | 1.14E-09 | - | 390 | protein_coding | cortical cell-delineating protein-like [Aegilops tauschii subsp. tauschii] |
| TraesCS5A02G418600 | -0.4 | 0.565176 | -8.68 | 6.85E-06 | + | 609 | protein_coding | predicted protein [Hordeum vulgare subsp. vulgare] |
| TraesCS2D02G458700 | -4.4 | 0.138904 | 23.6 | NA | - | 651 | protein_coding | hypothetical protein OsI_31610 [Oryza sativa Indica Group] |
| TraesCS1A02G209900 | -0.4 | 0.363641 | -8.65 | 1.23E-11 | - | 339 | protein_coding | cortical cell-delineating protein-like [Aegilops tauschii subsp. tauschii] |
| TraesCS1D02G318600 | -2.7 | 3.05E-06 | -7.06 | 1.34E-06 | + | 1274 | protein_coding | peroxidase 1-like [Aegilops tauschii subsp. tauschii] |
| TraesCS3A02G324700 | -1.4 | 0.012416 | -8.01 | 1.52E-08 | - | 894 | protein_coding | unnamed protein product [Triticum aestivum] |
| TraesCS1B02G027800 | 0.21 | 0.681191 | -7.03 | 2.04E-09 | - | 616 | protein_coding | Bowman-Birk trypsin inhibitor-like protein [Triticum aestivum] |
| TraesCS7A02G054100 | 0.43 | 0.234405 | -8.96 | 2.89E-13 | - | 1974 | protein_coding | protein NRT1/ PTR FAMILY 2.3-like [Aegilops tauschii subsp. tauschii] |
| TraesCSU02G243695 | 4.76 | 0.000507 | -7.71 | 2.25E-06 | - | 347 | protein_coding | cortical cell-delineating protein-like [Aegilops tauschii subsp. tauschii] |
| novel.2861 | NA | NA | 23.5 | NA | + | 1363 | - | -- |
| TraesCS2A02G002900 | 2.81 | 9.88E-08 | -8.79 | 2.01E-12 | - | 1911 | protein_coding | Cytochrome P450 86B1 [Triticum urartu] |
| TraesCS2A02G108000 | -1 | 0.116679 | -8.16 | 2.21E-08 | - | 1261 | protein_coding | unnamed protein product [Triticum aestivum] |
| TraesCS4B02G313200 | 1.06 | 0.025997 | -8.1 | 1.13E-10 | + | 480 | protein_coding | cortical cell-delineating protein-like [Aegilops tauschii subsp. tauschii] |
| TraesCS1A02G129600 | 4.25 | 0.000124 | -7.1 | 3.97E-07 | + | 905 | protein_coding | bidirectional sugar transporter SWEET3a [Aegilops tauschii subsp. tauschii] |
| novel.1280 | -2.4 | 5.46E-06 | -7.15 | 8.06E-07 | - | 2191 | - | extensin-like [Aegilops tauschii subsp. tauschii] |
| TraesCS5D02G561700 | 0.7 | 0.122101 | -7.93 | 5.31E-10 | + | 1302 | protein_coding | aquaporin PIP2-2-like [Aegilops tauschii subsp. tauschii] |
| TraesCS7D02G410700 | -1.7 | 0.005296 | -7.53 | 2.99E-07 | + | 384 | protein_coding | lipid transfer protein EARLI 1-like [Aegilops tauschii subsp. tauschii] |
| TraesCS6B02G018700 | -0.3 | 0.432437 | -8.26 | 6.09E-11 | - | 1220 | protein_coding | extradiol ring-cleavage dioxygenase-like [Aegilops tauschii subsp. tauschii] |
| TraesCS2A02G108200 | -1.5 | 2.93E-06 | -7.56 | 6.68E-09 | - | 1328 | protein_coding | unnamed protein product [Triticum aestivum] |
| TraesCS4D02G208900 | 4.27 | 3.65E-05 | -7.12 | 9.72E-08 | + | 2126 | protein_coding | laccase-10-like [Aegilops tauschii subsp. tauschii] |
| novel.13303 | -1.8 | 0.017903 | -7.32 | 6.61E-06 | - | 754 | - | -- |
| TraesCS6A02G137200 | -2 | 0.000337 | -7.14 | 6.16E-07 | + | 845 | protein_coding | Early nodulin-like protein 2 [Triticum urartu] |
| TraesCS2B02G173200 | -1.8 | 0.001146 | -7.18 | 4.05E-07 | + | 1372 | protein_coding | peroxidase 2-like [Aegilops tauschii subsp. tauschii] |
| TraesCSU02G242200 | 3.57 | 7.38E-07 | -8 | 1.60E-09 | + | 390 | protein_coding | cortical cell-delineating protein-like [Aegilops tauschii subsp. tauschii] |
| TraesCS1D02G021900 | 2.26 | NA | -8.79 | NA | - | 378 | protein_coding | Bowman-Birk trypsin inhibitor-like protein [Triticum aestivum] |
| TraesCS1D02G050600 | 2.27 | 0.01591 | -8.76 | 6.75E-05 | + | 709 | protein_coding | Salt stress-induced protein [Dichanthelium oligosanthes] |
| TraesCS3A02G460900 | -1.9 | 0.00107 | -7.02 | 1.21E-06 | - | 1708 | protein_coding | probable leucine-rich repeat receptor-like protein kinase At5g49770 [Aegilops tauschii subsp. tauschii] |
| TraesCS7B02G002000 | -0 | 0.941853 | -8.09 | 2.61E-10 | - | 855 | protein_coding | aquaporin PIP2-2-like [Aegilops tauschii subsp. tauschii] |
| novel.890 | NA | NA | 22.9 | NA | + | 1511 | - | hypothetical protein TRIUR3_19550 [Triticum urartu] |
| TraesCS1D02G214500 | 3.97 | 0.04098 | -8.8 | 0.000808 | + | 672 | protein_coding | predicted protein [Hordeum vulgare subsp. vulgare] |
| TraesCS4D02G104000 | 5.19 | 1.93E-10 | -8.81 | 3.83E-12 | + | 528 | protein_coding | dirigent protein 21-like [Aegilops tauschii subsp. tauschii] |
| TraesCS5D02G004100 | 0.01 | 0.987064 | -7.11 | 4.42E-08 | + | 447 | protein_coding | puroindoline a [Triticum turgidum subsp. durum x Aegilops tauschii] |
| TraesCS7D02G020100 | 6.04 | 0.00022 | -7.41 | 0.002808 | - | 1173 | protein_coding | extensin-like isoform X1 [Aegilops tauschii subsp. tauschii] |
| TraesCS1D02G006600 | 3.65 | 5.74E-06 | -8.72 | 2.09E-11 | + | 990 | protein_coding | hypothetical protein TRIUR3_28534 [Triticum urartu] |
| TraesCS7B02G115300 | 4.78 | 1.06E-06 | -7.03 | 9.21E-08 | - | 674 | protein_coding | GASR7 [Triticum aestivum] |
| TraesCS2D02G152700 | -0.2 | 0.77316 | -7.75 | 9.40E-09 | - | 1540 | protein_coding | peroxidase 2-like [Aegilops tauschii subsp. tauschii] |
| TraesCS5B02G528700 | -0.4 | 0.470878 | -7.63 | 3.86E-08 | + | 1187 | protein_coding | expansin-A31-like [Aegilops tauschii subsp. tauschii] |
| TraesCS5A02G558000 | -0.4 | 0.333361 | -7.61 | 6.13E-09 | - | 1616 | protein_coding | probable receptor-like protein kinase At4g10390 [Aegilops tauschii subsp. tauschii] |
| TraesCS3D02G318600 | -1.1 | 0.774585 | 7.57 | NA | + | 1230 | protein_coding | salicylic acid-binding protein 2-like [Aegilops tauschii subsp. tauschii] |
| TraesCSU02G154100 | 5.45 | 0.007086 | -8.57 | 0.000202 | + | 390 | protein_coding | cortical cell-delineating protein-like [Aegilops tauschii subsp. tauschii] |
| TraesCS3A02G220600 | NA | NA | 22.6 | NA | - | 3394 | protein_coding | autophagy-related protein 7c [Triticum aestivum] |
| TraesCS4B02G367800 | 1.82 | 0.07225 | -8.2 | 1.50E-08 | - | 432 | protein_coding | cortical cell-delineating protein-like [Aegilops tauschii subsp. tauschii] |
| novel.3884 | -0.8 | 0.063772 | -7.29 | 7.32E-08 | + | 576 | - | predicted protein [Hordeum vulgare subsp. vulgare] |
| TraesCS3B02G261500 | NA | NA | 21 | NA | + | 2941 | protein_coding | hypothetical protein SORBI_3001G021400 [Sorghum bicolor] |
| TraesCS4B02G008900 | -3.4 | 0.134507 | 7.24 | NA | - | 587 | protein_coding | 40S ribosomal protein S25-2 [Aegilops tauschii subsp. tauschii] |
| TraesCS7D02G139500 | 0.94 | 0.175078 | -7.67 | 1.47E-08 | + | 1554 | protein_coding | uncharacterized protein LOC109732600 [Aegilops tauschii subsp. tauschii] |
| TraesCS3B02G256900 | -5 | 0.070515 | 21.4 | NA | - | 1298 | protein_coding | unnamed protein product [Triticum aestivum] |
| TraesCS2B02G569500 | -0.3 | 0.931748 | 7.16 | NA | + | 1021 | protein_coding | predicted protein [Hordeum vulgare subsp. vulgare] |
| TraesCS3B02G405900 | -0.2 | 0.79462 | -7.08 | 1.10E-06 | + | 1153 | protein_coding | unnamed protein product [Triticum aestivum] |
| novel.4897 | 2.44 | 0.374949 | -7.74 | 0.047605 | + | 1614 | - | predicted protein [Hordeum vulgare subsp. vulgare] |
| TraesCS5B02G080800 | NA | NA | 22 | NA | - | 2515 | protein_coding | uncharacterized protein LOC109769396 [Aegilops tauschii subsp. tauschii] |
| TraesCS2A02G002800 | 4.41 | 1.11E-06 | -7.67 | 3.85E-09 | + | 1978 | protein_coding | Cytochrome P450 86B1 [Triticum urartu] |
| TraesCS4B02G395200 | NA | NA | 7.48 | NA | + | 564 | protein_coding | uncharacterized protein LOC109753500 [Aegilops tauschii subsp. tauschii] |
| TraesCS3B02G193500 | NA | NA | 7.04 | NA | - | 1774 | protein_coding | unnamed protein product [Triticum aestivum] |
| TraesCS5D02G249900 | 1.28 | 0.167895 | -7.39 | 2.04E-06 | + | 300 | protein_coding | hypothetical protein TRIUR3_16342 [Triticum urartu] |
| TraesCS3D02G338600 | 0.94 | 0.160337 | -7.26 | 2.80E-07 | + | 1292 | protein_coding | expansin-A2 [Oryza sativa Japonica Group] |
| TraesCS5A02G243400 | 1.41 | 0.052286 | -7.42 | 6.90E-08 | + | 288 | protein_coding | hypothetical protein TRIUR3_16342 [Triticum urartu] |
| TraesCS3A02G439000 | NA | NA | 21.8 | NA | + | 1088 | protein_coding | unnamed protein product [Triticum aestivum] |
| TraesCS2D02G527700 | -3.3 | 0.369967 | 21.6 | NA | + | 1299 | protein_coding | nucleolar protein 58-like isoform X1 [Aegilops tauschii subsp. tauschii] |
| TraesCS1D02G050800 | 3.86 | 0.000346 | -7.61 | 7.24E-08 | + | 792 | protein_coding | LOW QUALITY PROTEIN: mannose/glucose-specific lectin [Setaria italica] |
| TraesCS1D02G050700 | 4.04 | 0.000471 | -7.53 | 2.65E-07 | + | 486 | protein_coding | LOW QUALITY PROTEIN: mannose/glucose-specific lectin [Setaria italica] |
| TraesCS6B02G142700 | NA | NA | 21.6 | NA | + | 1884 | protein_coding | purple acid phosphatase precursor [Zea mays] |
| TraesCS7A02G452400 | 1.36 | 0.05297 | -7.15 | 1.23E-07 | + | 1652 | protein_coding | probable glycosyltransferase 4 [Aegilops tauschii subsp. tauschii] |
| novel.5131 | -3.6 | 0.312129 | 21.4 | NA | - | 855 | - | -- |
| TraesCSU02G185500 | 6.08 | 1.33E-05 | -7.45 | 1.04E-07 | + | 390 | protein_coding | cortical cell-delineating protein-like [Aegilops tauschii subsp. tauschii] |
| novel.5317 | -2 | 0.614929 | 21.4 | NA | - | 601 | - | predicted protein [Hordeum vulgare subsp. vulgare] |
| TraesCS5A02G270300 | 6.96 | 3.72E-07 | -7.37 | 6.05E-08 | + | 2445 | protein_coding | G-type lectin S-receptor-like serine/threonine-protein kinase [Panicum miliaceum] |
| novel.13630 | 2.53 | 0.019834 | -7.04 | 5.23E-06 | - | 459 | - | -- |
| TraesCSU02G212400 | NA | NA | 7.16 | NA | - | 768 | protein_coding | mavicyanin-like [Aegilops tauschii subsp. tauschii] |
| TraesCS2B02G576600 | NA | NA | 7.14 | NA | + | 3288 | protein_coding | uncharacterized protein LOC109785922 isoform X1 [Aegilops tauschii subsp. tauschii] |
| novel.9482 | NA | NA | 7.07 | NA | - | 486 | - | -- |
