# Supplementary material for: Comparative transcriptomic and metabolic profiling provides insight into the mechanism by which the autophagy inhibitor 3-MA enhances salt stress sensitivity in wheat seedlings
Source: BMC Plant Biol. 2021 Dec 6;21:577. doi: 10.1186/s12870-021-03351-5 (PMC8647401; doi:10.1186/s12870-021-03351-5)
Supplement: Supplementary file 13 — Additional file 13: Supplementary Table 10. Genes that were differentially expressed in wheat leaves between NaCl and 3-MA+ NaCl samples. [file 12870_2021_3351_MOESM13_ESM.docx]

Supplementary table 10 Genes that were diﬀerentially expressed in wheat leaves between NaCl and 3-MA+ NaCl samples

| gene_id | TYvsCY_log2FoldChange | TYvsCY_pvalue | TMYvsTY_log2FoldChange | TMYvsTY_pvalue | gene_strand | gene_length | gene_biotype | nr annotation |
| --- | --- | --- | --- | --- | --- | --- | --- | --- |
| TraesCS4D02G238600 | -0.33 | 0.793123507 | 8.1 | 6.32E-16 | + | 765 | protein_coding | hemoglobin 1 [Triticum aestivum] |
| TraesCS7B02G105300 | 4.21 | 0.05237719 | 8.73 | 2.28E-81 | + | 777 | protein_coding | pathogenesis-related protein 1-17 [Triticum aestivum] |
| TraesCS4B02G237300 | -2.13 | 0.518046544 | 9.62 | 1.33E-06 | + | 774 | protein_coding | Non-symbiotic hemoglobin [Triticum urartu] |
| TraesCS1D02G266000 | 1.56 | 0.439428885 | 7.03 | 4.74E-09 | + | 744 | protein_coding | uncharacterized protein LOC109755131 [Aegilops tauschii subsp. tauschii] |
| TraesCS2D02G431500 | -0.48 | 0.84777124 | 8.3 | 1.24E-07 | + | 1281 | protein_coding | unnamed protein product [Triticum aestivum] |
| TraesCS5D02G507800 | -2.76 | 0.031360991 | 7.82 | 9.74E-10 | - | 853 | protein_coding | cell number regulator 10-like [Aegilops tauschii subsp. tauschii] |
| TraesCS7B02G253600 | -1.24 | 0.62910029 | 11.2 | 6.62E-07 | - | 1735 | protein_coding | probable gamma-aminobutyrate transaminase 4 [Oryza sativa Japonica Group] |
| TraesCS4D02G200700 | -0.78 | 0.493332358 | 8.42 | 5.43E-08 | + | 1104 | protein_coding | protein IN2-1-like isoform X2 [Aegilops tauschii subsp. tauschii] |
| TraesCS3A02G141100 | NA | NA | 7.18 | 7.92E-06 | + | 1354 | protein_coding | GDSL esterase/lipase At5g45910-like [Aegilops tauschii subsp. tauschii] |
| TraesCS5B02G128100 | -3.64 | 7.23E-23 | -8.3 | 1.23E-10 | + | 1527 | protein_coding | cytochrome P450 94C1-like [Aegilops tauschii subsp. tauschii] |
| TraesCS3B02G328700 | -3 | 0.000656891 | 7.48 | 3.09E-08 | - | 1640 | protein_coding | unnamed protein product [Triticum aestivum] |
| TraesCS7B02G107700 | -1.54 | 8.54E-05 | -7.1 | 2.37E-12 | + | 817 | protein_coding | protein TIFY 11e-like [Aegilops tauschii subsp. tauschii] |
| TraesCS4A02G061700 | -3.61 | 0.24731546 | 11.1 | 4.85E-08 | + | 824 | protein_coding | hemoglobin 1 [Triticum aestivum] |
| TraesCS7B02G107800 | -1.57 | 0.029069828 | -7.4 | 4.59E-09 | + | 690 | protein_coding | Protein TIFY 3A [Triticum urartu] |
| TraesCS1A02G303100 | 1.39 | 0.729973996 | 8.95 | 7.04E-07 | + | 2234 | protein_coding | cytochrome P450 714D1-like [Aegilops tauschii subsp. tauschii] |
| TraesCS1B02G389700 | -2.22 | 0.053723567 | -7.7 | 1.15E-06 | + | 1007 | protein_coding | ethylene responsive transcription factor 6 [Triticum turgidum subsp. durum] |
| TraesCS7B02G245000 | 1.02 | 0.751433152 | 7.48 | 6.32E-13 | - | 192 | protein_coding | hypothetical protein TRIUR3_01539 [Triticum urartu] |
| TraesCS4B02G199700 | -3.22 | 4.88E-05 | 9.3 | 5.73E-65 | + | 1047 | protein_coding | protein IN2-1-like isoform X2 [Aegilops tauschii subsp. tauschii] |
| TraesCS1A02G370600 | -2.49 | 0.019503633 | -8.2 | 4.94E-07 | + | 1257 | protein_coding | AP2 domain containing protein [Zea mays] |
| TraesCS7B02G108000 | -1.39 | 0.000871812 | -7.5 | 9.13E-10 | + | 675 | protein_coding | predicted protein [Hordeum vulgare subsp. vulgare] |
| TraesCS4A02G104300 | -2.62 | 0.002340472 | 8.56 | 3.86E-23 | - | 1226 | protein_coding | Protein IN2-1 [Triticum urartu] |
| TraesCS3B02G549000 | NA | NA | 8.94 | 1.68E-11 | - | 2116 | protein_coding | hypothetical protein OsI_38200 [Oryza sativa Indica Group] |
| TraesCS7D02G523800 | NA | NA | 10.7 | 1.44E-07 | + | 2125 | protein_coding | alpha carbonic anhydrase 7-like [Aegilops tauschii subsp. tauschii] |
| TraesCS1D02G376500 | -2.31 | NA | -8.4 | NA | + | 1268 | protein_coding | ethylene-responsive transcription factor ERF109-like [Aegilops tauschii subsp. tauschii] |
| novel.3130 | 0.82 | 0.380881154 | 7.95 | 4.76E-24 | - | 7163 | - | OSJNBb0034I13.10 [Oryza sativa Japonica Group] |
| TraesCS1A02G186400 | -2.28 | 0.012004222 | 8.37 | 4.07E-22 | + | 1025 | protein_coding | putative glutathione S-transferase GSTU6 [Triticum urartu] |
| TraesCS3A02G212300 | -2.36 | 0.557677264 | 8.41 | 2.97E-09 | + | 1982 | protein_coding | PREDICTED: aldehyde dehydrogenase family 2 member C4-like [Oryza brachyantha] |
| TraesCS3D02G049700 | -3.82 | 0.287958385 | 8.37 | 2.34E-09 | + | 1998 | protein_coding | LEAF RUST 10 DISEASE-RESISTANCE LOCUS RECEPTOR-LIKE PROTEIN KINASE-like 2.4 [Aegilops tauschii subsp. tauschii] |
| TraesCS6A02G297500 | -2.38 | 0.319685545 | 7.34 | 3.26E-10 | - | 884 | protein_coding | blue copper protein-like [Aegilops tauschii subsp. tauschii] |
| TraesCS7B02G108300 | -2.62 | 4.55E-15 | -7 | 6.78E-17 | + | 821 | protein_coding | protein TIFY 11e-like [Aegilops tauschii subsp. tauschii] |
| TraesCS7B02G150000 | -0.72 | 0.241430036 | -7.3 | 4.51E-07 | + | 1309 | protein_coding | RING-H2 finger protein ATL16-like [Aegilops tauschii subsp. tauschii] |
| TraesCS5B02G148300 | -0.01 | 0.998095561 | 10.2 | 4.15E-18 | + | 1664 | protein_coding | UDP-glycosyltransferase [Triticum aestivum] |
| TraesCS5A02G149600 | 0.45 | 0.910917883 | 10.9 | 3.26E-15 | + | 2318 | protein_coding | UDP-glycosyltransferase 74E2-like [Aegilops tauschii subsp. tauschii] |
| TraesCS6D02G302300 | -0.87 | 0.285645004 | 8.04 | 5.41E-55 | - | 1508 | protein_coding | RecName: Full=Putative 12-oxophytodienoate reductase 11; AltName: Full=OPDA-reductase 11; Short=OsOPR11 |
| TraesCS2B02G454500 | 1.98 | 0.620500181 | 10.8 | 4.31E-13 | + | 1499 | protein_coding | putative xyloglucan endotransglucosylase/hydrolase protein 13 [Aegilops tauschii subsp. tauschii] |
| TraesCS7D02G204700 | -2.83 | 0.000342056 | -7.1 | 8.97E-09 | + | 742 | protein_coding | protein TIFY 11e-like [Aegilops tauschii subsp. tauschii] |
| TraesCS5D02G360600 | 2.39 | 0.547020803 | 10.6 | 1.69E-11 | - | 1005 | protein_coding | 11S globulin seed storage protein 2-like [Aegilops tauschii subsp. tauschii] |
| TraesCS3D02G039400 | NA | NA | 7.92 | 0.00090083 | + | 1351 | protein_coding | uncharacterized protein LOC109772661 [Aegilops tauschii subsp. tauschii] |
| TraesCS6A02G181000 | 1.02 | 0.579903776 | 9.24 | 4.66E-12 | + | 1652 | protein_coding | Premnaspirodiene oxygenase [Triticum urartu] |
| TraesCS5D02G559000 | -2.73 | 0.113273452 | -8.7 | NA | + | 1992 | protein_coding | uncharacterized protein LOC109771141 [Aegilops tauschii subsp. tauschii] |
| TraesCS5A02G017900 | -1.3 | 0.512559309 | 7.1 | 0.000169036 | - | 942 | protein_coding | thaumatin-like protein [Triticum aestivum] |
| novel.9925 | -3.44 | 0.095648989 | 8.16 | 8.45E-11 | - | 1002 | - | 12-oxophytodienoic acid reductase 2 [Hordeum vulgare subsp. vulgare] |
| TraesCS4B02G086100 | -0.8 | 0.682651856 | 9.18 | 5.27E-07 | + | 1355 | protein_coding | 2-alkenal reductase (NADP(+)-dependent)-like [Aegilops tauschii subsp. tauschii] |
| TraesCS1D02G302500 | NA | NA | 8.99 | 4.05E-05 | - | 2438 | protein_coding | cytochrome P450 714D1-like [Aegilops tauschii subsp. tauschii] |
| TraesCS3D02G514300 | -0.45 | 0.912694072 | 12.4 | 8.10E-17 | - | 1447 | protein_coding | 3-oxo-Delta(4,5)-steroid 5-beta-reductase-like [Aegilops tauschii subsp. tauschii] |
| novel.5891 | 2 | 0.616827496 | 8.71 | 1.39E-20 | - | 2166 | - | glutathione transferase [Triticum aestivum] |
| novel.12621 | -1.89 | 0.02669583 | -7.1 | 2.87E-07 | - | 4559 | - | alpha-terpineol synthase, chloroplastic-like [Aegilops tauschii subsp. tauschii] |
| TraesCS3D02G104400 | -1.9 | 0.082345582 | 7.7 | 2.66E-15 | - | 1160 | protein_coding | probable NADPH:quinone oxidoreductase 1 [Aegilops tauschii subsp. tauschii] |
| novel.6128 | NA | NA | 8.03 | 1.80E-08 | + | 475 | - | hypothetical protein TRIUR3_04340 [Triticum urartu] |
| TraesCS1A02G370400 | -2.55 | 0.067387376 | -8.2 | 0.001994774 | + | 1341 | protein_coding | ethylene-responsive transcription factor ERF109-like [Aegilops tauschii subsp. tauschii] |
| TraesCS4B02G366800 | NA | NA | 7.72 | 1.15E-07 | + | 1518 | protein_coding | flavin-containing monooxygenase FMO GS-OX-like 4 isoform X1 [Aegilops tauschii subsp. tauschii] |
| TraesCS3D02G514400 | -2.7 | 0.000636711 | 7.25 | 4.11E-07 | + | 1420 | protein_coding | 3-oxo-Delta(4,5)-steroid 5-beta-reductase-like [Aegilops tauschii subsp. tauschii] |
| TraesCS5A02G238400 | -1.65 | 0.371624084 | -7.4 | 0.016252517 | + | 1179 | protein_coding | ethylene-responsive transcription factor ERF109-like [Aegilops tauschii subsp. tauschii] |
| TraesCS3B02G335300 | NA | NA | 8.13 | 0.037418612 | + | 2346 | protein_coding | predicted protein, partial [Hordeum vulgare subsp. vulgare] |
| TraesCS1D02G190200 | -0.67 | 0.532712788 | 8.47 | 6.26E-43 | - | 997 | protein_coding | glutathione S-transferase U17-like [Aegilops tauschii subsp. tauschii] |
| TraesCS3D02G483000 | 3.41 | 0.249382508 | 9.02 | 1.42E-15 | - | 1273 | protein_coding | glutathione S-transferase U8-like [Aegilops tauschii subsp. tauschii] |
| TraesCS6D02G127700 | -3.94 | 0.000977557 | 9.65 | 7.34E-23 | - | 1102 | protein_coding | glutathione S-transferase 1 [Aegilops tauschii] |
| TraesCS2D02G030500 | -6.34 | 0.010984565 | -7.1 | 0.070870673 | - | 1399 | protein_coding | unnamed protein product [Triticum aestivum] |
| TraesCS7D02G466900 | 1.98 | 0.620500181 | 8.86 | 6.60E-23 | + | 1465 | protein_coding | ureide permease 1-like [Aegilops tauschii subsp. tauschii] |
| TraesCS6D02G127800 | 0.25 | 0.913895135 | 7.97 | 3.47E-05 | - | 1017 | protein_coding | glutathione-S-transferase 2 [Aegilops tauschii] |
| TraesCS7B02G264200 | -4.35 | 0.185555889 | 7.61 | 7.70E-15 | - | 2736 | protein_coding | beta-galactosidase precursor [Zea mays] |
| novel.3946 | NA | NA | 9.43 | 1.03E-06 | + | 4811 | - | Os08g0544300 [Oryza sativa Japonica Group] |
| TraesCS3B02G290200 | -4.29 | 0.10920209 | 12.8 | 8.12E-16 | - | 1737 | protein_coding | unnamed protein product [Triticum aestivum] |
| TraesCS7B02G290200 | 0.43 | 0.914750567 | 9.08 | 1.44E-15 | - | 707 | protein_coding | 60S acidic ribosomal protein P2A-like [Aegilops tauschii subsp. tauschii] |
| TraesCS6A02G260900 | -0.38 | 0.907223694 | 8.69 | 3.77E-08 | - | 1495 | protein_coding | Anthranilate N-benzoyltransferase protein 1 [Triticum urartu] |
| TraesCS1D02G073000 | -0.45 | 0.912694072 | 8.58 | 1.73E-10 | + | 1748 | protein_coding | nicotianamine aminotransferase A-like [Aegilops tauschii subsp. tauschii] |
| TraesCS2D02G442700 | -4.98 | 0.000275921 | 8.24 | 0.000571608 | - | 2160 | protein_coding | protein NRT1/ PTR FAMILY 8.3-like [Aegilops tauschii subsp. tauschii] |
| TraesCS3A02G062200 | NA | NA | 12.6 | 1.57E-15 | + | 507 | protein_coding | Cytochrome P450 71C2 [Triticum urartu] |
| TraesCS3B02G220700 | 2.04 | 0.461114048 | 7.87 | 1.18E-09 | - | 864 | protein_coding | eukaryotic translation initiation factor 6-2 [Arachis duranensis] |
| TraesCS7B02G400500 | -5.22 | 0.005458026 | 12.1 | 3.75E-14 | - | 1716 | protein_coding | protein DETOXIFICATION 16-like isoform X2 [Aegilops tauschii subsp. tauschii] |
| TraesCS6B02G011500 | -3.17 | 0.195276497 | 9.1 | 9.98E-14 | - | 905 | protein_coding | thiosulfate sulfurtransferase 16, chloroplastic-like [Aegilops tauschii subsp. tauschii] |
| TraesCS7D02G484500 | NA | NA | 11.4 | 1.37E-12 | + | 1634 | protein_coding | predicted protein [Hordeum vulgare subsp. vulgare] |
| TraesCS1B02G394700 | NA | NA | 7.53 | 2.33E-08 | + | 734 | protein_coding | non-specific lipid-transfer protein 2-like [Aegilops tauschii subsp. tauschii] |
| TraesCS6B02G052200 | -4.4 | 0.16500359 | 10.1 | 1.50E-07 | + | 1781 | protein_coding | indole-2-monooxygenase-like [Aegilops tauschii subsp. tauschii] |
| TraesCS7D02G466800 | 2.4 | 0.527902022 | 8.76 | 2.90E-30 | - | 1619 | protein_coding | ureide permease 1-like [Aegilops tauschii subsp. tauschii] |
| TraesCS6A02G139100 | 0.16 | 0.947801055 | 7.96 | 7.45E-05 | - | 1083 | protein_coding | glutathione-S-transferase 2 [Aegilops tauschii] |
| TraesCS7D02G028100 | NA | NA | 8.7 | 4.63E-11 | - | 1815 | protein_coding | asparagine--tRNA ligase, cytoplasmic 1-like [Aegilops tauschii subsp. tauschii] |
| TraesCS2D02G582000 | -4.93 | 0.085860357 | 10.8 | 5.12E-06 | - | 1650 | protein_coding | tryptamine benzoyltransferase 1-like [Aegilops tauschii subsp. tauschii] |
| TraesCS7B02G382000 | NA | NA | 9.31 | 2.59E-14 | + | 1759 | protein_coding | ureide permease 1-like [Aegilops tauschii subsp. tauschii] |
| TraesCS6A02G331300 | -1.09 | 0.604089145 | 8.42 | 1.23E-10 | + | 1765 | protein_coding | 7-deoxyloganetin glucosyltransferase-like [Aegilops tauschii subsp. tauschii] |
| TraesCS2D02G072600 | 2 | 0.319181275 | 7.04 | 2.80E-25 | + | 1807 | protein_coding | UDP-glycosyltransferase 74F2-like [Aegilops tauschii subsp. tauschii] |
| TraesCS3A02G260100 | -2.72 | 0.099639681 | 7.63 | 5.63E-06 | - | 1256 | protein_coding | endochitinase [Triticum aestivum] |
| TraesCS2A02G534600 | 2.71 | 0.493046154 | 8.59 | 1.54E-06 | + | 1837 | protein_coding | isoflavone 2'-hydroxylase-like [Aegilops tauschii subsp. tauschii] |
| novel.9926 | -2.3 | 0.095829112 | 7.18 | 4.32E-06 | - | 384 | - | putative 12-oxophytodienoate reductase 11 [Aegilops tauschii subsp. tauschii] |
| TraesCS5B02G021800 | NA | NA | 8.98 | 2.05E-11 | - | 998 | protein_coding | glutathione transferase F4 [Triticum aestivum] |
| TraesCS6D02G241700 | NA | NA | 9.56 | 3.73E-13 | - | 1626 | protein_coding | putrescine hydroxycinnamoyltransferase 1-like [Aegilops tauschii subsp. tauschii] |
| TraesCS7B02G131600 | -3.72 | 2.98E-40 | -8.9 | 2.03E-12 | + | 243 | protein_coding | MYB-related protein [Aegilops speltoides] |
| TraesCS7A02G497300 | NA | NA | 10.1 | 5.56E-07 | + | 1615 | protein_coding | protein DETOXIFICATION 16-like isoform X2 [Aegilops tauschii subsp. tauschii] |
| TraesCS6B02G167600 | -3.62 | 0.252515195 | 11.3 | 1.65E-09 | - | 917 | protein_coding | probable glutathione S-transferase GSTU6 [Aegilops tauschii subsp. tauschii] |
| TraesCS4B02G188800 | -5.19 | 0.006953809 | 7.84 | 8.05E-08 | - | 1208 | protein_coding | sex determination protein tasselseed-2-like [Aegilops tauschii subsp. tauschii] |
| TraesCS3D02G094300 | NA | NA | 9.74 | 4.36E-08 | + | 671 | protein_coding | uncharacterized protein LOC109769231 [Aegilops tauschii subsp. tauschii] |
| TraesCS3B02G536100 | -1.43 | 0.492200838 | 8.76 | 2.10E-08 | - | 972 | protein_coding | glutathione S-transferase U8-like [Aegilops tauschii subsp. tauschii] |
| TraesCS7D02G328700 | -4.13 | 0.083971815 | 7.84 | 2.29E-07 | + | 878 | protein_coding | uncharacterized protein LOC109748612 [Aegilops tauschii subsp. tauschii] |
| TraesCS4A02G454800 | -4.74 | 0.051543305 | 11.3 | 4.13E-19 | + | 1060 | protein_coding | probable glutathione S-transferase GSTU6 [Aegilops tauschii subsp. tauschii] |
| novel.12163 | 1.94 | 0.402151736 | 7.52 | 4.24E-09 | + | 2047 | - | hypothetical protein TRIUR3_06504 [Triticum urartu] |
| TraesCS1D02G338500 | -1.4 | 0.720124831 | 9.3 | 1.60E-08 | + | 933 | protein_coding | 28 kDa heat- and acid-stable phosphoprotein-like [Aegilops tauschii subsp. tauschii] |
| novel.1377 | NA | NA | 11.7 | 2.71E-11 | + | 3266 | - | G-type lectin S-receptor-like serine/threonine-protein kinase At1g34300 [Aegilops tauschii subsp. tauschii] |
| TraesCS1A02G435100 | 1.02 | 0.666916773 | 7.1 | 8.00E-07 | + | 792 | protein_coding | Calmodulin-like protein 5 [Triticum urartu] |
| TraesCS5D02G312100 | 0.14 | 0.942251661 | 8.16 | 5.84E-14 | + | 1645 | protein_coding | UDP-glycosyltransferase 74E1-like [Aegilops tauschii subsp. tauschii] |
| TraesCS7B02G105100 | -0.96 | 0.777339736 | 8.46 | 2.53E-14 | + | 866 | protein_coding | pathogenesis-related protein 1-18 [Triticum aestivum] |
| TraesCS5B02G267800 | 0.05 | 0.987723161 | 8.42 | 1.83E-08 | + | 1557 | protein_coding | protein PIN-LIKES 3-like [Aegilops tauschii subsp. tauschii] |
| novel.13733 | -1.1 | 0.449653332 | 7.49 | 1.21E-11 | - | 525 | - | -- |
| TraesCS2B02G187200 | -2.24 | 0.580099235 | 7.62 | 4.77E-07 | - | 1382 | protein_coding | probable trehalose-phosphate phosphatase 3 [Aegilops tauschii subsp. tauschii] |
| TraesCS2B02G480400 | NA | NA | 9.09 | 1.05E-06 | - | 429 | protein_coding | ubiquitin [Setaria italica] |
| TraesCS7D02G168600 | -0.7 | 0.652976613 | 7.45 | 5.31E-20 | - | 882 | protein_coding | cyclic phosphodiesterase-like [Aegilops tauschii subsp. tauschii] |
| TraesCS6D02G332900 | 2.75 | 0.487900855 | 7.4 | 1.27E-06 | + | 1100 | protein_coding | dehydrin DHN4-like [Aegilops tauschii subsp. tauschii] |
| TraesCS7A02G479700 | -0.47 | 0.897361832 | 8.53 | 2.22E-14 | + | 1623 | protein_coding | ureide permease 1-like [Aegilops tauschii subsp. tauschii] |
| TraesCS7D02G410000 | -3.56 | 0.135982518 | 9.27 | 1.50E-12 | - | 483 | protein_coding | uncharacterized protein LOC109747823 [Aegilops tauschii subsp. tauschii] |
| TraesCS3B02G050300 | NA | NA | 8.89 | 8.98E-06 | + | 1685 | protein_coding | unnamed protein product [Triticum aestivum] |
| TraesCS1D02G256500 | -0.87 | 0.711389705 | 8.09 | 3.65E-16 | - | 1734 | protein_coding | AAA-ATPase ASD, mitochondrial-like [Aegilops tauschii subsp. tauschii] |
| novel.1311 | -0.38 | 0.875214177 | 7.86 | 4.70E-11 | - | 1082 | - | -- |
| TraesCS4A02G229900 | -2.86 | 0.179383822 | 9.45 | 1.73E-17 | - | 1056 | protein_coding | 2-alkenal reductase (NADP(+)-dependent)-like [Aegilops tauschii subsp. tauschii] |
| TraesCS6D02G379100 | -0.45 | 0.912694072 | 9.29 | 5.71E-09 | + | 892 | protein_coding | cytokinesis protein sepA-like [Aegilops tauschii subsp. tauschii] |
| TraesCS4D02G031900 | -2.24 | 0.580099235 | 11.2 | 2.56E-13 | + | 675 | protein_coding | oxalate oxidase GF-2.8-like [Aegilops tauschii subsp. tauschii] |
| TraesCS5A02G439700 | NA | NA | 8.79 | 0.001265541 | + | 783 | protein_coding | pathogenesis-related protein 1-9 [Triticum aestivum] |
| TraesCS1D02G236900 | NA | NA | 8.29 | 6.49E-10 | + | 1830 | protein_coding | threonine--tRNA ligase, mitochondrial 1-like [Panicum miliaceum] |
| TraesCS3D02G084700 | NA | NA | 11.2 | 9.98E-11 | + | 1637 | protein_coding | cytochrome P450 704C1-like [Aegilops tauschii subsp. tauschii] |
| TraesCS7B02G253700 | NA | NA | 9.2 | 0.000323426 | + | 1820 | protein_coding | putative Cytochrome P450 [Oryza sativa Japonica Group] |
| TraesCS5B02G444500 | 1.32 | 0.006158434 | -7.8 | 1.25E-28 | - | 1385 | protein_coding | tryptophan synthase alpha chain-like isoform X2 [Aegilops tauschii subsp. tauschii] |
| TraesCS6B02G108100 | 0.48 | NA | -22 | NA | + | 1934 | protein_coding | predicted protein [Hordeum vulgare subsp. vulgare] |
| TraesCS6A02G139000 | NA | NA | 9.23 | 4.78E-05 | - | 904 | protein_coding | glutathione-S-transferase 28e45 [Triticum aestivum] |
| TraesCS4A02G330100 | NA | NA | 7.53 | 1.28E-07 | - | 1182 | protein_coding | receptor kinase-like protein Xa21 [Aegilops tauschii subsp. tauschii] |
| TraesCS4D02G032100 | 1.99 | 0.6192703 | 8.63 | 9.13E-12 | + | 552 | protein_coding | oxalate oxidase 2-like [Aegilops tauschii subsp. tauschii] |
| TraesCS2A02G474500 | NA | NA | 8.72 | 2.96E-10 | - | 868 | protein_coding | unnamed protein product [Triticum aestivum] |
| TraesCS7A02G353400 | -2.28 | 0.573196153 | 7.01 | 0.072909453 | - | 1229 | protein_coding | predicted protein [Hordeum vulgare subsp. vulgare] |
| TraesCS7A02G132900 | -0.56 | 0.307353427 | -7.6 | 9.67E-09 | - | 1156 | protein_coding | uncharacterized protein LOC109766650 [Aegilops tauschii subsp. tauschii] |
| TraesCS3B02G109700 | NA | NA | 9.46 | 1.12E-09 | + | 608 | protein_coding | uncharacterized protein LOC109769231 [Aegilops tauschii subsp. tauschii] |
| TraesCS5B02G327800 | NA | NA | 8.3 | 3.37E-09 | - | 1929 | protein_coding | aspartate--tRNA ligase 2, cytoplasmic-like [Aegilops tauschii subsp. tauschii] |
| TraesCS1A02G394100 | NA | NA | 8.64 | 1.58E-10 | - | 804 | protein_coding | probable calcium-binding protein CML30 [Aegilops tauschii subsp. tauschii] |
| TraesCS6B02G167400 | NA | NA | 10 | 1.51E-06 | - | 672 | protein_coding | glutathione-S-transferase 2 [Aegilops tauschii] |
| TraesCS6D02G284200 | NA | NA | 10.8 | 1.55E-06 | + | 2052 | protein_coding | putative laccase-9 [Aegilops tauschii subsp. tauschii] |
| TraesCS3D02G429900 | -4.98 | 0.013599054 | 9.78 | 1.93E-08 | + | 1033 | protein_coding | predicted protein [Hordeum vulgare subsp. vulgare] |
| TraesCS2D02G165100 | NA | NA | 7.71 | 1.44E-07 | + | 1769 | protein_coding | phloretin 2'-O-glucosyltransferase-like [Aegilops tauschii subsp. tauschii] |
| TraesCS3B02G597000 | NA | NA | 10.8 | 1.60E-12 | + | 1968 | protein_coding | unnamed protein product [Triticum aestivum] |
| TraesCS4A02G181900 | -2.24 | 0.580099235 | 10.4 | 4.92E-16 | + | 657 | protein_coding | oxalate oxidase 1-like [Aegilops tauschii subsp. tauschii] |
| TraesCS3B02G150500 | -2.28 | 0.573196153 | 10 | 2.24E-05 | + | 1251 | protein_coding | unnamed protein product [Triticum aestivum] |
| TraesCSU02G026200 | NA | NA | 8.09 | 3.57E-08 | - | 1166 | protein_coding | xylanase inhibitor protein 1-like [Aegilops tauschii subsp. tauschii] |
| TraesCS2D02G500300 | -3.55 | 0.351769028 | 7.11 | 2.05E-05 | - | 1421 | protein_coding | anthocyanidin reductase ((2S)-flavan-3-ol-forming)-like isoform X1 [Aegilops tauschii subsp. tauschii] |
| TraesCS1B02G194600 | NA | NA | 7.13 | 2.19E-05 | - | 345 | protein_coding | predicted protein [Hordeum vulgare subsp. vulgare] |
| TraesCS5B02G014700 | -3.06 | 0.001736541 | -7.1 | 2.16E-06 | + | 1879 | protein_coding | zingiberene synthase-like [Aegilops tauschii subsp. tauschii] |
| novel.2216 | -4.82 | 0.100240921 | 7.73 | 2.93E-08 | - | 1586 | - | uncharacterized protein LOC109734236 [Aegilops tauschii subsp. tauschii] |
| TraesCS2A02G203200 | 2.71 | 0.493046154 | 7.32 | 0.000270059 | - | 2124 | protein_coding | internal alternative NAD(P)H-ubiquinone oxidoreductase A1, mitochondrial-like [Aegilops tauschii subsp. tauschii] |
| TraesCS4B02G225200 | -4.23 | 0.197268413 | 8.28 | 6.72E-09 | - | 1362 | protein_coding | glutathione-S-transferase Cla47 [Triticum aestivum] |
| TraesCS4A02G006600 | NA | NA | 8.28 | 0.034148008 | - | 1763 | protein_coding | probable flavin-containing monooxygenase 1 [Aegilops tauschii subsp. tauschii] |
| TraesCS2A02G345500 | 0.35 | 0.889623776 | 7.31 | 3.63E-08 | - | 1906 | protein_coding | predicted protein [Hordeum vulgare subsp. vulgare] |
| TraesCS5A02G016700 | -2.79 | 0.020679239 | -8.1 | 0.000197856 | + | 951 | protein_coding | zingiberene synthase-like [Aegilops tauschii subsp. tauschii] |
| TraesCS3A02G508600 | 2.72 | 0.440550927 | 7.22 | 4.86E-18 | + | 1692 | protein_coding | 3-oxo-Delta(4,5)-steroid 5-beta-reductase-like [Aegilops tauschii subsp. tauschii] |
| TraesCS3A02G082900 | NA | NA | 10.4 | 2.08E-05 | - | 1800 | protein_coding | cytochrome P450 704C1-like [Aegilops tauschii subsp. tauschii] |
| TraesCS2A02G318800 | -4.78 | 0.0251074 | 7.06 | 0.07085304 | + | 1012 | protein_coding | hypothetical protein TRIUR3_18331 [Triticum urartu] |
| TraesCS3D02G478200 | -4.69 | 0.117666567 | 8.47 | 0.001533159 | + | 1253 | protein_coding | predicted protein [Hordeum vulgare subsp. vulgare] |
| TraesCS5A02G353400 | NA | NA | 8.64 | 2.55E-06 | - | 1427 | protein_coding | 11S globulin seed storage protein 2-like [Aegilops tauschii subsp. tauschii] |
| TraesCS6B02G109400 | 0.51 | NA | -21 | NA | + | 1836 | protein_coding | stAR-related lipid transfer protein 7, mitochondrial-like [Aegilops tauschii subsp. tauschii] |
| TraesCS1D02G444200 | NA | NA | 9.14 | 1.62E-11 | + | 582 | protein_coding | calmodulin-like protein 3 [Aegilops tauschii subsp. tauschii] |
| TraesCS1D02G402400 | NA | NA | 8.2 | 0.000288975 | + | 558 | protein_coding | probable calcium-binding protein CML30 [Aegilops tauschii subsp. tauschii] |
| TraesCS4B02G133900 | -2.93 | 0.429489567 | 9.18 | 1.63E-11 | + | 1007 | protein_coding | uncharacterized protein LOC109779747 [Aegilops tauschii subsp. tauschii] |
| TraesCS7B02G370400 | NA | NA | 7.67 | 4.53E-09 | - | 420 | protein_coding | auxin-responsive protein SAUR71-like [Aegilops tauschii subsp. tauschii] |
| novel.2206 | NA | NA | 9.49 | 3.91E-07 | - | 2997 | - | hypothetical protein, partial [Triticum monococcum] |
| TraesCS7B02G418400 | -0.84 | NA | -7.6 | 0.051433823 | + | 1470 | protein_coding | WRKY transcription factor WRKY28-like [Aegilops tauschii subsp. tauschii] |
| novel.2398 | 0.75 | NA | -20 | NA | - | 4369 | - | uncharacterized protein LOC109770721 [Aegilops tauschii subsp. tauschii] |
| novel.9380 | 0.2 | NA | -7 | NA | - | 1385 | - | uncharacterized protein LOC109741435 isoform X2 [Aegilops tauschii subsp. tauschii] |
| TraesCS7B02G257300 | 1.59 | 0.004786652 | -8 | 2.37E-10 | + | 1653 | protein_coding | acyl transferase 10-like [Aegilops tauschii subsp. tauschii] |
| TraesCS5D02G403700 | -8.72 | 1.32E-06 | 7.2 | 0.00082429 | + | 1786 | protein_coding | UDP-glycosyltransferase 83A1-like [Aegilops tauschii subsp. tauschii] |
| TraesCS2B02G030500 | -1.01 | NA | -9 | 0.002629517 | - | 1778 | protein_coding | unnamed protein product [Triticum aestivum] |
| TraesCS3A02G279600 | NA | NA | 7.28 | 1.85E-07 | - | 1416 | protein_coding | Anthocyanidin 5,3-O-glucosyltransferase [Triticum urartu] |
| TraesCS3A02G061700 | NA | NA | 9.12 | 4.46E-13 | + | 1500 | protein_coding | unnamed protein product [Triticum aestivum] |
| TraesCS1B02G113600 | NA | NA | 9.96 | 4.37E-14 | + | 833 | protein_coding | glutathione S-transferase 4-like [Aegilops tauschii subsp. tauschii] |
| TraesCS6A02G305000 | -3.67 | 0.233960675 | 10.1 | 2.82E-06 | + | 1737 | protein_coding | putative laccase-9 [Aegilops tauschii subsp. tauschii] |
| TraesCS5D02G314100 | NA | NA | 9.82 | 7.52E-14 | + | 1422 | protein_coding | serine carboxypeptidase-like 2 [Aegilops tauschii subsp. tauschii] |
| TraesCS5B02G491800 | -3.64 | 0.331265633 | 7.83 | 6.22E-09 | + | 866 | protein_coding | actin-depolymerizing factor 3-like [Aegilops tauschii subsp. tauschii] |
| TraesCS7A02G021700 | 0.44 | NA | -21 | NA | - | 4219 | protein_coding | disease resistance protein RPP13-like [Aegilops tauschii subsp. tauschii] |
| TraesCS1B02G470900 | -1.28 | 0.750626217 | 8.15 | 9.30E-10 | + | 838 | protein_coding | calmodulin-like protein 7 [Aegilops tauschii subsp. tauschii] |
| TraesCS5B02G359300 | NA | NA | 8.09 | 0.038569365 | - | 2194 | protein_coding | benzoate--CoA ligase, peroxisomal-like [Aegilops tauschii subsp. tauschii] |
| TraesCS5A02G472300 | NA | NA | 9.89 | 8.87E-09 | + | 1956 | protein_coding | indole-2-monooxygenase-like isoform X1 [Aegilops tauschii subsp. tauschii] |
| novel.9517 | NA | NA | 9.99 | 5.90E-09 | + | 5166 | - | Os08g0389500 [Oryza sativa Japonica Group] |
| novel.9932 | -3.63 | 0.175124778 | 7.19 | 1.00E-06 | - | 524 | - | -- |
| TraesCS3B02G529400 | -2.27 | 0.497580071 | 8.11 | 0.000776592 | - | 1250 | protein_coding | glucan endo-1,3-beta-glucosidase GII-like [Aegilops tauschii subsp. tauschii] |
| TraesCS1A02G410400 | NA | NA | 7.16 | 7.41E-07 | - | 492 | protein_coding | uncharacterized protein LOC109736917 [Aegilops tauschii subsp. tauschii] |
| TraesCS5A02G467800 | NA | NA | 8.01 | 0.000502595 | + | 890 | protein_coding | probable calcium-binding protein CML18 [Aegilops tauschii subsp. tauschii] |
| TraesCS2D02G483300 | 2 | 0.616827496 | 7.11 | 1.30E-11 | + | 1891 | protein_coding | organic cation/carnitine transporter 4-like [Aegilops tauschii subsp. tauschii] |
| TraesCS5A02G016600 | -2.92 | 0.006354018 | -7.3 | 4.26E-06 | + | 1374 | protein_coding | (E)-beta-farnesene synthase [Triticum urartu] |
| TraesCS1D02G338000 | 0.02 | 0.976836213 | -9.2 | 1.11E-06 | - | 1323 | protein_coding | acetylserotonin O-methyltransferase 1-like isoform X1 [Aegilops tauschii subsp. tauschii] |
| TraesCS4A02G279300 | 1.41 | 0.726267558 | 7.42 | 8.54E-11 | + | 657 | protein_coding | oxalate oxidase 1-like [Aegilops tauschii subsp. tauschii] |
| TraesCS6B02G288300 | NA | NA | 7.37 | 1.09E-06 | - | 1314 | protein_coding | putrescine hydroxycinnamoyltransferase 1-like [Aegilops tauschii subsp. tauschii] |
| novel.12303 | -0.62 | 0.867568185 | -21 | NA | - | 2159 | - | uncharacterized protein LOC109733110 [Aegilops tauschii subsp. tauschii] |
| TraesCS5B02G337000 | -3.55 | 0.351769028 | 7.09 | 3.37E-06 | + | 1326 | protein_coding | putrescine hydroxycinnamoyltransferase-like [Aegilops tauschii subsp. tauschii] |
| novel.12863 | 3.55 | 0.097566348 | -7.8 | 2.67E-05 | + | 3595 | - | uncharacterized protein LOC109734495 [Aegilops tauschii subsp. tauschii] |
| TraesCS6D02G080500 | NA | NA | 7.13 | 1.10E-06 | - | 1713 | protein_coding | protein SAR DEFICIENT 1-like isoform X1 [Aegilops tauschii subsp. tauschii] |
| TraesCS5B02G306100 | NA | NA | 7.2 | 1.38E-06 | - | 761 | protein_coding | hypothetical protein BRADI_4g35430v3 [Brachypodium distachyon] |
| TraesCS1D02G415300 | -2.8 | 7.91E-16 | -7 | 3.91E-07 | + | 925 | protein_coding | uncharacterized protein LOC109761946 [Aegilops tauschii subsp. tauschii] |
| TraesCS2B02G184000 | NA | NA | 9.17 | 1.08E-06 | + | 1675 | protein_coding | phloretin 2'-O-glucosyltransferase-like [Aegilops tauschii subsp. tauschii] |
| TraesCS4A02G082200 | NA | NA | 7.18 | 1.61E-06 | + | 1843 | protein_coding | serine/threonine-protein kinase WAG1-like [Aegilops tauschii subsp. tauschii] |
| TraesCS2A02G377400 | NA | NA | 8.5 | 0.000239852 | + | 1383 | protein_coding | probable metal-nicotianamine transporter YSL13 [Brachypodium distachyon] |
| TraesCS7B02G437300 | 2.32 | NA | -21 | NA | - | 4429 | protein_coding | putative disease resistance RPP13-like protein 3 [Panicum hallii] |
| TraesCS3D02G094400 | NA | NA | 7.25 | 8.28E-07 | + | 667 | protein_coding | uncharacterized protein LOC109769232 [Aegilops tauschii subsp. tauschii] |
| TraesCS6A02G296600 | NA | NA | 7.45 | 1.01E-06 | + | 1653 | protein_coding | protein SAR DEFICIENT 1-like [Aegilops tauschii subsp. tauschii] |
| TraesCS3A02G488200 | -1.99 | 0.572556827 | 8.49 | 1.02E-06 | - | 915 | protein_coding | glutathione S-transferase U8-like [Aegilops tauschii subsp. tauschii] |
| TraesCS4D02G030700 | NA | NA | 8.81 | 2.75E-11 | - | 675 | protein_coding | oxalate oxidase precursor [Triticum aestivum] |
| TraesCS3B02G529300 | -3.44 | 0.212974628 | 7.47 | 0.000769392 | - | 1008 | protein_coding | glucan endo-1,3-beta-glucosidase GII-like [Aegilops tauschii subsp. tauschii] |
| TraesCS3B02G015800 | -2.01 | 0.002116135 | -7.7 | 6.06E-07 | + | 528 | protein_coding | unnamed protein product [Triticum aestivum] |
| TraesCS7D02G219000 | NA | NA | 7.05 | 9.61E-06 | - | 3038 | protein_coding | uncharacterized protein LOC109785175 [Aegilops tauschii subsp. tauschii] |
| TraesCS6D02G310700 | -1.39 | 0.663459605 | 7.24 | 6.33E-05 | + | 1331 | protein_coding | flavonol synthase/flavanone 3-hydroxylase-like [Aegilops tauschii subsp. tauschii] |
| TraesCS7A02G155000 | NA | NA | 7.59 | 2.50E-07 | - | 1811 | protein_coding | cytochrome P450 709B1-like [Aegilops tauschii subsp. tauschii] |
| TraesCS1A02G435200 | -3.23 | 0.416250247 | 7.89 | 6.34E-08 | + | 727 | protein_coding | calmodulin-like protein 3 [Aegilops tauschii subsp. tauschii] |
| novel.2568 | -2.7 | 0.316252555 | 8.15 | 2.66E-09 | + | 497 | - | -- |
| novel.10050 | 6.99 | NA | -21 | NA | + | 1984 | - | NADPH-dependent diflavin oxidoreductase 1-like [Aegilops tauschii subsp. tauschii] |
| TraesCS2B02G396000 | -0.66 | 0.678537087 | -7.4 | 0.057726024 | + | 1462 | protein_coding | GA2ox-A6 [Triticum aestivum] |
| TraesCS3A02G437900 | NA | NA | 7.21 | 2.43E-07 | + | 904 | protein_coding | putative glutathione S-transferase GSTU6 [Triticum urartu] |
| TraesCS2D02G316300 | -3.23 | 0.416250247 | 7.37 | 1.06E-06 | + | 913 | protein_coding | unnamed protein product [Triticum aestivum] |
| TraesCS5B02G398800 | -5.78 | 0.001109145 | 9.06 | 1.61E-11 | + | 1670 | protein_coding | UDP-glycosyltransferase 83A1-like [Aegilops tauschii subsp. tauschii] |
| TraesCS4D02G128300 | NA | NA | 7.43 | 0.003231368 | - | 1898 | protein_coding | cyanidin 3-O-rutinoside 5-O-glucosyltransferase-like [Aegilops tauschii subsp. tauschii] |
| TraesCS6D02G165300 | -2.82 | 0.48083833 | 7.27 | 5.45E-07 | + | 147 | protein_coding | hypothetical protein TRIUR3_24014 [Triticum urartu] |
| TraesCS3D02G496600 | -4.43 | 0.088017071 | 7.31 | 1.31E-06 | - | 866 | protein_coding | probable NADPH:quinone oxidoreductase 1 [Aegilops tauschii subsp. tauschii] |
| TraesCS2A02G486100 | NA | NA | 8.39 | 2.25E-09 | + | 750 | protein_coding | protein DOG1-like 4 [Aegilops tauschii subsp. tauschii] |
| novel.4928 | NA | NA | 8.69 | 2.24E-10 | + | 1555 | - | -- |
| TraesCS1D02G401500 | NA | NA | 7.7 | 0.048824783 | + | 267 | protein_coding | CLAVATA3/ESR (CLE)-related protein 2-like [Aegilops tauschii subsp. tauschii] |
| TraesCS7A02G083700 | NA | NA | 7.09 | 3.08E-06 | + | 729 | protein_coding | probable glutathione S-transferase [Aegilops tauschii subsp. tauschii] |
| TraesCS3D02G257400 | NA | NA | 7.29 | 5.31E-07 | + | 1434 | protein_coding | UDP-glycosyltransferase 88B1-like [Aegilops tauschii subsp. tauschii] |
| TraesCS4D02G227200 | NA | NA | 7.43 | 3.87E-07 | - | 1539 | protein_coding | hypothetical protein BRADI_1g68350v3 [Brachypodium distachyon] |
| TraesCS1D02G024800 | 5.7 | NA | -7.2 | NA | - | 1972 | protein_coding | uncharacterized protein LOC109741991 [Aegilops tauschii subsp. tauschii] |
| TraesCS2B02G509700 | NA | NA | 7.31 | 8.72E-08 | + | 1868 | protein_coding | unnamed protein product [Triticum aestivum] |
| TraesCS4D02G030800 | NA | NA | 8.32 | 9.03E-10 | + | 675 | protein_coding | Oxalate oxidase 2 [Triticum urartu] |
| TraesCS7D02G432800 | NA | NA | 7.62 | 2.13E-06 | + | 1443 | protein_coding | DIBOA-glucoside dioxygenase BX6-like [Aegilops tauschii subsp. tauschii] |
| TraesCS4D02G032000 | NA | NA | 8.54 | 1.28E-09 | + | 675 | protein_coding | germin [Triticum aestivum] |
| TraesCS3B02G471900 | NA | NA | 7.98 | 1.76E-08 | + | 971 | protein_coding | unnamed protein product [Triticum aestivum] |
| TraesCS1D02G190500 | -3.37 | 0.396193665 | 8.03 | 3.07E-08 | + | 962 | protein_coding | probable glutathione S-transferase GSTU6 [Aegilops tauschii subsp. tauschii] |
| TraesCS3B02G598200 | NA | NA | 8.08 | 3.15E-09 | - | 1806 | protein_coding | unnamed protein product [Triticum aestivum] |
| TraesCS2D02G332300 | NA | NA | 8.43 | 9.05E-10 | - | 2709 | protein_coding | unnamed protein product [Triticum aestivum] |
| TraesCS1B02G285400 | NA | NA | 7.89 | 3.71E-08 | - | 1472 | protein_coding | glucan endo-1,3-beta-glucosidase 14-like isoform X1 [Aegilops tauschii subsp. tauschii] |
| TraesCS4B02G178300 | -2.31 | 0.566462963 | 7.04 | 2.20E-07 | - | 1870 | protein_coding | uncharacterized protein LOC109762516 [Aegilops tauschii subsp. tauschii] |
| TraesCS6B02G074100 | -3.51 | 0.091370726 | 7.34 | 3.65E-08 | + | 1614 | protein_coding | cytochrome P450 76M5-like [Aegilops tauschii subsp. tauschii] |
| TraesCS4B02G033100 | NA | NA | 7.88 | 4.89E-09 | - | 675 | protein_coding | Oxalate oxidase 2 [Triticum urartu] |
| novel.2182 | NA | NA | 8.12 | 0.000273731 | - | 796 | - | pre-mRNA-splicing factor 18-like [Aegilops tauschii subsp. tauschii] |
| TraesCS7B02G019300 | NA | NA | 8.15 | 0.000249963 | + | 1279 | protein_coding | uncharacterized protein LOC109754927 [Aegilops tauschii subsp. tauschii] |
| novel.4176 | NA | NA | 8.02 | 1.53E-07 | + | 1607 | - | unnamed protein product [Triticum aestivum] |
| TraesCS5A02G531600 | NA | NA | 7.58 | 8.57E-08 | + | 2543 | protein_coding | protein PAIR1-like [Aegilops tauschii subsp. tauschii] |
| novel.826 | NA | NA | 7.84 | 3.00E-08 | + | 1869 | - | brassinosteroid LRR receptor kinase 1 [Triticum aestivum] |
| novel.7864 | NA | NA | 7.02 | 1.81E-05 | + | 1477 | - | Extended synaptotagmin-1 [Triticum urartu] |
| TraesCS5B02G478200 | NA | NA | 7.95 | 2.27E-09 | - | 866 | protein_coding | hypothetical protein [Triticum aestivum] |
| TraesCS4A02G090200 | 3.82 | NA | -20 | NA | - | 1650 | protein_coding | Peroxidasin-like protein [Triticum urartu] |
| TraesCS6D02G244600 | -2.85 | 0.453277978 | 7.36 | 5.38E-06 | - | 1777 | protein_coding | 7-deoxyloganetic acid glucosyltransferase-like [Aegilops tauschii subsp. tauschii] |
| TraesCS3A02G488300 | NA | NA | 7.43 | 3.16E-06 | - | 573 | protein_coding | glutathione S-transferase U8-like [Aegilops tauschii subsp. tauschii] |
| TraesCS3A02G084700 | NA | NA | 7.01 | 9.17E-06 | + | 1785 | protein_coding | unnamed protein product [Triticum aestivum] |
| TraesCS7D02G418200 | -3.23 | 0.416250247 | 7.12 | 4.27E-06 | - | 2810 | nontranslating_CDS | wall-associated receptor kinase 1-like [Aegilops tauschii subsp. tauschii] |
| TraesCS2D02G093000 | NA | NA | 7.09 | 1.21E-05 | + | 1225 | protein_coding | unnamed protein product [Triticum aestivum] |
| TraesCS3B02G099700 | NA | NA | 7.16 | 5.34E-06 | - | 1806 | protein_coding | unnamed protein product [Triticum aestivum] |
