# Supplementary material for: Comparative transcriptomic and metabolic profiling provides insight into the mechanism by which the autophagy inhibitor 3-MA enhances salt stress sensitivity in wheat seedlings
Source: BMC Plant Biol. 2021 Dec 6;21:577. doi: 10.1186/s12870-021-03351-5 (PMC8647401; doi:10.1186/s12870-021-03351-5)
Supplement: Supplementary file 14 — Additional file 14: Supplementary Figure 4. Principal component analysis (PCA) score diagram in (A) negative, (B) positive ion mode of metabolic profiles in the wheat roots and leaves. [file 12870_2021_3351_MOESM14_ESM.docx]

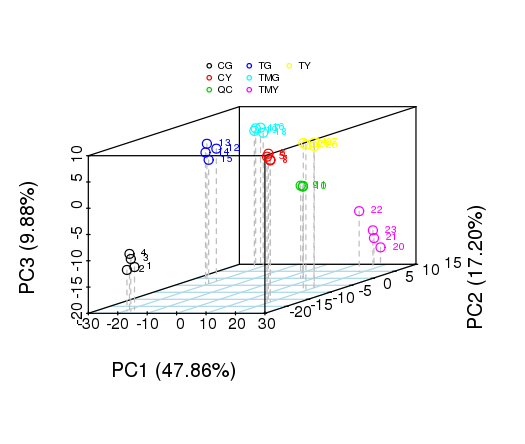

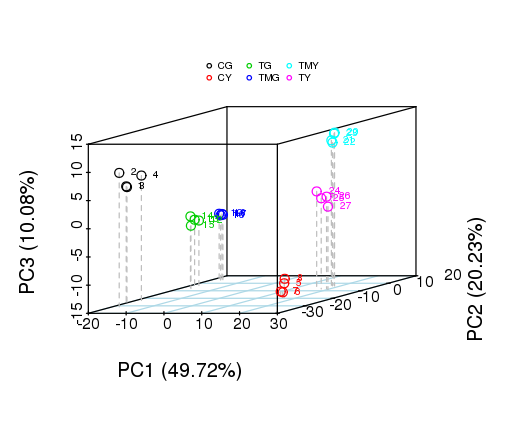


B

A

Supplementary Figure 4 Principal component analysis (PCA) score diagram in (A) negative, (B) positive ion mode of metabolic profiles in the wheat roots and leaves

Note: CG: the control wheat roots, TG: 150 mM NaCl treated wheat roots, TMG: 5 mM 3-MA + 150 mM NaCl treated wheat roots, CY: the control wheat leaves, TY: 150 mM NaCl treated wheat leaves, TMY: 5 mM 3-MA + 150 mM NaCl treated wheat leaves. PC1, the first principal component, PC2, the second principal component. The scatter points of different colors represent the samples of different experimental groups, and the ellipse is the 95% confidence interval.
