# Supplementary material for: Comparative transcriptomic and metabolic profiling provides insight into the mechanism by which the autophagy inhibitor 3-MA enhances salt stress sensitivity in wheat seedlings
Source: BMC Plant Biol. 2021 Dec 6;21:577. doi: 10.1186/s12870-021-03351-5 (PMC8647401; doi:10.1186/s12870-021-03351-5)
Supplement: Supplementary file 15 — Additional file 15: Supplementary Figure 5. Volcano plot of negative ion and positive ion mode in roots and leaves of wheat seedlings. [file 12870_2021_3351_MOESM15_ESM.docx]

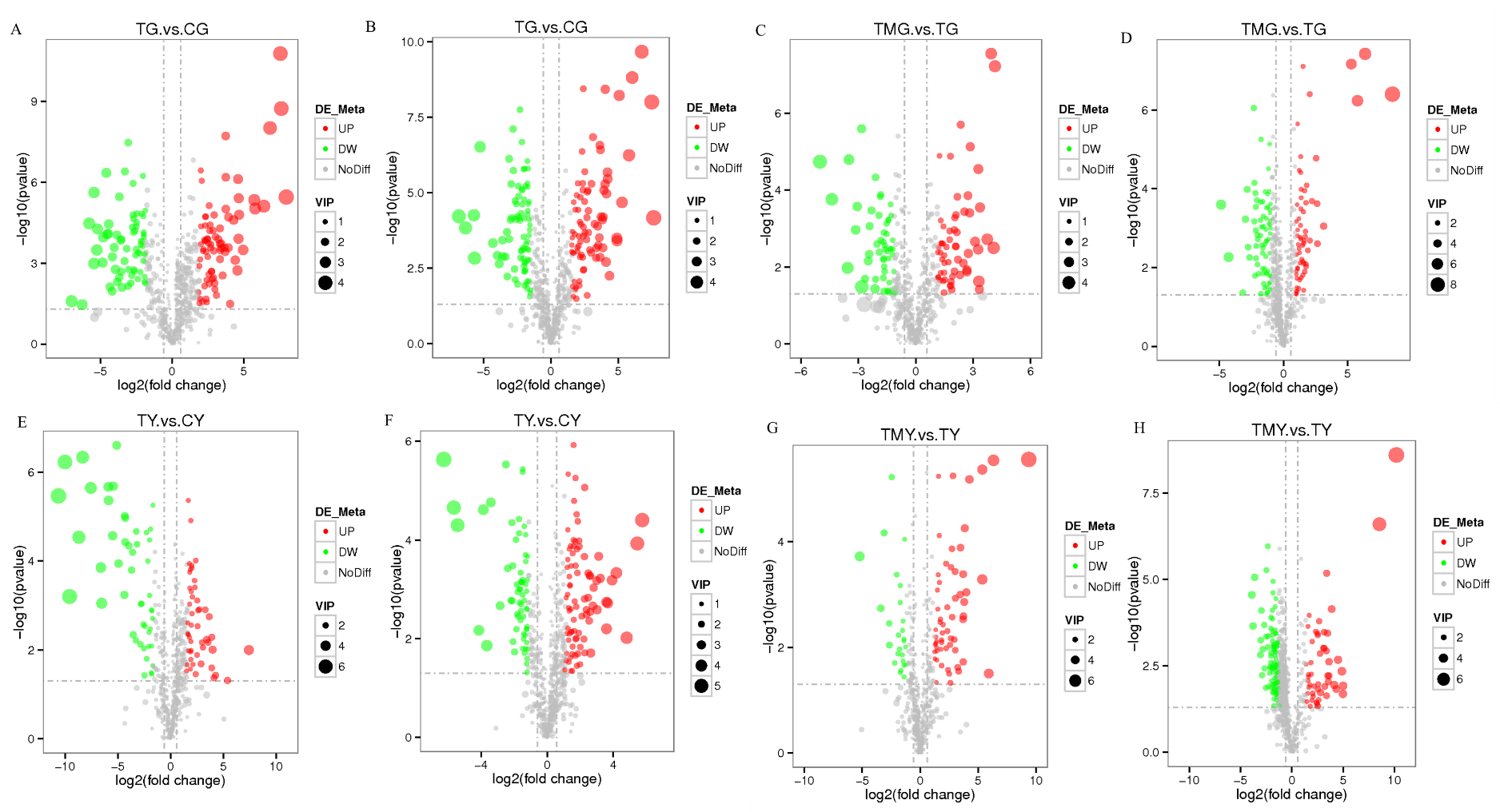


Supplementary Figure 5 Volcano plot of negative ion and positive ion mode in roots and leaves of wheat seedlings

Note: A, the negative ion mode of CG.vs.TG, B, the positive ion mode of CG.vs.TG, C, the negative ion mode of TMG.vs.TG, D, the positive ion mode of TMG.vs.TG, E, the negative ion mode of CY.vs.TY, F, the positive ion mode of CY.vs.TY, G, the negative ion mode of TMY.vs.TY, H, the positive ion mode of TMY.vs.TY.
