# Supplementary material for: Comparative transcriptomic and metabolic profiling provides insight into the mechanism by which the autophagy inhibitor 3-MA enhances salt stress sensitivity in wheat seedlings
Source: BMC Plant Biol. 2021 Dec 6;21:577. doi: 10.1186/s12870-021-03351-5 (PMC8647401; doi:10.1186/s12870-021-03351-5)
Supplement: Supplementary file 21 — Additional file 21: Supplementary Figure 6. The effect of 3-MA on activity of POD, SOD, CAT and GABA content in wheat seedlings under NaCl stress. [file 12870_2021_3351_MOESM21_ESM.docx]

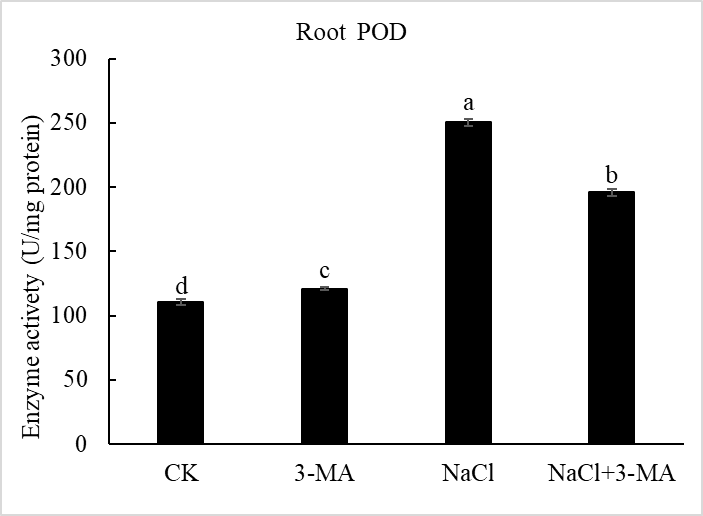

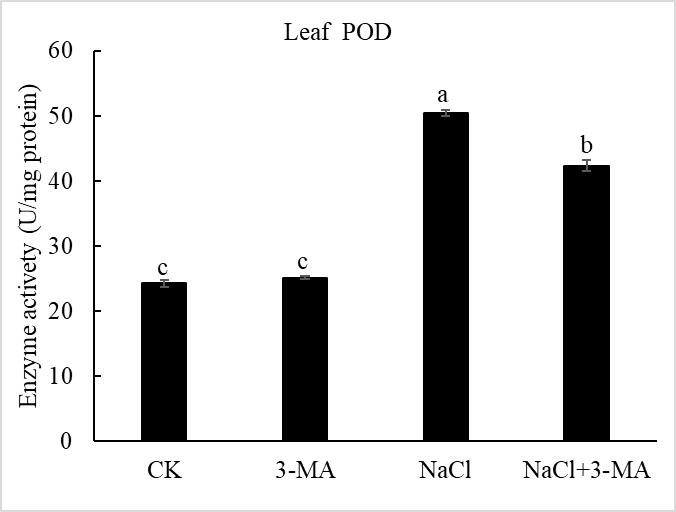


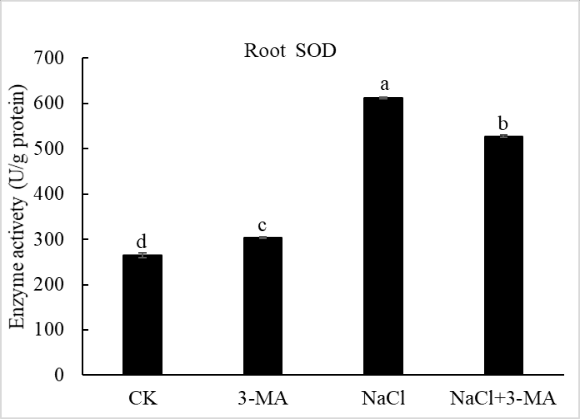

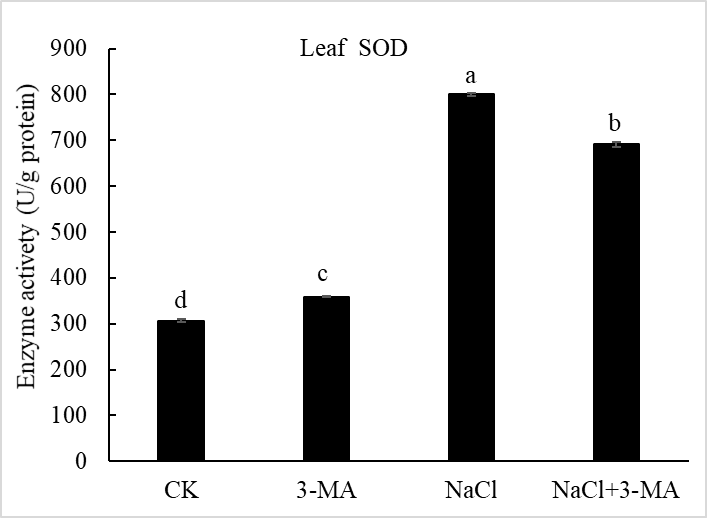


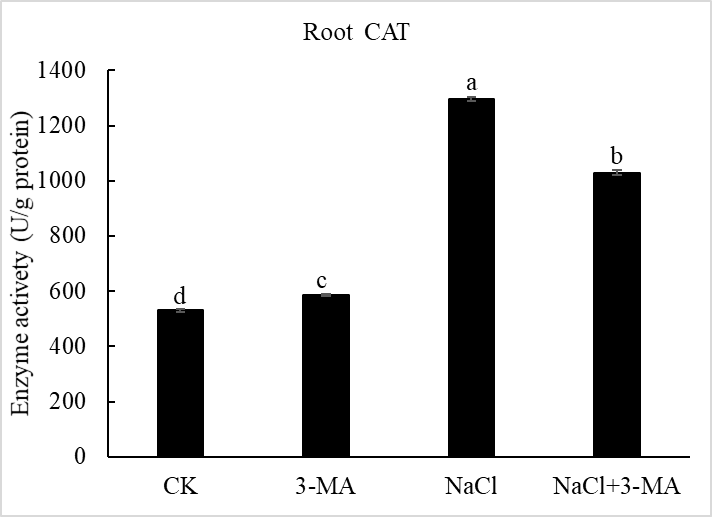

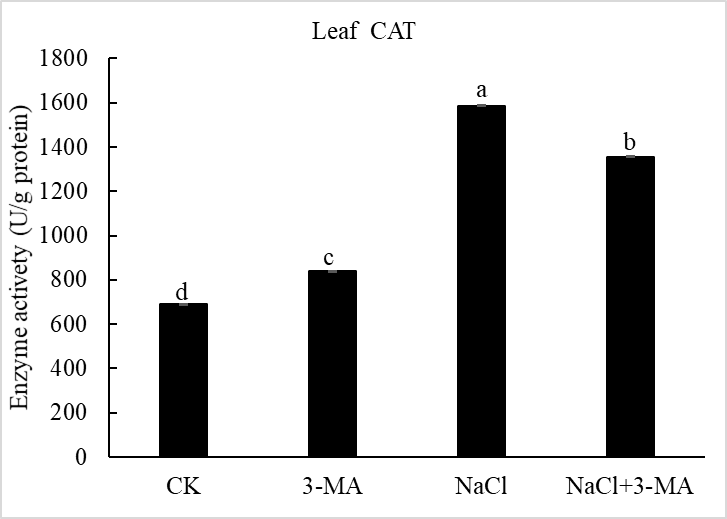


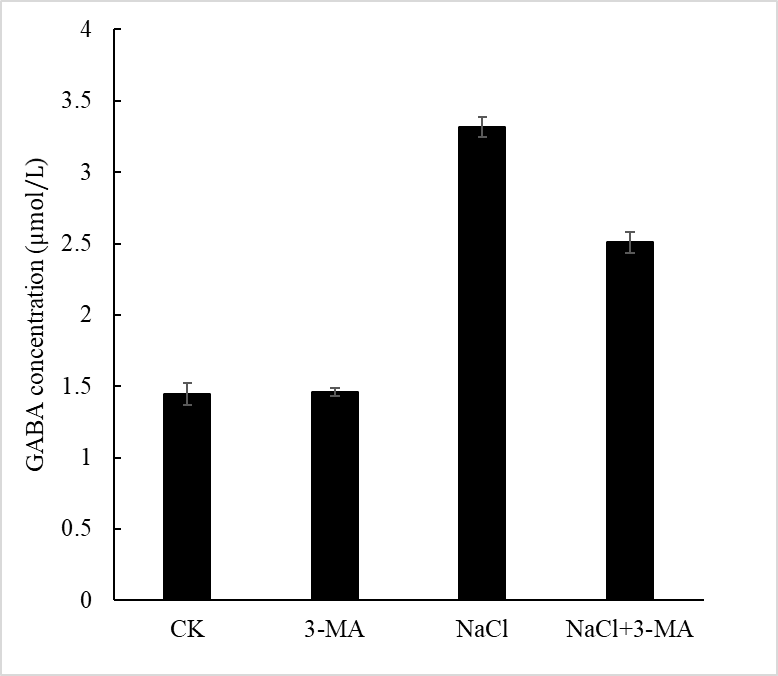


Leaf GABA

a

b

c

c

Supplementary Figure 6 Figure 3 The effect of 3-MA on activity of POD, SOD, CAT and GABA content in wheat seedlings under NaCl stress

All of the experiments presented here were performed at least 3 times, and similar results were obtained. Bars with different letters are significantly different at *P* < 0.05.
